# Preliminary Data on SNP of Transplantation-Related Genes after Haploidentical Stem Cell Transplantation

**DOI:** 10.3390/jcm13164681

**Published:** 2024-08-09

**Authors:** Ching-Ping Tseng, Tung-Liang Lin, Shu-Hui Tsai, Wei-Tzu Lin, Fang-Ping Hsu, Wei-Ting Wang, Ding-Ping Chen

**Affiliations:** 1 Department of Laboratory Medicine, Chang Gung Memorial Hospital, Taoyuan 33305, Taiwan; ctseng@mail.cgu.edu.tw (C.-P.T.); h22183@cgmh.org.tw (S.-H.T.); berry0908@cgmh.org.tw (W.-T.L.); sfp721@cgmh.org.tw (F.-P.H.); s1223@cgmh.org.tw (W.-T.W.); 2 Department of Medical Biotechnology and Laboratory Science, College of Medicine, Chang Gung University, Taoyuan 33305, Taiwan; 3 Graduate Institute of Biomedical Sciences, College of Medicine, Chang Gung University, Taoyuan 33305, Taiwan; 4 Division of Hematology, Department of Internal Medicine, Chang Gung Memorial Hospital, Taoyuan 33305, Taiwan; ldl2605@cgmh.org.tw

**Keywords:** human leukocyte antigen-related genes, non-HLA genes, single nucleotide polymorphisms, graft-versus-host disease, effectiveness

## Abstract

**Background**: Hematopoietic stem cell transplantation (HSCT) is one of the mainstream treatments for patients with hematologic malignancies. The matching status of human leukocyte antigen (HLA) between the donor and recipient is highly related to the outcomes of HSCT. Haploidentical HSCT (haplo-HSCT) has emerged as a type of HSCT for patients who cannot find a fully HLA-matched donor. In this study, we investigated whether the single nucleotide polymorphisms (SNPs) of the HLA-related genes and the genes encoding co-stimulatory molecules located on the non-HLA region are related to the outcomes of haplo-HSCT. **Methods**: The genomic DNAs of 24 patients and their respective donors were isolated from the peripheral blood obtained before performing haplo-HSCT. A total of 75 SNPs of the HLA-related genes (HCP5, NOTCH4, HLA-DOA, LTA, HSPA1L, BAG6, RING1, TRIM27, and HLA-DOB) and the genes located in the non-HLA genes involved in co-stimulatory signaling (CTLA4, TNFSF4, CD28, and PDCD1) were selected to explore their relationship with the outcomes after haplo-HSCT, including graft-versus-host disease, survival status, and relapse. **Results**: Our data revealed that specific donor or patient SNPs, including rs79327197 of the HLA-DOA gene, rs107822 and rs213210 of the RING1 gene, rs2523676 of the HCP5 gene, rs5742909 of the CTLA4 gene, rs5839828 and rs36084323 of the PDCD1 gene, and rs1234314 of the TNFSF4 gene, were significantly related to the development of adverse outcomes post-haplo-HSCT. **Conclusions**: These SNPs may play important roles in post-transplant immune response that can be considered during the selection of suitable donors.

## 1. Introduction

Hematopoietic stem cell transplantation (HSCT) is a procedure involving the administration of healthy hematopoietic stem cells to patients with dysfunctional or depleted bone marrow, with an aim to restore the bone marrow function and reconstruct the immune system [1]. In principle, it is preferential to have human leukocyte antigens (HLAs) that are fully matched between recipient and donor to avoid the occurrence of graft-versus-host disease (GVHD) [1,2,3]. Given the declining birth rate, it becomes more difficult to find a suitable donor to perform HLA-matched HSCT [4]. Haploidentical HSCT (haplo-HSCT) is a type of HSCT where the donor matches half of the recipient HLA. The hematopoietic stem cells for haplo-HSCT are mainly obtained from patient’s parents, siblings, and children. Although haplo-HSCT greatly improves the chances of transplantation for patients [5] with an average of 2.7 potential donors per patient, the strong bidirectional alloreactivity results in an unacceptably high rate of graft rejection and GVHD that hinders its initial use in the clinical setting. Haplo-HSCT becomes more feasible for therapeutic purpose after the advancement of immunomodulatory medical technology and the development of T-cell depletion techniques [6]. The transplantation efficacy of haplo-HSCT and the survival rate of patients are now comparable to the HSCT with HLA-matched sibling or unrelated donors [7,8]. Haplo-HSCT is nowadays considered as a therapeutic option for patient.

Despite the success rate of haplo-HSCT greatly increasing in the clinical setting, cases with adverse outcomes still occur for patients with haplo-HSCT. Genetic variants of recipients and/or donors have been linked to the development of GVHD, the survival status, and relapse for patients receiving HLA-matched transplants. Specifically, the single nucleotide polymorphisms (SNPs) of the genes located in the HLA region (such as HCP5, NOTCH4, HLA-DOA, LTA, HSPA1L, BAG6, RING1, TRIM27, and HLA-DOB) and the genes located in the non-HLA region involving in co-stimulatory signaling of T-cells (such as CTLA4, CD28, TNFSF4, and PDCD1) are related to the outcomes of HLA-matched HSCT [9,10,11]. Patients receiving the graft with more genetic implications for positive post-transplant outcomes are likely to have a better prognosis.

Most studies on the relationship between haplo-HSCT and genetic polymorphism focus on the gene encoding human killer cell immunoglobulin-like receptors (also known as CD158), a family of highly polymorphic activating and inhibitory receptors that serve as key regulators of human NK cell function [12,13,14]. In this study, we aimed to address whether polymorphisms of HLA-related genes and the genes involving in co-stimulatory signaling of T-cells located in the non-HLA region have any effects on the outcomes of haplo-HSCT. The new insights provided in this study may lead to the development of a better strategy to identify suitable haplo-HSCT donors.

## 2. Materials and Methods

### 2.1. Subjects

The Institutional Review Board of Chang Gung Memorial Hospital reviewed and approved this study with the approval ID of 102-4949B, 202101454B0, and 202300738B0. A total of 24 patients receiving haplo-HSCT with the post-transplant cyclophosphamide (PT/Cy) regimen and their respective donors were enrolled in this study. The clinical characteristics of these patients are detailed in the Results section. All donors and recipients signed informed consent forms. The use of patients’ materials and all research methods including genetical tests were conducted in accordance with ethical requirements and regulations of Chang Gung Memorial Hospital.

### 2.2. Assessment of the Outcomes after Haplo-HSCT

End points of interest were acute and chronic GVHD, relapse, and survival status (alive or death) assessed at the end of the study (5 May 2023). The SNPs that were significantly associated with the outcomes were then subject to event-free survival analysis. The status and grading of GVHD were reviewed and assessed by a senior hematologist according to the statements by the Center for International Blood and Marrow Transplant Research (CIBMTR) [15]. Briefly, to be defined as acute GVHD (aGVHD), one must present with acute symptoms until day +100 after HSCT, primarily affecting the skin, or liver, or gastrointestinal tract. Special grading for different clinical forms is used. Grade I: maculopapular rash over <25% of body area with no liver or gastrointestinal involvement; Grade II: maculopapular rash over 25% to 50% of body area, diarrhea > 500 mL/day, and bilirubin 2 to 6 mg/dL; Grade III: maculopapular rash over >50% of body area, and severe diarrhea; Grade IV: skin blisters, bilirubin > 15 mg/dL, severe diarrhea with pain, and life-threatening [16]. In SNP analysis, the severity of aGVHD was classified as mild (Grades I–II) and severe (Grades III–IV), respectively. Chronic GVHD (cGVHD) was defined as the features of the disease that can affect any organ in the body without time limit on diagnosis. It can be further classified into classical cGVHD and overlap cGVHD (with aGVHD).

Relapse was defined as morphologic evidence of the disease in peripheral blood, bone marrow, or extramedullary sites, or recurrence and persistence of pretransplant chromosomal abnormalities [17]. Survival status (alive or death) from any cause was assessed at the end of the study. Event-free survival was defined as the time from stem cell infusion to the occurrence of adverse outcomes or death.

### 2.3. Selection of Candidate SNPs

Based on the study by Petersdorf et al. [18] and our study for the association of SNPs in the HLA-related genes with the outcomes of HSCT [9,10], 41 candidate SNPs of 9 HLA-related genes (Table 1) were selected for analysis by donor, recipient, and mismatch group, respectively. In addition, 34 candidate SNPs of the four genes encoding co-stimulatory molecules on T cells (CTLA4, CD28, TNFSF4, and PDCD1) located in the non-HLA region [11] were selected for analysis (Table 2). For the genes encoding co-stimulatory molecules, only donor SNPs were analyzed because donor T cells reconstitutes in patient’s bone marrow and peripheral blood after transplantation, while the recipient T cells were eliminated by chemotherapy or radiation therapy before haplo-HSCT.

### 2.4. Sample Collection and SNP Analysis

Before performing haplo-HSCT, the peripheral blood (3 mL) from patients and their corresponding donors was collected into the blood collection tube containing the anticoagulant ethylenediaminetetraacetic acid for extraction of genomic DNA by using QIAamp DNA Blood Mini Kit (Qiagen, Valencia, CA, USA). The specific gene regions covering the candidate SNPs were amplified by the polymerase chain reaction (PCR) using the MJ Research PTC-200 Thermal Cycler (Waltham, MA, USA). The PCR mix included 1 μL each of forward and reverse primer (10 μM) 12.5 μL of 2X HotStart PCR Mix (BIOMAN, Taipei, Taiwan), 1 μL of DNA sample, and 9.5 μL of water. The primer pairs for amplifying the DNA fragments covering the candidate SNPs and the respective PCR programs are shown in Table 3. PCR products were visualized by fractionating on a 1.5% agarose gel electrophoresis. The PCR products with the correct size were isolated and sequenced using the Big Dye Terminator Cycle Sequencing kit (Thermo Fisher, Waltham, MA, USA) along with an ABI PRISM Genetic Analyzer (Thermo Fisher, Waltham, MA, USA) according to the manufacturer’s instructions. Due to limited available DNA and occasional failure of PCR, not all samples had complete sets of SNP data. The SNPs of HLA-related genes were divided into 3 groups—donor, patient, and donor–patient matching pairs—for analysis according to the study by Petersdorf et al. [18]. Only donor SNPs were analyzed for the genes which encode co-stimulatory molecules and locate in the non-HLA region.

### 2.5. Statistical Analysis

Univariate analysis was performed to compare the frequency of the genotypes between patients with and without occurrence of the indicated outcomes including aGVHD/cGVHD, relapse, and dead at the end of the study. The frequencies of alleles or genotypes for patients with the specified outcomes and those without were compared using the chi-square or Fisher’s exact test. Fisher’s exact test was further performed if more than 20% of the cells had expected values less than 5 in the chi-square test. The analysis considered five inheritance modes: additive (AA vs. Aa vs. aa), recessive (AA + Aa vs. aa), dominant (AA vs. Aa + aa), heterozygous (AA vs. Aa), and homozygous (AA vs. aa). Briefly, “A” represented the allele with high frequency, while the other allele was referred as the minor allele “a”. The additive model is determined by the combined effects of multiple genes with each gene contributing a small amount to the overall phenotype. In the recessive model, an allele only expresses its phenotype when present in the homozygous state. In the dominant model, an allele expresses its phenotype even when present in the heterozygous state (Aa). In the heterozygous model, a heterozygote genotype is in some way superior to that of homozygote genotype. In the homozygous model, the phenotype of the heterozygote falls between those of the two homozygotes, reflecting the relative effects of the two alleles.

For the SNPs which had the most significant associations with the outcomes following analyses as described above, event-free survival was analyzed using the Kaplan–Meier method and assessed with the log-rank test. Patients who were alive without presenting the end points of interest at the end of the study or at the last follow-up were censored. *p* < 0.05 was considered statistically significant. The linkage disequilibrium (LD), the pairwise linkage disequilibrium value D’, and the haplotype blocks of SNPs were determined by using the Haploview 4.2 software [19]. The haplotype blocks were defined as the SNPs in this region that had no evidence of historical recombination [20].

## 3. Results

### 3.1. Patient Characteristics

The clinical characteristics of patients are shown in Table 4. Twenty-four patients (12 males and 12 females) receiving haplo-HSCT were enrolled in this study, including 17 patients (70.8%) with AML and 7 patients (29.2%) with ALL. The median age of these patients at transplantation was 43.9 years old with a range from 8 months to 67 years old. Donors were mainly from the haploidentical siblings (50%), followed by the offspring (25%) and parents (16.7%) of the patients. Only two patients (8.3%) received haploidentical transplants from the unrelated donors. Among the 24 patient–donor pairs, 33.3% were female recipients with male donors (M → F), 29.2% were male recipients with male donors (M → M), 16.7% were female recipients with female donors (F → F), and 20.8% were male recipients with female donors. Overall, 45.9% of pairs were sex matched: 58.3% among male recipients and 33.3% among female recipients. Fifteen patient–donor pairs (62.5%) were positive for CMV serum test. All patients received the stem cells collected from the peripheral blood of the donors. Before transplantation, 10 patients (42%) received myeloblative conditioning (MAC) and 14 patients (58%) received reduced intensity conditioning (RIC). Busulfan was used in combination with cyclophosphamide (Cy) for MAC. Fludarabibne (Flu) was used in combination with Cy for RIC. All patients received post-transplant Cy for GVHD prophylaxis. The median follow-up among survivors was 29 months (range 6–99). The detailed clinical characteristics for each patient are shown in Table S1.

### 3.2. Clinical Outcomes of Patients Receiving Haplo-HSCT

The post-haplo-HSCT clinical outcomes of patients were analyzed at the end of the study (Table 5). Thirteen (54.2%) and eleven (45.8%) of the 24 patients were alive and dead, respectively. Sixteen (66.7%) patients developed relapse during the follow-up. Fourteen (58.3%) and four (16.7%) patients developed aGVHD and cGVHD during the follow-up, respectively. Of the patients with aGVHD, eight patients (33.3%) developed grade I or II, while six patients (25.0%) developed grade III or IV, respectively. GVHD was not developed for six (25.0%) patients. Other complications were developed post-haplo-HSCT including six patients (25.0%) with BK virus-related hemorrhagic cystitis, one with myelitis (4.1%), two with secondary graft rejection (8.2%), and one with septic shock and congestive heart failure (4.1%), respectively. No other complications were observed for 14 patients (58.3%).

### 3.3. Association of Recipient Genotypes with the Outcomes of Patients Receiving Haplo-HSCT

The association of recipient genotypes with the outcomes of patients receiving haplo-HSCT was analyzed. Two SNPs (rs79327197 in HLA-DOA and rs107822 in RING1) were associated with the survival status post-haplo-HSCT (Table 6). The rs79327197 genotype in the HLA-DOA gene was associated with patient survival based on the heterozygous model (AA vs. AG, *p* = 0.031). The rs107822 in the RING1 gene was associated with patient survival based on the dominant model (TT vs. CT + CC, *p* = 0.036). The complete data are shown in Table S2.

### 3.4. Association of Donor Genotypes with the Outcomes of Patients Receiving Haplo-HSCT

The association of donor genotypes with the outcomes of patients receiving haplo-HSCT was analyzed. Seven donor SNPs (rs5742909 in CTLA4, rs1234314 in TNFSF4, rs2523676 in HCP5, rs107822 and rs213210 in RING1, and rs36084323 and rs5839828 in PD1) were associated with the outcomes post-haplo-HSCT (Table 7). The rs5742909 of CTLA4 was associated with patient survival based on the heterozygous model (CC vs. CT, *p* = 0.041). The rs1234314 of TNFSF4 was associated with patient survival based on the dominant model (CC vs. CG + GG, *p* = 0.033), in which the graft with at least one G-allele decreased the odds of survival. The rs107822 of RING1 was associated with relapse and cGVHD. It was associated with relapse based on the additive (*p* = 0.047), dominant (TT vs. CT + CC, *p* = 0.014), and heterozygous models (*p* = 0.045). The rs36084323 in the PDCD1 gene was associated with relapse based on the additive model (*p* = 0.042). In addition, another SNP in the PDCD1 gene, rs5839828, was associated with relapse based on the additive model (*p* = 0.014), recessive model (*p* = 0.037), and homozygous model (*p* = 0.015). The rs2523676 of HCP5 was associated with GVHD based on the additive model (CC vs. CT vs. TT, *p* = 0.026). In addition, the gene polymorphisms of rs213210 of RING1 were associated with mild GVHD (GVHD I–II) and severe GVHD (GVHD III–IV) based on the additive model (GG vs. AG vs. AA, *p* = 0.045 and 0.031, respectively). The complete data are shown in Tables S2–S7.

For the SNPs that were significantly associated with the outcome post-haplo-HSCT, Kaplan–Meier curves analysis was performed to illustrate the effects of SNPs on event-free survival. Of the SNPs under analysis in various models, only rs5742909 under the heterozygous model of overall survival and rs107822 under the additive and heterozygous models of relapse-free survival did not reach statistically significant values (Figures S1–S3). All other SNPs analyzed in various models show significant difference in either overall survival, relapse-free survival, or GVHD-free survival.

### 3.5. Association of Mismatch between Donor and Recipient Genotypes with the Outcomes of Patients Receiving Haplo-HSCT

The association of mismatch between donor and recipient genotypes with the outcomes of patients receiving haplo-HSCT was analyzed. Among all SNPs, only rs107822 in the RING1 gene significantly affected the transplant outcome of recipients depending on whether there was a mismatch between the donor and recipient genotypes. There was an increased risk to relapse when patients had the same genotype of rs107822 with the donors (*p* = 0.006) (Table 8). The complete data are shown in Table S3.

### 3.6. Linkage Disequilibrium Analysis

**Figure 1:**
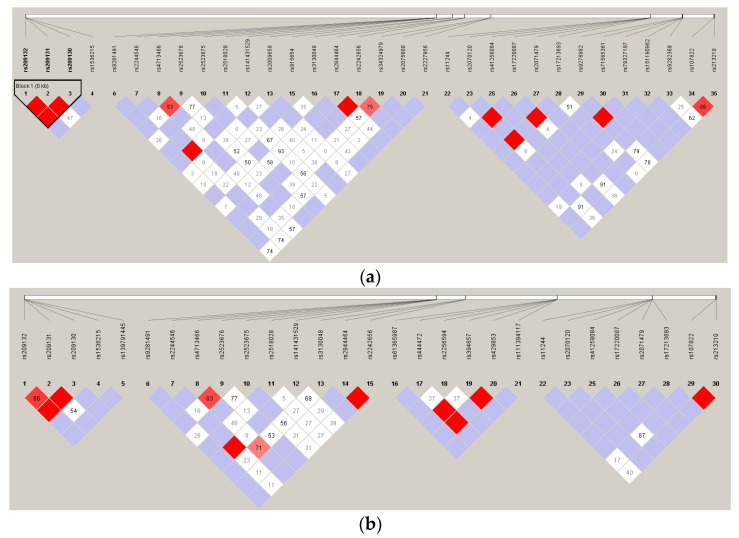

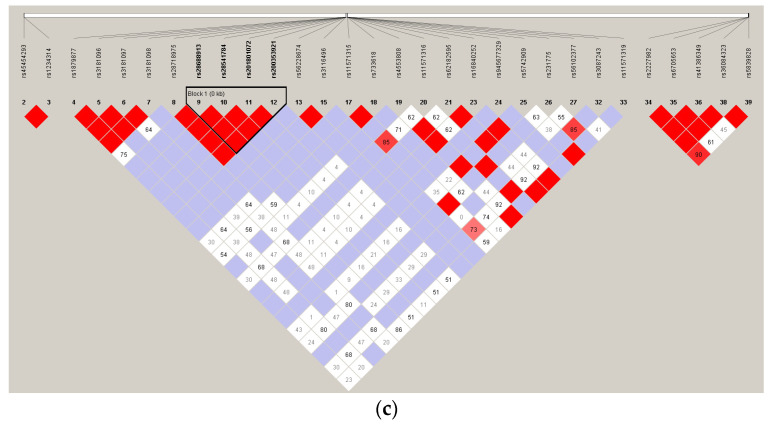
Linkage disequilibrium analysis of the candidate SNPs. (**a**) The SNPs from patients in HLA-related genes. (**b**) The SNPs from donors in HLA-related genes. (**c**) The SNPs from donors in non-HLA genes. The HLA-related genes from patients included TRIM27 gene, HCP5 gene, LTA gene, BAG6 gene, HSPA1L gene, RING1 gene, HLA-DOB gene, and HLA-DOA gene. The HLA-related genes from patients included TRIM27 gene, HCP5 gene, BAG6 gene, NOTCH4 gene, RING1 gene, and HLA-DOB gene. The non-HLA genes included CTLA4 gene, CD28 gene, PDCD1 gene, and TNFSF4 gene. The number in the boxes is the D’ value, which was measured between the pair of SNPs. The red color in the boxes indicates that the two SNPs had high linkage. The closer to white color indicates that the linkage decreases gradually. The light purple color indicates that the SNPs absolutely had no linkage. Due to the lack of evidence of recombination, a haplotype block was identified in this specific region.

The SNPs that were associated with the outcomes in patients were subject to LD analysis. LD analyses of the patient and donor SNPs in HLA-related genes and donor SNPs in co-stimulatory genes located in the non-HLA region are shown in Figure 1. One haplotype in TRIM27 gene containing the rs209132, rs209131, and rs209130 was defined for patient SNPs (Figure 1a). There was no haplotype block for donor SNPs in the HLA-related genes (Figure 1b). One haplotype in the CD28 gene containing the rs28688913, rs28541784, rs201801072, and rs200353921 was defined for patient SNPs (Figure 1c).

## 4. Discussion

The genetic polymorphisms of HLA-related genes and the immunity-related genes in the non-HLA region are known to associate with the outcomes of patients receiving fully HLA-matched donors [9,10,11]. In this study, we investigated further whether there is an association of the 41 SNPs in the HLA-related genes and 34 SNPs in the genes encoding co-stimulatory molecules on T cells with the outcomes (survival, relapse, and GVHD) for patients with AML and ALL receiving haploidentical transplants. Our data revealed that three SNPs in the HLA-related genes (HLA-DOA, HCP5, and RING1) and five SNPs in the genes encoding co-stimulatory molecules (CTLA4, TNFSF4, and PDCD1) in the non-HLA region were related to the outcomes of patients receiving haplo-HSCT.

Of the SNPs analyzed in the non-HLA region, rs5742909 of the CTLA4 gene, rs1234314 of the TNFSF4 gene, and rs36084323 and rs5839828 of the PDCD1 gene were significantly associated with the outcomes post-haplo-HSCT. CTLA4 is an immune checkpoint that is induced to express on the surface of T cells after T-cell activation and competes with CD28 to prevent sustained T-cell activation and avoid immune overreaction [21]. The heterozygous genotype of rs5742909 in the CTLA4 gene was associated with the survival of patients. All deceased patients received grafts from the donor with the CC genotype in rs5742909, while approximately 40% of the surviving patients received grafts with the CT genotype. A Taiwanese study also found that patients receiving grafts from the donor with TT genotype in rs5742909 have an increased risk of relapse in allogeneic HSCT [22]. The C-allele of rs5742909 was also relative to the occurrence of GVHD III-IV in CBT and the occurrence of cGVHD and GVHD in ALL patients after HSCT [13,14]. The SNP of rs5742909 is located at the promoter region of CTLA4. SNP mutations in the promoter may affect gene expression by altering promoter activity, transcription factor binding activity, DNA methylation, and histone modifications [23,24,25]. In our previous studies, it was found that rs5742909C>T reduced the transcription activity by 19% [26]. While the T-allele of rs5742909 had a higher odd of survival and relapse in haplo-HSCT and C-allele was a risk allele for chronic GVHD in ALL patients [11], it is speculated that the presence of C-allele in rs5742909 leads to a higher expression of CTLA4, causing donor T cells to attack host cells and cause GVHD. On the other hand, the presence of T-allele in rs5742909 likely leads to a lower expression of CTLA4, making worse the GVL effect and prone to relapse. In the study by Qin et al. [27], rs5742909 was not associated with the outcomes of haplo-HSCT, while the rs231775 was associated with GVHD and overall survival. We have a different definition of survival. We classified an individual as alive or dead at the end of the study, whereas their approach involves assessing the duration of survival. This disparity in definitions may contribute to the divergent outcomes in our and their results. In addition to univariant analysis for the association between SNPs of the genes under investigation and the outcomes of patients receiving haplo-HSCT, most of the SNPs effects on patient survival have been further confirmed by Kaplan–Meier curves analysis.

PDCD1 plays a key role in the regulation of the allogeneic immune response in transplantation. We found an association between rs36084323 and relapse in this haplo-HSCT study. The finding is consistent with our previous study of HLA-matched HSCT [11]. Notably, a study of Spanish population indicates that the donor’s rs36084323 genotype of PDCD1 gene was associated with GVHD II-IV but not with disease relapse for patients receiving HLA-matched HSCT [28]. This discrepancy may be attributed to the differences in genetic element in addition to the SNPs under study and/or treatment regimen between the two populations. Moreover, LAG-3 and PD-1 molecules have a synergistic effect on T-cell inhibition. Cruz et al. investigated the clinical outcomes after transplantation based on the combination of donor LAG-3 rs870849 and PDCD1 rs36084323 genotypes, and they suggested that the inhibitory effect of LAG-3 may be stronger than the negative signal driven by PD-1 [29]. Thus, the effect of rs36084323 on the relapse after haplo-HSCT needs to be further investigated. In addition to rs36084323, we also demonstrated that there is an association between rs5839828 of PDCD1 gene and the relapse of patients receiving haplo-HSCT. The current finding is consistent with our previous work showing an association between rs5839828 and relapse in HLA-matched HSCT which is the sole report for the association between rs5839828 and diseases thus far [11].

It is known that the interaction between TNFSF4 and OX40 plays a crucial role in Th17 regulation [30]. These checkpoints are implicated in the development of immune-related disorders, such as inflammation, autoimmune diseases, and tumors. In addition to being a susceptibility gene for HSCT transplantation, the polymorphisms of rs1234314 were also associated with systemic sclerosis, Sjögren’s syndrome, and allergic rhinitis [31,32,33]. The CC genotype of rs1234314 showed a correlation by protecting rhinitis [33]. Our results indicate that patients undergoing haplo-HSCT with donors possessing the CC genotype had a higher survival rate. This observation was likely associated with the rs1234314 variant, where the C variant exhibited higher relative light units compared to the G variant in the in vitro study of transcriptional activity using the luciferase reporter gene [34]. Therefore, we infer that the association of rs1234314 with the disease may be attributed to the impact of this SNP on gene transcriptional activity.

HCP5 is an HLA-related gene located within the HLA class I gene region and is a gene coding for a 317-bp non-coding RNA of a human endogenous retrovirus. Its transcripts were mainly composed of the 3′-long terminal repeat and pol sequences of human endogenous retrovirus-16 [35]. Dysregulation of lncRNAs has been reported to regulate the progression of malignancy in various types of cancer by acting as oncogenes or tumor suppressor genes [36]. A previous study also links HCP5 to the development of certain autoimmune diseases and cancers [35]. However, the defense and pathological functions of HCP5 as well as its structural and functional roles in RNA editing and signal transduction for epigenetic plasticity and immune response remain to be elucidated. The SNP of rs2523676 is located approximately 3 kbp from the 3′-end of HCP5. Although rs2523676 is not associated with the effectiveness of HSCT [9,10], it is in strong linkage disequilibrium with other SNPs [10]. Hence, the association of rs2523676 with the effectiveness of haplo-HSCT is likely due to its linkage disequilibrium with other SNPs associated with the outcomes of haplo-HSCT.

RING1 is a transcription factor that binds to specific DNA sequences to inhibit the expression of its targeted genes. RING1 is involved in multiple cellular functions including transcription, RNA metabolism, translation, cellular signaling, stress signaling, and cell cycle [37]. Dysfunction of RING1 may relate to the pathogenesis of autoimmune diseases, cancers, neurodegenerative diseases, and viral infections [38]. The SNP of rs107822 is also associated with the effectiveness of HSCT and CBT as reported in our previous studies [9,10,39]. Patients with at least one C-allele (CT+CC) of rs107822 have lower odds of survival. In addition, the donor with heterozygous genotype (CT) of rs107822 is associated with the risk of CMV infection and the occurrence of cGVHD in patients. Another SNP, rs213210, was previously found to be associated with the relapse of ALL patients after HSCT [9]. Both SNPs are in the RING1 promoter region. These SNPs may regulate RING1 expression that subsequently upregulates or downregulates the transcriptional activity of other genes [24,25,26] and affect the effectiveness of transplantation.

HLA-DOA participates in the T cell receptor (TCR) signaling pathway and nuclear factor of activated T-cells (NFAT) pathway in immune response [40]. We found that the SNPs of rs79327197 in the HLA-DOA gene of patient genome were associated with the survival for patients receiving haplo-HSCT. The SNP of rs79327197 was associated with the relapse of ALL patients and the survival of AML patients [11,18]. TCR signal pathway affects the activation and differentiation of T cells [41], and the NFAT pathway regulates the transcription of many cytokines, chemokines, and growth factors in immune cells [42]. This SNP is also located in the promoter region. We speculate that both SNPs may change the expression level of HLA-DOA, leading to the subsequent changes of humoral immune response, immune tolerance, and immune metabolism regulation.

There are some limitations in this study. The sample size is small, although it represents the number of cases collected from our hospital during the last seven years. In addition, the data reported in this study mainly represent the correlation of SNPs genotypes with the outcomes of haplo-HSCT. It is not necessary representing the relationship between cause and effect. Although common SNPs were found to associate with both HLA fully matched transplantation and haplo-HSCT, it is warranted to investigate further how SNPs affect transplant outcomes by using cell experiments or animal models to verify whether these SNPs have any effect on T-cell activation and signaling. Moreover, the cohort of the patient and donor pairs was mainly composed of Taiwanese persons. The data of this study obtained from ethnicity-restricted groups of patients and donors may not apply to other populations with different ethnic groups because of the racial variability of SNPs [43]. In addition, the SNPs that were analyzed in this study were selected according to the reports by Pertersdorf et al. [18], which mainly addressed the populations of white (81%) and Hispanic (8%) persons. The SNPs that are unique to the Taiwanese population and are involved in modulating the outcomes of haplo-HSCT may not be unveiled because of the probable biases of SNP selection based on the studies from different countries and donor populations. Since both demography and standards of treatment are changed over time, slow accrual of patients is another limitation of this study. Whether the outcome-associated SNPs as reported in this study can serve as a donor selection criterion to improve the successful rate of haplo-HSCT may still require more cases and functional studies to verify.

## 5. Conclusions

In conclusion, a total of eight SNPs of the genes under study are associated with the post-haplo-HSCT outcomes for patient with AML and ALL. Despite the preliminary nature of the study, our findings underscore the potential that genetic assessment could help in the choice of more suited haploidentical donors. In the future, the genetic information could be privileged risk factors to be considered for selection of appropriate donors for haplo-HSCT and advancing the therapeutic potential of haploidentical transplantation in clinical practice, provided that enough evidence is available. Alternatively, when significant research and development efforts guarantee the safety, efficacy, and ethical considerations associated with the application of CRISPR/Cas9 technology, it is conceivable that CRISPR/Cas9 technology could be utilized to edit the genes of transplants before transplantation into patients, potentially improving their prognosis [44]. The nature per se of this study is an association study of genetic elements and the outcomes after haplo-HSCT. The biological basis and the underlying genetic mechanisms of our findings are warranted to be elucidated further.

## Figures and Tables

**Table 1:** The 41 candidate SNPs of HLA-related genes.

Gene	Source	Candidate SNPs under Analysis						
HCP5	Donor	rs9281491	rs2244546	rs4713466	rs2523676	rs2523675	rs2518028	rs1414315
NOTCH4	Donor	rs111394117	rs429853	rs394657	rs2256594	rs444472	rs61365987	
HLA-DOA	Recipient	rs9276982	rs71565361	rs79327197	rs151190962	rs9282369		
LTA	Recipient	rs2009658	rs736160	rs915654				
HSPA1L	Recipient	rs34324979	rs2075800	rs2227956				
BAG6	Mismatch	rs3130048	rs2844464	rs2242656				
RING1	Mismatch	rs107822	rs213210					
TRIM27	Mismatch	rs209132	rs209131	rs209130	rs1536215	rs139791445		
HLA-DOB	Mismatch	rs11244	rs2070120	rs56150445	rs41258084	rs17220087	rs2071479	rs17213693

**Table 2:** The 34 candidate SNPs of the genes located in the non-HLA region.

Gene	Genomic Region	Candidate SNP under Analysis						
CTLA4	Promoter	rs11571315	rs733618	rs4553808	rs11571316	rs62182595	rs573554201	rs16840252
		rs945677329	rs5742909					
	Exon 1	rs231775						
	Exon 4	rs56102377	rs56217811	rs55696217				
	3′-UTR	rs231721	rs778932058	rs3087243	rs11571319			
TNFSF4	Promoter	rs1234314	rs45454293	rs181758110				
CD28	Promoter	rs1879877	rs3181096	rs3181097	rs3181098	rs28718975	rs28688913	rs28541784
		rs201801072	rs200353921					
PDCD1	Promoter	rs5839828	rs36084323					
	Intron 4	rs41386349	rs6705653					
	Exon 5	rs2227982						

**Table 3:** The primer sequences and the PCR program for amplifying DNA fragments covering the candidate SNPs.

Gene	Primer Sequences ^a^	PCR Program
CD28	F: 5′-GGGTGGTAAGAATGTGGATGAATC-3′ R: 5′-CAAGGCATCCTGACTGCAGCA-3′	1 cycle of 95 °C for 4 min, 30 cycles of 94 °C for 30 s, 58 °C for 30 s, and 72 °C for 45 s, and 1 cycle of 72 °C for 10 min
HCP5	F: 5′-GGGCAACTAAGTCAGGTCTAG-3′ R: 5′-TCTGCAGGTCTCATGGAGAG-3′	
HLA-DOA	F: 5′-CAACAACGTAAAGCTAACGTCTGTG-3′ R: 5′-GCACCACTCTTAGTTATGTATAGG-3′	
HLA-DOB	F: 5′-TCTTCTGAAGACTGTGGAGACTGC-3′ R: 5′-TCCCATAGGAGCTCAGTCTGAAT-3′	
HSPA1L	F: 5′-TCCCCTTCAAGGTACATTCACAGCC-3′ R: 5′-TGATCCAGGTGTATGAGGGCGAGAG-3′	
LTA	F: 5′-AGCATAAAAGGCAAAGGGGCAG-3′ R: 5′-TTAGGTATGAGGTGGACACCTC-3′	
NOTCH4	F: 5′-GATTGTCTGTTGGGTGACCTGAG-3′ R: 5′-TGAGGCTGATCACAATGAGTGCCTCTC-3′	
RING1	F: 5′-TAATCGACTCTGGCGCCCACAT-3′ R: 5′-AACAACCTTAGCCTCGGTTCCCTT-3′	
TRIM27	F: 5′-AGTCGGGATTACAGAAATGCACC-3′ R: 5′-GCAGGACATTTGAAGGTAACC-3′	
BAG6	F: 5′-ATTCATTCAGGGGCACAAGGGG-3′ R: 5′-GCGGAGGTTGAAGAGAATAGAAGC-3′	1 cycle of 95 °C for 3 min, 30 cycles of 95 °C for 30 s, 60 °C for 30 s, and 72 °C for 60 s, and 1 cycle of 72 °C for 10 min
TNFSF4	F: 5′-GGCTTGGAGTCTATGATATTGTGCC-3′ R: 5′-GAAGGGCGTTTAACCACACTTTACG-3′	
CTLA4-1	F: 5′-GGCAACAGAGACCCCACCGTT-3′ R: 5′-GAGGACCTTCCTTAAATCTGGAGAG-3′	1 cycle of 95 °C for 10 min, 35 cycles of 94 °C for 30 s, 65.5 °C for 30 s, and 72 °C for 60 s, and 1 cycle of 72 °C for 3 min
CTLA4-2	F: 5′-CTCTCCAGATTTAAGGAAGGTCCT C-3′ R: 5′-GGAATACAGAGC CAGCCAAGC C-3′	
CTLA4-3	F: 5′-CTAGGGACCCAATATGTGTTG-3′ R: 5′-AGAAACATCCCAGCT CTGTC-3′	1 cycle of 95 °C for 10 min, 35 cycles of 94 °C for 30 s, 59 °C for 30 s, and 72 °C for 60 s, and 1 cycle of 72 °C for 3 min
CTLA4-4	F: 5′-GCTTGGAAACTGGATGAGGTCATAGC-3′ R: 5′-AGAGGAAGAGACACAGACAGAGTTGC-3′	
PDCD1-1	F: 5′-ACCCACACAGCCTCACATCTCT-3′ R: 5′-AAACTGAGGGTGGAAGGTCCCT-3′	1 cycle at 94 °C for 4 min, 30 cycles at 94 °C for 30 s, 55 °C for 30 s, and 72 °C for 60 s, and 1 cycle at 72 °C for 7 min
PDCD1-2	F: 5′-TGGTGACCCCAAGTGTGTTTCTC-3′ R: 5′-GAGGAATTT TTCACCGGAGGGC-3′	1 cycle at 94 °C for 4 min, 30 cycles at 95 °C for 30 s, 61 °C for 30 s, and 72 °C for 120 s, and 1 cycle at 72 °C for 10 min

^a^ F: forward primer; R: reverse primer.

**Table 4:** The clinical characteristics of patients.

Patient Characteristics	N (%)
Number of patients	24
Gender (female/male)	
Recipient	12 (50.0):12 (50.0)
Donor	9 (37.5):15 (62.5)
Age, median years (range)	43.9 (8 m–67 y)
Diseases	
AML	17 (70.8)
ALL	7 (29.2)
Type of donor	
Parents	4 (16.7)
Siblings	12 (50.0)
Offspring	6 (25.0)
Unrelated	2 (8.3)
Sex pairing (donor → recipient)	
M → F	8 (33.3)
M → M	7 (29.2)
F → F	4 (16.7)
F → M	5 (20.8)
Graft source	
Peripheral blood	24 (100.0)
Conditioning regimen	
Myeloablative	10 (42)
Reduced intensity	14 (58)
GVHD prophylaxis	
Post-transplant cyclophosphamide	24 (100.0)
CMV serostatus	
R-/D-	1 (4.1)
R-/D+	0 (0)
R+/D-	4 (16.7)
R+/D+	15 (62.5)
Unknown	4 (16.7)
Median follow-up among survivors, months (range)	29 (6–99)

AML: acute myeloid leukemia; ALL: acute lymphoblastic leukemia. M: male; F: female; R: recipient; D: donor.

**Table 5:** Clinical outcomes of patient receiving haplo-HSCT.

Clinical Outcomes	N (%)
Survival status	
Alive	13 (54.2)
Death	11 (45.8)
Relapse status	
Relapse	16 (66.7)
No relapse	8 (33.3)
aGVHD	14 (58.3)
GVHD I–II	8 (33.3)
GVHD III–IV	6 (25.0)
cGVHD	4 (16.7)
No GVHD	6 (25.0)
Other complications	
Hemorrhagic cystitis (BK virus related)	6 (25.0)
Myelitis	1 (4.1)
Secondary graft failure	2 (8.2)
Septic shock and congestive heart failure	1 (4.1)
None	14 (58.3)

**Table 6:** The SNPs associated with the outcomes of haplo-HSCT in recipient genotype analysis.

SNP	Genes	Outcome	No. of Patients (%)			Model	*p*-Value
rs79327197	HLA-DOA	Survival	AA	AG	GG	Heterozygous	0.031
		Alive	13 (65.0)	0 (0.0)	0 (0.0)		
		Death	7 (35.0)	4 (100.0)	0 (0.0)		
rs107822	RING1	Survival	TT	CT	CC	Dominant	0.036
		Alive	10 (71.4)	2 (25.0)	0 (0.0)		
		Death	4 (28.6)	6 (75.0)	1 (100.0)		

**Table 7:** The SNPs associated with the outcomes of haplo-HSCT in donor genotype analysis.

SNP	Genes	Outcome	No of Patients (%)			Model	*p*-Value
rs5742909	CTLA4	Survival	CC	CT	TT	Heterozygous	0.041
		Alive	8 (42.1)	5 (100.0)	0 (0.0)		
		Death	11 (57.9)	0 (0.0)	0 (0.0)		
rs1234314	TNFSF4	Survival	CC	CG	GG	Dominant	0.033
		Alive	7 (87.5)	4 (36.4)	2 (40.0)		
		Death	1 (12.5)	7 (63.6)	3 (60.0)		
rs107822	RING1	Relapse	TT	CT	CC	Additive	0.047
		Yes	9 (75.0)	2 (22.2)	1 (33.3)	Dominant	0.014
		No	3 (25.0)	7 (77.8)	2 (66.7)	Heterozygous	0.030
rs36084323	PDCD1	Relapse	CC	CT	TT	Additive	0.042
		Yes	1 (14.3)	6 (54.5)	5 (83.3)		
		No	6 (85.7)	5 (45.5)	1 (16.7)		
rs5839828	PDCD1	Relapse	del/del	del/G	GG	Additive	0.014
		Yes	6 (85.7)	6 (50.0)	0 (0.0)	Recessive	0.037
		No	1 (14.3)	6 (50.0)	5 (100.0)	Homozygous	0.015
rs2523676	HCP5	GVHD	CC	CT	TT	Additive	0.026
		Yes	4 (40.0)	10 (83.3)	0 (0.0)		
		No	6 (60.0)	2 (16.7)	2 (100.0)		
rs213210	RING1	GVHD I-II	GG	AG	AA	Additive	0.045
		Yes	2 (22.2)	6 (60.0)	0 (0.0)		
		No	7 (77.8)	4 (40.0)	5 (100.0)		
rs213210	RING1	GVHD III-IV	GG	AG	AA	Additive	0.031
		Yes	3 (33.3)	0 (0.0)	3 (60.0)		
		No	6 (66.7)	10 (100.0)	2 (40.0)		
rs107822	RING1	cGVHD	TT	CT	CC	Additive	0.018
		Yes	0 (0.0)	4 (44.4)	0 (0.0)	Heterozygous	0.021
		No	12 (100.0)	5 (55.6)	3 (100.0)		

**Table 8:** The mismatched status of donor and recipient genotypes were associated with the outcomes of haplo-HSCT.

SNP	Gene	Outcome	Mismatched Frequency (%)		*p*-Value
rs107822	RING1	Relapse	Matched	Mismatched	0.006
		Yes	9 (81.8)	3 (25)	
		No	2 (18.2)	9 (75)	

## Data Availability

The datasets used and/or analyzed during the current study are available from the corresponding author on reasonable request.

## Supplementary Materials

The following supporting information can be downloaded at: https://www.mdpi.com/article/10.3390/jcm13164681/s1, Table S1: The detailed information of the participants. Table S2: The association analysis between SNPs and survival of haplo-HSCT; Table S3: The association analysis between SNPs and relapse of haplo-HSCT; Table S4: The association analysis between SNPs and GVHD or not of haplo-HSCT; Table S5: The association analysis between SNPs and GVHD I–II of haplo-HSCT; Table S6: The association analysis between SNPs and GVHD III–IV of haplo-HSCT; Table S7: The association analysis between SNPs and cGVHD of haplo-HSCT; Figure S1: Kaplan–Meier survival curve analysis for patients with the indicated SNPs receiving haplo-HSCT. The SNPs were associated with the survival of patients in the recipient genotype analysis; Figure S2: Kaplan–Meier survival curve analysis for patients with the indicated SNPs receiving haplo-HSCT. The SNPs were associated with the indicated end-point in the donor genotype analysis; Figure S3: Kaplan–Meier survival curve analysis for patients with the indicated SNPs receiving haplo-HSCT. The SNPs were associated with the relapse of patients in the analysis of mismatched status of donor and recipient genotypes.
