# Supplementary material for: Preliminary Data on SNP of Transplantation-Related Genes after Haploidentical Stem Cell Transplantation
Source: J Clin Med. 2024 Aug 9;13(16):4681. doi: 10.3390/jcm13164681 (PMC11354871; doi:10.3390/jcm13164681)
Supplement: Supplementary file 1 [file jcm-13-04681-s001.zip › Supplementary Figures1-3-20240718.pdf]

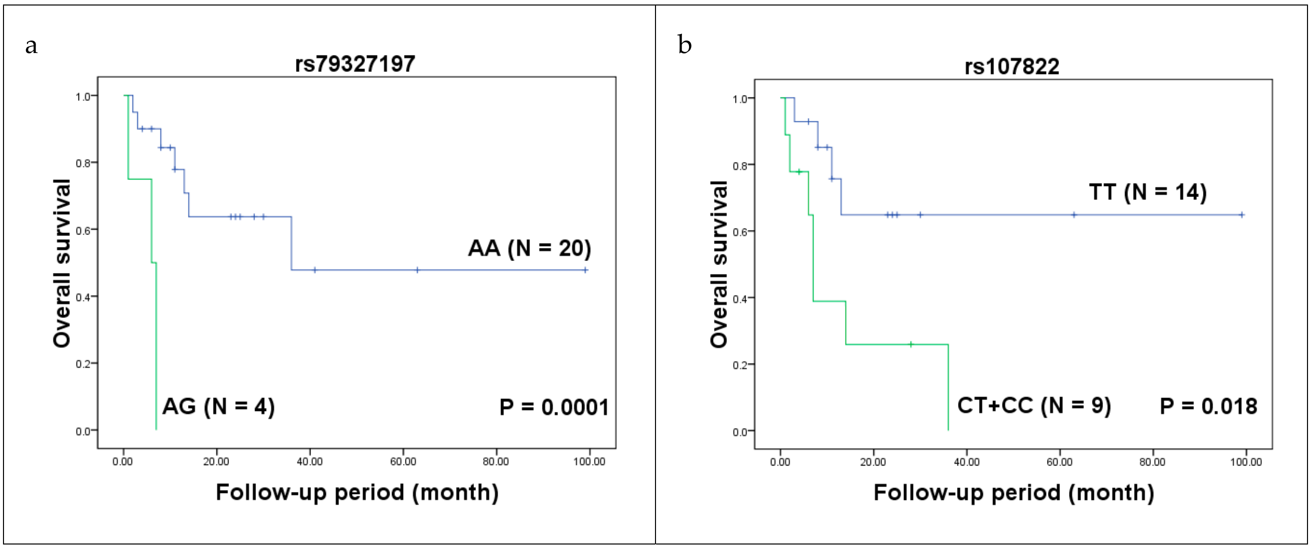

Figure S1. (a-b) Kaplan–Meier survival curve analysis for patients with the indicated SNPs receiving haplo-HSCT. The SNPs were associated with the survival of patients in the recipient genotype analysis.

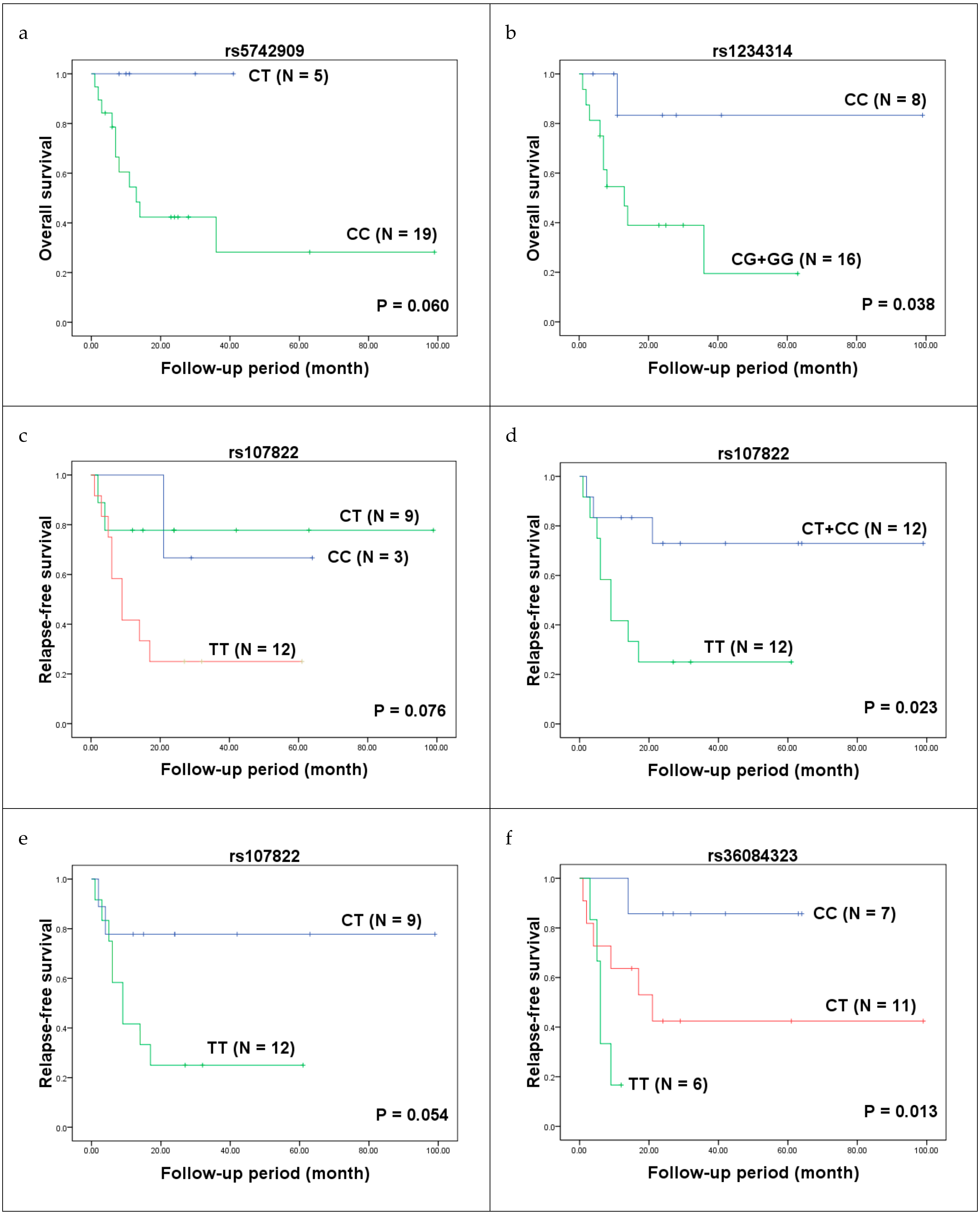
a
rs5742909
CT (N = 5)
CC (N = 19)
P = 0.060
Overall survival
Follow-up period (month)
b
rs1234314
CC (N = 8)
CG+GG (N = 16)
P = 0.038
Overall survival
Follow-up period (month)
c
rs107822
CT (N = 9)
CC (N = 3)
TT (N = 12)
P = 0.076
Relapse-free survival
Follow-up period (month)
d
rs107822
CT+CC (N = 12)
TT (N = 12)
P = 0.023
Relapse-free survival
Follow-up period (month)
e
rs107822
CT (N = 9)
TT (N = 12)
P = 0.054
Relapse-free survival
Follow-up period (month)
f
rs36084323
CC (N = 7)
CT (N = 11)
TT (N = 6)
P = 0.013
Relapse-free survival
Follow-up period (month)

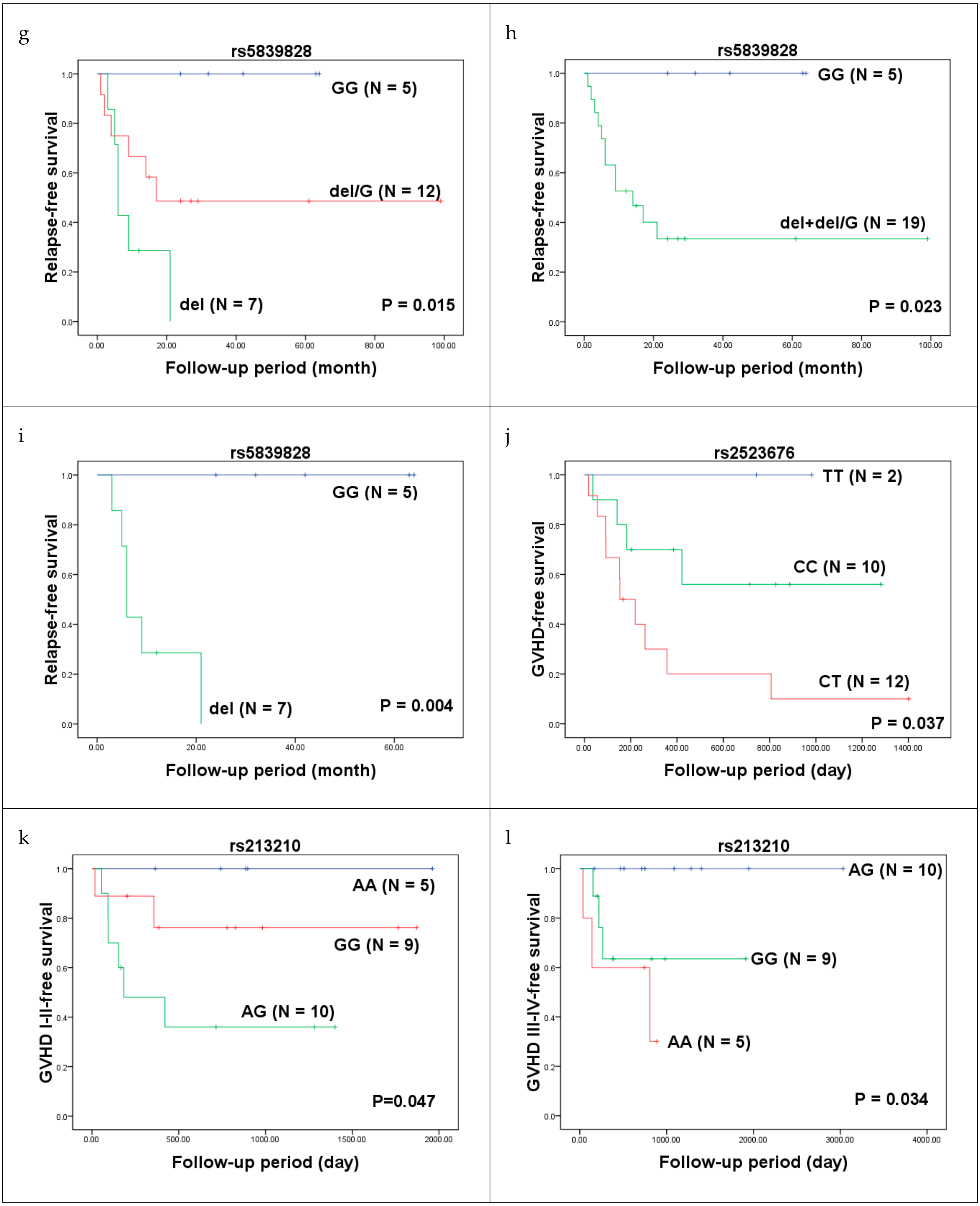
g
rs5839828
Relapse-free survival
GG (N = 5)
del/G (N = 12)
del (N = 7)
P = 0.015
Follow-up period (month)
h
rs5839828
Relapse-free survival
GG (N = 5)
del+del/G (N = 19)
P = 0.023
Follow-up period (month)
i
rs5839828
Relapse-free survival
GG (N = 5)
del (N = 7)
P = 0.004
Follow-up period (month)
j
rs2523676
GVHD-free survival
TT (N = 2)
CC (N = 10)
CT (N = 12)
P = 0.037
Follow-up period (day)
k
rs213210
GVHD I-II-free survival
AA (N = 5)
GG (N = 9)
AG (N = 10)
P=0.047
Follow-up period (day)
l
rs213210
GVHD III-IV-free survival
AG (N = 10)
GG (N = 9)
AA (N = 5)
P = 0.034
Follow-up period (day)

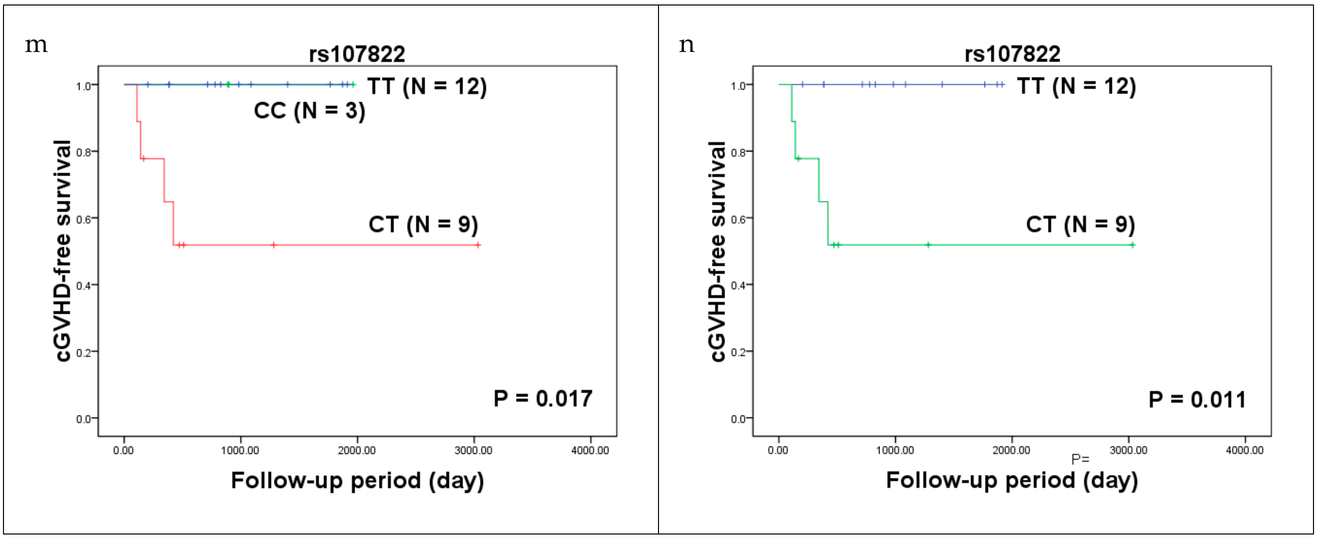

Figure S2. (a-n) Kaplan–Meier survival curve analysis for patients with the indicated SNPs receiving haplo-HSCT. The SNPs were associated with the indicated end-point in the donor genotype analysis.

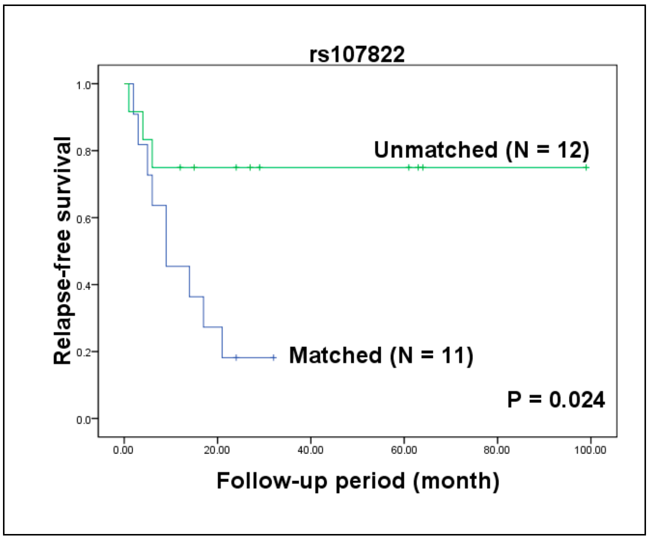

Figure S3. Kaplan–Meier survival curve analysis for patients with the indicated SNPs receiving haplo-HSCT. The SNPs were associated with the relapse of patients in the analysis of mismatched status of donor and recipient genotypes.
