# Supplementary material for: Preliminary Data on SNP of Transplantation-Related Genes after Haploidentical Stem Cell Transplantation
Source: J Clin Med. 2024 Aug 9;13(16):4681. doi: 10.3390/jcm13164681 (PMC11354871; doi:10.3390/jcm13164681)
Supplement: Supplementary file 1 [file jcm-13-04681-s001.zip › Supplementary Table 1_20240718.pdf]

Table S1. The detailed information of the recipient characteristics and outcomes status.

Cases	Gender	Age		CMV status		Conditioning regimen	Donor sources	Survival	aGVHD	cGVHD	Relapse
		Recipient	Donor	Recipient	Donor						
**1**	Female	45	20	positive	positive	RIC	son	alive	NO	NO	YES
**2**	Male	18	45	positive		MAC	father	alive	1-2	NO	No
**3**	Female	52	21	positive	negative	RIC	son	alive	1-2	YES	No
**4**	Female	8m	28	positive	positive	MAC	father	death	3-4	NO	No
**5**	Female	26	31	positive		RIC	sibling	death	1-2	NO	YES
**6**	Male	23	51	positive		MAC	mother	death	3-4	NO	No
**7**	Female	60	21	positive		RIC	daughter	death	3-4	NO	YES
**8**	Male	39	41	positive	positive	MAC	sibling	death	NO	NO	YES
**9**	Male	41	45	positive	positive	MAC	unrelated	alive	NO	NO	No
**10**	Male	25	54	positive	positive	MAC	father	death	1-2	NO	YES
**11**	Male	46	26	positive	positive	RIC	daughter	alive	3-4	NO	YES
**12**	Male	39	33	positive	negative	MAC	sibling	death	3-4	NO	YES
**13**	Male	46	41	positive	positive	MAC	sibling	alive	NO	NO	No
**14**	Female	39	28	positive	positive	MAC	unrelated	death	NO	NO	No
**15**	Female	58	34	positive	positive	RIC	daughter	death	1-2	YES	No
**16**	Male	64	33	positive	negative	RIC	daughter	alive	NO	YES	No
**17**	Male	66	38	positive	positive	RIC	son	alive	NO	NO	YES
**18**	Female	49	20	positive	negative	RIC	daughter	alive	1-2	NO	YES
**19**	Female	55	29	positive	positive	RIC	son	death	1-2	NO	No
**20**	Male	50	52	positive	positive	MAC	sibling	alive	NO	NO	No
**21**	Female	53	25	positive	positive	RIC	son	death	1-2	NO	YES
**22**	Female	64	41	positive	positive	RIC	daughter	alive	3-4	YES	No
**23**	Male	27	30	negative	negative	RIC	sibling	alive	NO	NO	YES
**24**	Female	67	45	positive	positive	RIC	son	alive	NO	NO	YES
