# Supplementary material for: Preliminary Data on SNP of Transplantation-Related Genes after Haploidentical Stem Cell Transplantation
Source: J Clin Med. 2024 Aug 9;13(16):4681. doi: 10.3390/jcm13164681 (PMC11354871; doi:10.3390/jcm13164681)
Supplement: Supplementary file 1 [file jcm-13-04681-s001.zip › Supplementary Table 2_20240718.pdf]

Table S2. Association analysis for SNPs and the survival status post-haplo-HSCT.

Source of DNA[a]	SNP	Gene	Outcome /status	Number of patients			Model	*p*-value
**P**	rs9276982	HLA-DOA	survival	GG	AG	AA	Additive	0.082
			Yes	11	2	0	Dominant	0.082
				68.8%	25.0%	0.0%	Recessive	NA
			No	5	6	0	Homozygous	NA
				31.3%	75.0%	0.0%	Heterozygous	0.082
**P**	rs71565361	HLA-DOA	survival	CC	CA	AA	Additive	0.458
			Yes	13	0	0	Dominant	0.458
				56.5%	0.0%	0.0%	Recessive	NA
			No	10	1	0	Homozygous	NA
				43.5%	100.0%	0.0%	Heterozygous	0.458
**P**	rs79327197	HLA-DOA	survival	AA	AG	GG	Additive	**0.031**
			Yes	13	0	0	Dominant	**0.031**
				65.0%	0.0%	0.0%	Recessive	NA
			No	7	4	0	Homozygous	NA
				35.0%	100.0%	0.0%	Heterozygous	**0.031**
**P**	rs151190962	HLA-DOA	survival	AA	delA	del	Additive	0.458
			Yes	13	0	0	Dominant	0.458
				56.5%	0.0%	0.0%	Recessive	NA
			No	10	1	0	Homozygous	NA
				43.5%	100.0%	0.0%	Heterozygous	0.458
**P**	rs9282369	HLA-DOA	survival	ins	insT	TT	Additive	0.162
			Yes	5	3	5	Dominant	1.000
				55.6%	33.3%	83.3%	Recessive	0.166
			No	4	6	1	Homozygous	0.580
				44.4%	66.7%	16.7%	Heterozygous	0.637
**P**	rs2009658	LTA	survival	CC	CG	GG	Additive	0.679
			Yes	8	5	0	Dominant	0.679
				50.0%	62.5%	0.0%	Recessive	NA
			No	8	3	0	Homozygous	NA
				50.0%	37.5%	0.0%	Heterozygous	0.679
**P**	rs111250247	LTA	survival	TT	TA	AA	Additive	NA
			Yes	13	0	0	Dominant	NA
				54.2%	0.0%	0.0%	Recessive	NA
			No	11	0	0	Homozygous	NA
				45.8%	0.0%	0.0%	Heterozygous	NA
**P**	rs915654	LTA	survival	AA	TA	TT	Additive	0.831
			Yes	3	8	2	Dominant	1.000
				60.0%	50.0%	66.7%	Recessive	1.000
			No	2	8	1	Homozygous	1.000
				40.0%	50.0%	33.3%	Heterozygous	1.000
**P**	rs34324979	HSPA1L	survival	GG	Gins	ins	Additive	0.649
			Yes	9	4	0	Dominant	0.649
				50.0%	66.7%	0.0%	Recessive	NA
			No	9	2	0	Homozygous	NA
				50.0%	33.3%	0.0%	Heterozygous	0.649
**P**	rs2075800	HSPA1L	survival	CC	CT	TT	Additive	0.777
			Yes	4	7	2	Dominant	0.649

				66.7%	50.0%	50.0%	Recessive	1.000
			No	2	7	2	Homozygous	1.000
				33.3%	50.0%	50.0%	Heterozygous	0.642
**P**	rs2227956	HSPA1L	survival	AA	GA	GG	Additive	1.000
			Yes	11	2	0	Dominant	1.000
				55.0%	50.0%	0.0%	Recessive	NA
			No	9	2	0	Homozygous	NA
				45.0%	50.0%	0.0%	Heterozygous	1.000
**P**	rs107822	RING1	survival	TT	CT	CC	Additive	0.063
			Yes	10	2	0	Dominant	**0.036**
				71.4%	25.0%	0.0%	Recessive	0.478
			No	4	6	1	Homozygous	0.333
				28.6%	75.0%	100.0%	Heterozygous	0.074
**P**	rs213210	RING1	survival	GG	AG	AA	Additive	0.143
			Yes	9	3	0	Dominant	0.100
				69.2%	33.3%	0.0%	Recessive	0.478
			No	4	6	1	Homozygous	0.357
				30.8%	66.7%	100.0%	Heterozygous	0.192
**P**	rs209132	TRIM27	survival	GG	AG	AA	Additive	0.504
			Yes	8	3	2	Dominant	0.431
				61.5%	37.5%	66.7%	Recessive	1.000
			No	5	5	1	Homozygous	1.000
				38.5%	62.5%	33.3%	Heterozygous	0.387
**P**	rs209131	TRIM27	survival	GG	AG	AA	Additive	0.730
			Yes	5	5	3	Dominant	0.679
				62.5%	45.5%	60.0%	Recessive	1.000
			No	3	6	2	Homozygous	1.000
				37.5%	54.5%	40.0%	Heterozygous	0.650
**P**	rs209130	TRIM27	survival	TT	CT	CC	Additive	0.169
			Yes	8	3	2	Dominant	0.431
				61.5%	33.3%	100.0%	Recessive	0.482
			No	5	6	0	Homozygous	0.524
				38.5%	66.7%	0.0%	Heterozygous	0.387
**P**	rs1536215	TRIM27	survival	CC	CG	GG	Additive	0.659
			Yes	10	3	0	Dominant	0.659
				58.8%	42.9%	0.0%	Recessive	NA
			No	7	4	0	Homozygous	NA
				41.2%	57.1%	0.0%	Heterozygous	0.659
**P**	rs139791445	TRIM27	survival	CC	CG	GG	Additive	NA
			Yes	11	0	0	Dominant	NA
				45.8%	0.0%	0.0%	Recessive	NA
			No	13	0	0	Homozygous	NA
				54.2%	0.0%	0.0%	Heterozygous	NA
**P**	rs11244	HLA-DOB	survival	GG	AG	AA	Additive	0.410
			Yes	7	4	2	Dominant	0.669
				50.0%	57.1%	100.0%	Recessive	0.486
			No	7	3	0	Homozygous	0.475
				50.0%	42.9%	0.0%	Heterozygous	1.000
**P**	rs2070120	HLA-DOB	survival	GG	AG	AA	Additive	1.000

			Yes	11	2	0	Dominant	1.000
				55.0%	66.7%	0.0%	Recessive	NA
			No	9	1	0	Homozygous	NA
				45.0%	33.3%	0.0%	Heterozygous	1.000
**P**	rs56150445	HLA-DOB	survival	GG	CG	CC	Additive	NA
			Yes	13	0	0	Dominant	NA
				56.5%	0.0%	0.0%	Recessive	NA
			No	10	0	0	Homozygous	NA
				43.5%	0.0%	0.0%	Heterozygous	NA
**P**	rs41258084	HLA-DOB	survival	CC	CT	TT	Additive	0.486
			Yes	11	2	0	Dominant	0.486
				52.4%	100.0%	0.0%	Recessive	NA
			No	10	0	0	Homozygous	NA
				47.6%	0.0%	0.0%	Heterozygous	0.486
**P**	rs17220087	HLA-DOB	survival	CC	AC	AA	Additive	1.000
			Yes	11	2	0	Dominant	1.000
				55.0%	66.7%	0.0%	Recessive	NA
			No	9	1	0	Homozygous	NA
				45.0%	33.3%	0.0%	Heterozygous	1.000
**P**	rs2071479	HLA-DOB	survival	CC	CT	TT	Additive	1.000
			Yes	12	1	0	Dominant	1.000
				54.5%	100.0%	0.0%	Recessive	NA
			No	10	0	0	Homozygous	NA
				45.5%	0.0%	0.0%	Heterozygous	1.000
**P**	rs17213693	HLA-DOB	survival	GG	CG	CC	Additive	0.604
			Yes	10	3	0	Dominant	0.604
				52.6%	75.0%	0.0%	Recessive	NA
			No	9	1	0	Homozygous	NA
				47.4%	25.0%	0.0%	Heterozygous	0.604
**P**	rs3130048	BAG6	survival	TT	CT	CC	Additive	0.109
			Yes	10	3	0	Dominant	0.095
				71.4%	33.3%	0.0%	Recessive	0.458
			No	4	6	1	Homozygous	0.333
				28.6%	66.7%	100.0%	Heterozygous	0.102
**P**	rs2844464	BAG6	survival	GG	AG	AA	Additive	0.237
			Yes	8	4	1	Dominant	0.166
				44.4%	80.0%	100.0%	Recessive	1.000
			No	10	1	0	Homozygous	0.474
				55.6%	20.0%	0.0%	Heterozygous	0.317
**P**	rs2242656	BAG6	survival	TT	CT	CC	Additive	0.327
			Yes	9	4	0	Dominant	0.327
				47.4%	80.0%	0.0%	Recessive	NA
			No	10	1	0	Homozygous	NA
				52.6%	20.0%	0.0%	Heterozygous	0.327
**D**	rs61365987	NOTCH4	survival	CC	CT	TT	Additive	0.082
			Yes	13	0	0	Dominant	0.082
				61.9%	0.0%	0.0%	Recessive	NA
			No	8	3	0	Homozygous	NA
				38.1%	100.0%	0.0%	Heterozygous	0.082

**D**	rs444472	NOTCH4	survival	GG	GA	AA	Additive	0.303
			Yes	7	5	1	Dominant	0.211
				43.8%	71.4%	100.0%	Recessive	1.000
			No	9	2	0	Homozygous	0.471
				56.3%	28.6%	0.0%	Heterozygous	0.371
**D**	rs2256594	NOTCH4	survival	AA	AG	GG	Additive	0.714
			Yes	3	8	2	Dominant	0.659
				42.9%	61.5%	50.0%	Recessive	1.000
			No	4	5	2	Homozygous	1.000
				57.1%	38.5%	50.0%	Heterozygous	0.642
**D**	rs394657	NOTCH4	survival	AA	AG	GG	Additive	0.303
			Yes	7	5	1	Dominant	0.211
				43.8%	71.4%	100.0%	Recessive	1.000
			No	9	2	0	Homozygous	0.471
				56.3%	28.6%	0.0%	Heterozygous	0.371
**D**	rs429853	NOTCH4	survival	TT	TC	CC	Additive	0.386
			Yes	8	5	0	Dominant	0.386
				47.1%	71.4%	0.0%	Recessive	NA
			No	9	2	0	Homozygous	NA
				52.9%	28.6%	0.0%	Heterozygous	0.386
**D**	rs111394117	NOTCH4	survival	GG	GA	AA	Additive	0.082
			Yes	13	0	0	Dominant	0.082
				61.9%	0.0%	0.0%	Recessive	NA
			No	8	3	0	Homozygous	NA
				38.1%	100.0%	0.0%	Heterozygous	0.082
**D**	rs9281491	HCP5	survival	ins	insA	AA	Additive	0.456
			Yes	8	4	1	Dominant	0.386
				47.1%	66.7%	100.0%	Recessive	1.000
			No	9	2	0	Homozygous	1.000
				52.9%	33.3%	0.0%	Heterozygous	0.640
**D**	rs2244546	HCP5	survival	CC	CG	GG	Additive	0.390
			Yes	10	3	0	Dominant	0.390
				62.5%	37.5%	0.0%	Recessive	NA
			No	6	5	0	Homozygous	NA
				37.5%	62.5%	0.0%	Heterozygous	0.390
**D**	rs4713466	HCP5	survival	CC	CT	TT	Additive	0.107
			Yes	9	4	0	Dominant	0.107
				69.2%	36.4%	0.0%	Recessive	NA
			No	4	7	0	Homozygous	NA
				30.8%	63.6%	0.0%	Heterozygous	0.107
**D**	rs2523676	HCP5	survival	CC	CT	TT	Additive	0.091
			Yes	7	4	2	Dominant	0.240
				70.0%	33.3%	100.0%	Recessive	0.482
			No	3	8	0	Homozygous	1.000
				30.0%	66.7%	0.0%	Heterozygous	0.087
**D**	rs2523675	HCP5	survival	GG	GA	AA	Additive	0.104
			Yes	1	10	2	Dominant	0.061
				16.7%	66.7%	66.7%	Recessive	1.000
			No	5	5	1	Homozygous	0.226
				83.3%	33.3%	33.3%	Heterozygous	0.063

**D**	rs2518028	HCP5	survival	CC	TC	TT	Additive	1.000
			Yes	10	3	0	Dominant	1.000
				52.6%	60.0%	0.0%	Recessive	NA
			No	9	2	0	Homozygous	NA
				47.4%	40.0%	0.0%	Heterozygous	1.000
**D**	rs141431529	HCP5	survival	GG	GT	TT	Additive	0.300
			Yes	12	1	0	Dominant	0.300
				60.0%	25.0%	0.0%	Recessive	NA
			No	8	3	0	Homozygous	NA
				40.0%	75.0%	0.0%	Heterozygous	0.300
**D**	rs107822	RING1	survival	TT	CT	CC	Additive	0.192
			Yes	5	7	1	Dominant	0.219
				41.7%	77.8%	33.3%	Recessive	0.576
			No	7	2	2	Homozygous	1.000
				58.3%	22.2%	66.7%	Heterozygous	0.184
**D**	rs213210	RING1	survival	GG	AG	AA	Additive	0.760
			Yes	4	6	3	Dominant	0.675
				44.4%	60.0%	60.0%	Recessive	1.000
			No	5	4	2	Homozygous	1.000
				55.6%	40.0%	40.0%	Heterozygous	0.656
**D**	rs209132	TRIM27	survival	GG	AG	AA	Additive	0.688
			Yes	5	7	1	Dominant	0.431
				45.5%	63.6%	50.0%	Recessive	1.000
			No	6	4	1	Homozygous	1.000
				54.5%	36.4%	50.0%	Heterozygous	0.392
**D**	rs209131	TRIM27	survival	GG	AG	AA	Additive	0.657
			Yes	4	6	3	Dominant	1.000
				50.0%	50.0%	75.0%	Recessive	0.596
			No	4	6	1	Homozygous	0.576
				50.0%	50.0%	25.0%	Heterozygous	1.000
**D**	rs209130	TRIM27	survival	TT	CT	CC	Additive	0.368
			Yes	5	7	1	Dominant	0.219
				41.7%	63.6%	100.0%	Recessive	1.000
			No	7	4	0	Homozygous	0.462
				58.3%	36.4%	0.0%	Heterozygous	0.292
**D**	rs1536215	TRIM27	survival	CC	CG	GG	Additive	0.182
			Yes	11	2	0	Dominant	0.182
				64.7%	28.6%	0.0%	Recessive	NA
			No	6	5	0	Homozygous	NA
				35.3%	71.4%	0.0%	Heterozygous	0.182
**D**	rs139791445	TRIM27	survival	CC	CG	GG	Additive	0.458
			Yes	13	0	0	Dominant	0.458
				56.5%	0.0%	0.0%	Recessive	NA
			No	10	1	0	Homozygous	NA
				43.5%	100.0%	0.0%	Heterozygous	0.458
**D**	rs11244	HLA-DOB	survival	GG	AG	AA	Additive	0.272
			Yes	7	4	2	Dominant	0.240
				70.0%	36.4%	66.7%	Recessive	1.000
			No	3	7	1	Homozygous	1.000

				30.0%	63.6%	33.3%	Heterozygous	0.198
**D**	rs2070120	HLA-DOB	survival	GG	AG	AA	Additive	1.000
			Yes	12	1	0	Dominant	1.000
				52.2%	100.0%	0.0%	Recessive	NA
			No	11	0	0	Homozygous	NA
				47.8%	0.0%	0.0%	Heterozygous	1.000
**D**	rs56150445	HLA-DOB	survival	GG	CG	CC	Additive	NA
			Yes	13	0	0	Dominant	NA
				54.2%	0.0%	0.0%	Recessive	NA
			No	11	0	0	Homozygous	NA
				45.8%	0.0%	0.0%	Heterozygous	NA
**D**	rs41258084	HLA-DOB	survival	CC	CT	TT	Additive	0.482
			Yes	11	2	0	Dominant	0.482
				50.0%	100.0%	0.0%	Recessive	NA
			No	11	0	0	Homozygous	NA
				50.0%	0.0%	0.0%	Heterozygous	0.482
**D**	rs17220087	HLA-DOB	survival	CC	AC	AA	Additive	1.000
			Yes	12	1	0	Dominant	1.000
				52.2%	100.0%	0.0%	Recessive	NA
			No	11	0	0	Homozygous	NA
				47.8%	0.0%	0.0%	Heterozygous	1.000
**D**	rs2071479	HLA-DOB	survival	CC	CT	TT	Additive	0.458
			Yes	13	0	0	Dominant	0.458
				56.5%	0.0%	0.0%	Recessive	NA
			No	10	1	0	Homozygous	NA
				43.5%	100.0%	0.0%	Heterozygous	0.458
**D**	rs17213693	HLA-DOB	survival	GG	CG	CC	Additive	1.000
			Yes	12	1	0	Dominant	1.000
				52.2%	100.0%	0.0%	Recessive	NA
			No	11	0	0	Homozygous	NA
				47.8%	0.0%	0.0%	Heterozygous	1.000
**D**	rs3130048	BAG6	survival	TT	CT	CC	Additive	0.370
			Yes	9	3	1	Dominant	0.408
				64.3%	50.0%	25.0%	Recessive	0.300
			No	5	3	3	Homozygous	0.275
				35.7%	50.0%	75.0%	Heterozygous	0.642
**D**	rs2844464	BAG6	survival	GG	AG	AA	Additive	1.000
			Yes	10	3	0	Dominant	1.000
				52.6%	60.0%	0.0%	Recessive	NA
			No	9	2	0	Homozygous	NA
				47.4%	40.0%	0.0%	Heterozygous	1.000
**D**	rs2242656	BAG6	survival	TT	CT	CC	Additive	1.000
			Yes	10	3	0	Dominant	1.000
				52.6%	60.0%	0.0%	Recessive	NA
			No	9	2	0	Homozygous	NA
				47.4%	40.0%	0.0%	Heterozygous	1.000
**M**	rs107822	RING1	survival		matched		unmatched	0.827
			Yes		6		6	
					54.5%		50.0%	

			No	5	6	
				45.5%	50.0%	
**M**	rs213210	RING1	survival	matched	unmatched	1.000
			Yes	4	8	
				57.1%	50.0%	
			No	3	8	
				42.9%	50.0%	
**M**	rs209132	TRIM27	survival	matched	unmatched	0.423
			Yes	7	6	
				46.7%	66.7%	
			No	8	3	
				53.3%	33.3%	
**M**	rs209131	TRIM27	survival	matched	unmatched	0.697
			Yes	7	6	
				50.0%	60.0%	
			No	7	4	
				50.0%	40.0%	
**M**	rs209130	TRIM27	survival	matched	unmatched	0.697
			Yes	7	6	
				50.0%	60.0%	
			No	7	4	
				50.0%	40.0%	
**M**	rs1536215	TRIM27	survival	matched	unmatched	0.596
			Yes	10	3	
				50.0%	75.0%	
			No	10	1	
				50.0%	25.0%	
**M**	rs139791445	TRIM27	survival	matched	unmatched	0.458
			Yes	13	0	
				56.5%	0.0%	
			No	10	1	
				43.5%	100.0%	
**M**	rs11244	HLA-DOB	survival	matched	unmatched	0.222
			Yes	9	4	
				69.2%	40.0%	
			No	4	6	
				30.8%	60.0%	
**M**	rs2070120	HLA-DOB	survival	matched	unmatched	0.435
			Yes	13	0	
				59.1%	0.0%	
			No	9	1	
				40.9%	100.0%	
**M**	rs56150445	HLA-DOB	survival	matched	unmatched	NA
			Yes	13	0	
				56.5%	0.0%	
			No	10	0	
				43.5%	0.0%	
**M**	rs41258084	HLA-DOB	survival	matched	unmatched	NA
			Yes	13	0	

				56.5%	0.0%	
			No	10	0	
				43.5%	0.0%	
**M**	rs17220087	HLA-DOB	survival	matched	unmatched	1.000
			Yes	12	1	
				57.1%	50.0%	
			No	9	1	
				42.9%	50.0%	
**M**	rs2071479	HLA-DOB	survival	matched	unmatched	1.000
			Yes	12	1	
				54.5%	100.0%	
			No	10	0	
				45.5%	0.0%	
**M**	rs17213693	HLA-DOB	survival	matched	unmatched	1.000
			Yes	12	1	
				54.5%	100.0%	
			No	10	0	
				45.5%	0.0%	
**M**	rs3130048	BAG6	survival	matched	unmatched	0.107
			Yes	9	4	
				69.2%	36.4%	
			No	4	7	
				30.8%	63.6%	
**M**	rs2844464	BAG6	survival	matched	unmatched	1.000
			Yes	9	4	
				52.9%	57.1%	
			No	8	3	
				47.1%	42.9%	
**M**	rs2242656	BAG6	survival	matched	unmatched	1.000
			Yes	10	3	
				55.6%	50.0%	
			No	8	3	
				44.4%	50.0%	

**Source of DNA[a]**	**SNP**	**Gene**	**Outcome /status**	**Number of patients**			**Model**	***p*-value**
**D**	rs11571315	CTLA4	survival	TT	CT	CC	Additive	0.517
			Yes	6	6	1	Dominant	0.392
				46.2%	60.0%	100.0%	Recessive	1.000
			No	7	4	0	Homozygous	1.000
				53.8%	40.0%	0.0%	Heterozygous	0.680
**D**	rs733618	CTLA4	survival	CC	CT	TT	Additive	0.104
			Yes	2	7	4	Dominant	0.357
				33.3%	50.0%	100.0%	Recessive	0.098
			No	4	7	0	Homozygous	0.357
				66.7%	50.0%	0.0%	Heterozygous	0.642
**D**	rs4553808	CTLA4	survival	AA	AG	GG	Additive	0.223
			Yes	10	3	0	Dominant	0.223
				47.6%	100.0%	0.0%	Recessive	NA
			No	11	0	0	Homozygous	NA
				52.4%	0.0%	0.0%	Heterozygous	0.223

**D**	rs11571316	CTLA4	survival	GG	AG	AA	Additive	1.000
			Yes	8	5	0	Dominant	1.000
				57.1%	50.0%	0.0%	Recessive	NA
			No	6	5	0	Homozygous	NA
				42.9%	50.0%	0.0%	Heterozygous	1.000
**D**	rs62182595	CTLA4	survival	GG	AG	AA	Additive	0.223
			Yes	10	3	0	Dominant	0.223
				47.6%	100.0%	0.0%	Recessive	NA
			No	11	0	0	Homozygous	NA
				52.4%	0.0%	0.0%	Heterozygous	0.223
**D**	rs573554201	CTLA4	survival	AA	AG	GG	Additive	NA
			Yes	13	0	0	Dominant	NA
				54.2%	0.0%	0.0%	Recessive	NA
			No	11	0	0	Homozygous	NA
				45.8%	0.0%	0.0%	Heterozygous	NA
**D**	rs16840252	CTLA4	survival	CC	CT	TT	Additive	0.223
			Yes	10	3	0	Dominant	0.223
				47.6%	100.0%	0.0%	Recessive	NA
			No	11	0	0	Homozygous	NA
				52.4%	0.0%	0.0%	Heterozygous	0.223
**D**	rs945677329	CTLA4	survival	AA	AG	GG	Additive	1.000
			Yes	12	1	0	Dominant	1.000
				52.2%	100.0%	0.0%	Recessive	NA
			No	11	0	0	Homozygous	NA
				47.8%	0.0%	0.0%	Heterozygous	1.000
**D**	rs5742909	CTLA4	survival	CC	CT	TT	Additive	**0.041**
			Yes	8	5	0	Dominant	**0.041**
				42.1%	100.0%	0.0%	Recessive	NA
			No	11	0	0	Homozygous	NA
				57.9%	0.0%	0.0%	Heterozygous	**0.041**
**D**	rs231775	CTLA4	survival	GG	AG	AA	Additive	0.531
			Yes	5	7	1	Dominant	0.431
				45.5%	58.3%	100.0%	Recessive	1.000
			No	6	5	0	Homozygous	1.000
				54.5%	41.7%	0.0%	Heterozygous	0.537
**D**	rs56102377	CTLA4	survival	GG	AG	AA	Additive	1.000
			Yes	12	1	0	Dominant	1.000
				54.5%	50.0%	0.0%	Recessive	NA
			No	10	1	0	Homozygous	NA
				45.5%	50.0%	0.0%	Heterozygous	1.000
**D**	rs56217811	CTLA4	survival	CC	CA	AA	Additive	NA
			Yes	13	0	0	Dominant	NA
				54.2%	0.0%	0.0%	Recessive	NA
			No	11	0	0	Homozygous	NA
				45.8%	0.0%	0.0%	Heterozygous	NA
**D**	rs55696217	CTLA4	survival	GG	GA	AA	Additive	NA
			Yes	13	0	0	Dominant	NA
				54.2%	0.0%	0.0%	Recessive	NA
			No	11	0	0	Homozygous	NA
				45.8%	0.0%	0.0%	Heterozygous	NA

**D**	rs231721	CTLA4	survival	TT	CT	CC	Additive	NA
			Yes	13	0	0	Dominant	NA
				54.2%	0.0%	0.0%	Recessive	NA
			No	11	0	0	Homozygous	NA
				45.8%	0.0%	0.0%	Heterozygous	NA
**D**	rs778932058	CTLA4	survival	AA	AG	GG	Additive	NA
			Yes	13	0	0	Dominant	NA
				54.2%	0.0%	0.0%	Recessive	NA
			No	11	0	0	Homozygous	NA
				45.8%	0.0%	0.0%	Heterozygous	NA
**D**	rs3087243	CTLA4	survival	GG	AG	AA	Additive	1.000
			Yes	7	6	0	Dominant	1.000
				53.8%	54.5%	0.0%	Recessive	NA
			No	6	5	0	Homozygous	NA
				46.2%	45.5%	0.0%	Heterozygous	1.000
**D**	rs11571319	CTLA4	survival	GG	AG	AA	Additive	0.098
			Yes	9	4	0	Dominant	0.098
				45.0%	100.0%	0.0%	Recessive	NA
			No	11	0	0	Homozygous	NA
				55.0%	0.0%	0.0%	Heterozygous	0.098
**D**	rs1234314	TNFSF4	survival	CC	CG	GG	Additive	0.068
			Yes	7	4	2	Dominant	**0.033**
				87.5%	36.4%	40.0%	Recessive	0.630
			No	1	7	3	Homozygous	0.217
				12.5%	63.6%	60.0%	Heterozygous	0.059
**D**	rs45454293	TNFSF4	survival	CC	CT	TT	Additive	0.246
			Yes	10	2	1	Dominant	0.206
				66.7%	28.6%	50.0%	Recessive	1.000
			No	5	5	1	Homozygous	1.000
				33.3%	71.4%	50.0%	Heterozygous	0.172
**D**	rs181758110	TNFSF4	survival	CC	CT	TT	Additive	NA
			Yes	13	0	0	Dominant	NA
				54.2%	0.0%	0.0%	Recessive	NA
			No	11	0	0	Homozygous	NA
				45.8%	0.0%	0.0%	Heterozygous	NA
**D**	rs1879877	CD28	survival	TT	GT	GG	Additive	0.418
			Yes	3	8	1	Dominant	0.400
				37.5%	57.1%	100.0%	Recessive	1.000
			No	5	6	0	Homozygous	0.444
				62.5%	42.9%	0.0%	Heterozygous	0.659
**D**	rs3181096	CD28	survival	CC	CT	TT	Additive	1.000
			Yes	7	5	0	Dominant	1.000
				50.0%	55.6%	0.0%	Recessive	NA
			No	7	4	0	Homozygous	NA
				50.0%	44.4%	0.0%	Heterozygous	1.000
**D**	rs3181097	CD28	survival	AA	AG	GG	Additive	0.163
			Yes	2	7	3	Dominant	0.371
				33.3%	50.0%	100.0%	Recessive	0.217
			No	4	7	0	Homozygous	0.167

				66.7%	50.0%	0.0%	Heterozygous	0.642
**D**	rs3181098	CD28	survival	GG	AG	AA	Additive	1.000
			Yes	7	5	0	Dominant	1.000
				50.0%	55.6%	0.0%	Recessive	NA
			No	7	4	0	Homozygous	NA
				50.0%	44.4%	0.0%	Heterozygous	1.000
**D**	rs28718975	CD28	survival	TT	CT	CC	Additive	0.144
			Yes	7	5	0	Dominant	0.371
				43.8%	83.3%	0.0%	Recessive	0.478
			No	9	1	1	Homozygous	1.000
				56.3%	16.7%	100.0%	Heterozygous	0.162
**D**	rs28688913	CD28	survival	CC	CT	TT	Additive	0.371
			Yes	7	5	0	Dominant	0.371
				43.8%	71.4%	0.0%	Recessive	NA
			No	9	2	0	Homozygous	NA
				56.3%	28.6%	0.0%	Heterozygous	0.371
**D**	rs28541784	CD28	survival	CC	CT	TT	Additive	0.155
			Yes	7	5	0	Dominant	0.155
				41.2%	83.3%	0.0%	Recessive	NA
			No	10	1	0	Homozygous	NA
				58.8%	16.7%	0.0%	Heterozygous	0.155
**D**	rs201801072	CD28	survival	TT	CT	CC	Additive	0.371
			Yes	7	5	0	Dominant	0.371
				43.8%	71.4%	0.0%	Recessive	NA
			No	9	2	0	Homozygous	NA
				56.3%	28.6%	0.0%	Heterozygous	0.371
**D**	rs200353921	CD28	survival	TT	AT	AA	Additive	0.371
			Yes	7	5	0	Dominant	0.371
				43.8%	71.4%	0.0%	Recessive	NA
			No	9	2	0	Homozygous	NA
				56.3%	28.6%	0.0%	Heterozygous	0.371
**D**	rs56228674	CD28	survival	CC	CT	TT	Additive	0.478
			Yes	10	2	0	Dominant	0.478
				47.6%	100.0%	0.0%	Recessive	NA
			No	11	0	0	Homozygous	NA
				52.4%	0.0%	0.0%	Heterozygous	0.478
**D**	rs1290180288	CD28	survival	TT	CT	CC	Additive	NA
			Yes	12	0	0	Dominant	NA
				52.2%	0.0%	0.0%	Recessive	NA
			No	11	0	0	Homozygous	NA
				47.8%	0.0%	0.0%	Heterozygous	NA
**D**	rs3116496	CD28	survival	TT	CT	CC	Additive	0.478
			Yes	10	2	0	Dominant	0.478
				47.6%	100.0%	0.0%	Recessive	NA
			No	11	0	0	Homozygous	NA
				52.4%	0.0%	0.0%	Heterozygous	0.478
**D**	rs375758971	CD28	survival	TT	CT	CC	Additive	NA
			Yes	12	0	0	Dominant	NA
				52.2%	0.0%	0.0%	Recessive	NA

			No	11	0	0	Homozygous	NA
				47.8%	0.0%	0.0%	Heterozygous	NA
**D**	rs36084323	PD1	survival	CC	CT	TT	Additive	0.544
			Yes	5	5	3	Dominant	0.386
				71.4%	45.5%	50.0%	Recessive	1.000
			No	2	6	3	Homozygous	0.386
				28.6%	54.5%	50.0%	Heterozygous	0.367
**D**	rs5839828	PD1	survival	del	delG	GG	Additive	0.409
			Yes	3	6	4	Dominant	0.659
				42.9%	50.0%	80.0%	Recessive	0.327
			No	4	6	1	Homozygous	0.293
				57.1%	50.0%	20.0%	Heterozygous	1.000
**D**	rs11568821	PD1	survival	CC	CA	AA	Additive	NA
			Yes	13	0	0	Dominant	NA
				54.2%	0.0%	0.0%	Recessive	NA
			No	11	0	0	Homozygous	NA
				45.8%	0.0%	0.0%	Heterozygous	NA
**D**	rs41386349	PD1	survival	GG	AG	AA	Additive	0.612
			Yes	8	4	1	Dominant	0.679
				50.0%	57.1%	100.0%	Recessive	1.000
			No	8	3	0	Homozygous	1.000
				50.0%	42.9%	0.0%	Heterozygous	1.000
**D**	rs6705653	PD1	survival	CC	CT	TT	Additive	0.304
			Yes	5	7	1	Dominant	0.219
				41.7%	63.6%	100.0%	Recessive	1.000
			No	7	4	0	Homozygous	0.219
				58.3%	36.4%	0.0%	Heterozygous	0.292
**D**	rs2227982	PD1	survival	GG	AG	AA	Additive	0.530
			Yes	5	5	3	Dominant	0.386
				71.4%	50.0%	42.9%	Recessive	0.659
			No	2	5	4	Homozygous	0.592
				28.6%	50.0%	57.1%	Heterozygous	0.622

[a]P: patient; D: donor; M: patient and donor mismatch status.
