# Supplementary material for: Preliminary Data on SNP of Transplantation-Related Genes after Haploidentical Stem Cell Transplantation
Source: J Clin Med. 2024 Aug 9;13(16):4681. doi: 10.3390/jcm13164681 (PMC11354871; doi:10.3390/jcm13164681)
Supplement: Supplementary file 1 [file jcm-13-04681-s001.zip › Supplementary Table 3_20240718.pdf]

Table S3. Association analysis between SNPs and relapse post-haplo-HSCT.

Source of DNA[a]	SNP	Gene	Outcome /status	Number of patients			Model	*p*-value
**P**	rs9276982	HLA-DOA	relapse	GG	AG	AA	Additive	0.193
			Yes	10	2	0	Dominant	0.193
				62.5%	25.0%	0.0%	Recessive	NA
			No	6	6	0	Homozygous	NA
				37.5%	75.0%	0.0%	Heterozygous	0.193
**P**	rs71565361	HLA-DOA	relapse	CC	CA	AA	Additive	1.000
			Yes	12	0	0	Dominant	1.000
				52.2%	0.0%	0.0%	Recessive	NA
			No	11	1	0	Homozygous	NA
				47.8%	100.0%	0.0%	Heterozygous	1.000
**P**	rs79327197	HLA-DOA	relapse	AA	AG	GG	Additive	0.590
			Yes	11	1	0	Dominant	0.590
				55.0%	25.0%	0.0%	Recessive	NA
			No	9	3	0	Homozygous	NA
				45.0%	75.0%	0.0%	Heterozygous	0.590
**P**	rs151190962	HLA-DOA	relapse	AA	delA	del	Additive	1.000
			Yes	11	1	0	Dominant	1.000
				47.8%	100.0%	0.0%	Recessive	NA
			No	12	0	0	Homozygous	NA
				52.2%	0.0%	0.0%	Heterozygous	1.000
**P**	rs9282369	HLA-DOA	relapse	ins	insT	TT	Additive	0.151
			Yes	3	4	5	Dominant	0.400
				33.3%	44.4%	83.3%	Recessive	0.155
			No	6	5	1	Homozygous	0.119
				66.7%	55.6%	16.7%	Heterozygous	1.000
**P**	rs2009658	LTA	relapse	CC	CG	GG	Additive	1.000
			Yes	8	4	0	Dominant	1.000
				50.0%	50.0%	0.0%	Recessive	NA
			No	8	4	0	Homozygous	NA
				50.0%	50.0%	0.0%	Heterozygous	1.000
**P**	rs111250247	LTA	relapse	TT	TA	AA	Additive	NA
			Yes	12	0	0	Dominant	NA
				50.0%	0.0%	0.0%	Recessive	NA
			No	12	0	0	Homozygous	NA
				50.0%	0.0%	0.0%	Heterozygous	NA
**P**	rs915654	LTA	relapse	AA	TA	TT	Additive	0.766
			Yes	2	8	2	Dominant	1.000
				40.0%	50.0%	66.7%	Recessive	1.000
			No	3	8	1	Homozygous	1.000
				60.0%	50.0%	33.3%	Heterozygous	1.000
**P**	rs34324979	HSPA1L	relapse	GG	Gins	ins	Additive	0.155
			Yes	7	5	0	Dominant	0.155
				38.9%	83.3%	0.0%	Recessive	NA

			No	11	1	0	Homozygous	NA
				61.1%	16.7%	0.0%	Heterozygous	0.155
**P**	rs2075800	HSPA1L	relapse	CC	CT	TT	Additive	0.435
			Yes	4	7	1	Dominant	0.640
				66.7%	50.0%	25.0%	Recessive	0.590
			No	2	7	3	Homozygous	0.524
				33.3%	50.0%	75.0%	Heterozygous	0.642
**P**	rs2227956	HSPA1L	relapse	AA	GA	GG	Additive	0.590
			Yes	11	1	0	Dominant	0.590
				55.0%	25.0%	0.0%	Recessive	NA
			No	9	3	0	Homozygous	NA
				45.0%	75.0%	0.0%	Heterozygous	0.590
**P**	rs107822	RING1	relapse	TT	CT	CC	Additive	0.418
			Yes	8	3	1	Dominant	0.680
				57.1%	37.5%	100.0%	Recessive	1.000
			No	6	5	0	Homozygous	1.000
				42.9%	62.5%	0.0%	Heterozygous	0.659
**P**	rs213210	RING1	relapse	GG	AG	AA	Additive	0.564
			Yes	7	4	1	Dominant	1.000
				53.8%	44.4%	100.0%	Recessive	1.000
			No	6	5	0	Homozygous	1.000
				46.2%	55.6%	0.0%	Heterozygous	1.000
**P**	rs209132	TRIM27	relapse	GG	AG	AA	Additive	0.815
			Yes	7	4	1	Dominant	0.682
				53.8%	50.0%	33.3%	Recessive	1.000
			No	6	4	2	Homozygous	1.000
				46.2%	50.0%	66.7%	Heterozygous	1.000
**P**	rs209131	TRIM27	relapse	GG	AG	AA	Additive	0.303
			Yes	5	6	1	Dominant	0.667
				62.5%	54.5%	20.0%	Recessive	0.317
			No	3	5	4	Homozygous	0.266
				37.5%	45.5%	80.0%	Heterozygous	1.000
**P**	rs209130	TRIM27	relapse	TT	CT	CC	Additive	0.335
			Yes	7	5	0	Dominant	0.682
				53.8%	55.6%	0.0%	Recessive	0.478
			No	6	4	2	Homozygous	0.467
				46.2%	44.4%	100.0%	Heterozygous	1.000
**P**	rs1536215	TRIM27	relapse	CC	CG	GG	Additive	1.000
			Yes	8	4	0	Dominant	1.000
				47.1%	57.1%	0.0%	Recessive	NA
			No	9	3	0	Homozygous	NA
				52.9%	42.9%	0.0%	Heterozygous	1.000
**P**	rs139791445	TRIM27	relapse	CC	CG	GG	Additive	NA
			Yes	12	0	0	Dominant	NA
				50.0%	0.0%	0.0%	Recessive	NA

			No	12	0	0	Homozygous	NA
				50.0%	0.0%	0.0%	Heterozygous	NA
**P**	rs11244	HLA-DOB	relapse	GG	AG	AA	Additive	0.951
			Yes	7	3	1	Dominant	1.000
				50.0%	42.9%	50.0%	Recessive	1.000
			No	7	4	1	Homozygous	1.000
				50.0%	57.1%	50.0%	Heterozygous	1.000
**P**	rs2070120	HLA-DOB	relapse	GG	AG	AA	Additive	0.590
			Yes	9	2	0	Dominant	0.590
				45.0%	66.7%	0.0%	Recessive	NA
			No	11	1	0	Homozygous	NA
				55.0%	33.3%	0.0%	Heterozygous	0.590
**P**	rs56150445	HLA-DOB	relapse	GG	CG	CC	Additive	NA
			Yes	11	0	0	Dominant	NA
				47.8%	0.0%	0.0%	Recessive	NA
			No	12	0	0	Homozygous	NA
				52.2%	0.0%	0.0%	Heterozygous	NA
**P**	rs41258084	HLA-DOB	relapse	CC	CT	TT	Additive	1.000
			Yes	10	1	0	Dominant	1.000
				47.6%	50.0%	0.0%	Recessive	NA
			No	11	1	0	Homozygous	NA
				52.4%	50.0%	0.0%	Heterozygous	1.000
**P**	rs17220087	HLA-DOB	relapse	CC	AC	AA	Additive	0.590
			Yes	9	2	0	Dominant	0.590
				45.0%	66.7%	0.0%	Recessive	NA
			No	11	1	0	Homozygous	NA
				55.0%	33.3%	0.0%	Heterozygous	0.590
**P**	rs2071479	HLA-DOB	relapse	CC	CT	TT	Additive	1.000
			Yes	11	0	0	Dominant	1.000
				50.0%	0.0%	0.0%	Recessive	NA
			No	11	1	0	Homozygous	NA
				50.0%	100.0%	0.0%	Heterozygous	1.000
**P**	rs17213693	HLA-DOB	relapse	GG	CG	CC	Additive	1.000
			Yes	9	2	0	Dominant	1.000
				47.4%	50.0%	0.0%	Recessive	NA
			No	10	2	0	Homozygous	NA
				52.6%	50.0%	0.0%	Heterozygous	1.000
**P**	rs3130048	BAG6	relapse	TT	CT	CC	Additive	0.319
			Yes	8	3	1	Dominant	0.408
				57.1%	33.3%	100.0%	Recessive	1.000
			No	6	6	0	Homozygous	1.000
				42.9%	66.7%	0.0%	Heterozygous	0.400
**P**	rs2844464	BAG6	relapse	GG	AG	AA	Additive	0.491
			Yes	8	3	1	Dominant	0.640
				44.4%	60.0%	100.0%	Recessive	1.000

			No	10	2	0	Homozygous	0.474
				55.6%	40.0%	0.0%	Heterozygous	0.640
**P**	rs2242656	BAG6	relapse	TT	CT	CC	Additive	1.000
			Yes	9	3	0	Dominant	1.000
				47.4%	60.0%	0.0%	Recessive	NA
			No	10	2	0	Homozygous	NA
				52.6%	40.0%	0.0%	Heterozygous	1.000
**D**	rs61365987	NOTCH4	relapse	CC	CT	TT	Additive	1.000
			Yes	10	2	0	Dominant	1.000
				47.6%	66.7%	0.0%	Recessive	NA
			No	11	1	0	Homozygous	NA
				52.4%	33.3%	0.0%	Heterozygous	1.000
**D**	rs444472	NOTCH4	relapse	GG	GA	AA	Additive	0.281
			Yes	7	5	0	Dominant	0.667
				43.8%	71.4%	0.0%	Recessive	1.000
			No	9	2	1	Homozygous	1.000
				56.3%	28.6%	100.0%	Heterozygous	0.371
**D**	rs2256594	NOTCH4	relapse	AA	AG	GG	Additive	0.307
			Yes	5	6	1	Dominant	0.371
				71.4%	46.2%	25.0%	Recessive	0.590
			No	2	7	3	Homozygous	0.242
				28.6%	53.8%	75.0%	Heterozygous	0.374
**D**	rs394657	NOTCH4	relapse	AA	AG	GG	Additive	0.281
			Yes	7	5	0	Dominant	0.667
				43.8%	71.4%	0.0%	Recessive	1.000
			No	9	2	1	Homozygous	1.000
				56.3%	28.6%	100.0%	Heterozygous	0.371
**D**	rs429853	NOTCH4	relapse	TT	TC	CC	Additive	1.000
			Yes	8	4	0	Dominant	1.000
				47.1%	57.1%	0.0%	Recessive	NA
			No	9	3	0	Homozygous	NA
				52.9%	42.9%	0.0%	Heterozygous	1.000
**D**	rs111394117	NOTCH4	relapse	GG	GA	AA	Additive	1.000
			Yes	11	1	0	Dominant	1.000
				52.4%	33.3%	0.0%	Recessive	NA
			No	10	2	0	Homozygous	NA
				47.6%	66.7%	0.0%	Heterozygous	1.000
**D**	rs9281491	HCP5	relapse	ins	insA	AA	Additive	0.422
			Yes	9	2	1	Dominant	1.000
				52.9%	33.3%	100.0%	Recessive	1.000
			No	8	4	0	Homozygous	1.000
				47.1%	66.7%	0.0%	Heterozygous	0.640
**D**	rs2244546	HCP5	relapse	CC	CG	GG	Additive	1.000
			Yes	8	4	0	Dominant	1.000
				50.0%	50.0%	0.0%	Recessive	NA

			No	8	4	0	Homozygous	NA
				50.0%	50.0%	0.0%	Heterozygous	1.000
**D**	rs4713466	HCP5	relapse	CC	CT	TT	Additive	0.682
			Yes	7	5	0	Dominant	0.682
				53.8%	45.5%	0.0%	Recessive	NA
			No	6	6	0	Homozygous	NA
				46.2%	54.5%	0.0%	Heterozygous	0.682
**D**	rs2523676	HCP5	relapse	CC	CT	TT	Additive	0.155
			Yes	4	8	0	Dominant	0.408
				40.0%	66.7%	0.0%	Recessive	0.478
			No	6	4	2	Homozygous	0.515
				60.0%	33.3%	100.0%	Heterozygous	0.391
**D**	rs2523675	HCP5	relapse	GG	GA	AA	Additive	0.097
			Yes	1	10	1	Dominant	0.155
				16.7%	66.7%	33.3%	Recessive	1.000
			No	5	5	2	Homozygous	1.000
				83.3%	33.3%	66.7%	Heterozygous	0.063
**D**	rs2518028	HCP5	relapse	CC	TC	TT	Additive	0.317
			Yes	11	1	0	Dominant	0.317
				57.9%	20.0%	0.0%	Recessive	NA
			No	8	4	0	Homozygous	NA
				42.1%	80.0%	0.0%	Heterozygous	0.317
**D**	rs141431529	HCP5	relapse	GG	GT	TT	Additive	0.590
			Yes	11	1	0	Dominant	0.590
				55.0%	25.0%	0.0%	Recessive	NA
			No	9	3	0	Homozygous	NA
				45.0%	75.0%	0.0%	Heterozygous	0.590
**D**	rs107822	RING1	relapse	TT	CT	CC	Additive	**0.047**
			Yes	9	2	1	Dominant	**0.014**
				75.0%	22.2%	33.3%	Recessive	1.000
			No	3	7	2	Homozygous	0.242
				25.0%	77.8%	66.7%	Heterozygous	**0.030**
**D**	rs213210	RING1	relapse	GG	AG	AA	Additive	0.247
			Yes	6	5	1	Dominant	0.400
				66.7%	50.0%	20.0%	Recessive	0.317
			No	3	5	4	Homozygous	0.266
				33.3%	50.0%	80.0%	Heterozygous	0.65
**D**	rs209132	TRIM27	relapse	GG	AG	AA	Additive	0.913
			Yes	5	6	1	Dominant	0.682
				45.5%	54.5%	50.0%	Recessive	1.000
			No	6	5	1	Homozygous	1.000
				54.5%	45.5%	50.0%	Heterozygous	0.670
**D**	rs209131	TRIM27	relapse	GG	AG	AA	Additive	0.513
			Yes	4	7	1	Dominant	1.000
				50.0%	58.3%	25.0%	Recessive	0.590

			No	4	5	3	Homozygous	0.576
				50.0%	41.7%	75.0%	Heterozygous	1.000
**D**	rs209130	TRIM27	relapse	TT	CT	CC	Additive	0.580
			Yes	6	6	0	Dominant	1.000
				50.0%	54.5%	0.0%	Recessive	1.000
			No	6	5	1	Homozygous	1.000
				50.0%	45.5%	100.0%	Heterozygous	0.827
**D**	rs1536215	TRIM27	relapse	CC	CG	GG	Additive	1.000
			Yes	8	4	0	Dominant	1.000
				47.1%	57.1%	0.0%	Recessive	NA
			No	9	3	0	Homozygous	NA
				52.9%	42.9%	0.0%	Heterozygous	1.000
**D**	rs139791445	TRIM27	relapse	CC	CG	GG	Additive	1.000
			Yes	11	1	0	Dominant	1.000
				47.8%	100.0%	0.0%	Recessive	NA
			No	12	0	0	Homozygous	NA
				52.2%	0.0%	0.0%	Heterozygous	1.000
**D**	rs11244	HLA-DOB	relapse	GG	AG	AA	Additive	0.809
			Yes	5	6	1	Dominant	1.000
				50.0%	54.5%	33.3%	Recessive	1.000
			No	5	5	2	Homozygous	1.000
				50.0%	45.5%	66.7%	Heterozygous	1.000
**D**	rs2070120	HLA-DOB	relapse	GG	AG	AA	Additive	1.000
			Yes	11	1	0	Dominant	1.000
				47.8%	100.0%	0.0%	Recessive	NA
			No	12	0	0	Homozygous	NA
				52.2%	0.0%	0.0%	Heterozygous	1.000
**D**	rs56150445	HLA-DOB	relapse	GG	CG	CC	Additive	NA
			Yes	12	0	0	Dominant	NA
				50.0%	0.0%	0.0%	Recessive	NA
			No	12	0	0	Homozygous	NA
				50.0%	0.0%	0.0%	Heterozygous	NA
**D**	rs41258084	HLA-DOB	relapse	CC	CT	TT	Additive	1.000
			Yes	11	1	0	Dominant	1.000
				50.0%	50.0%	0.0%	Recessive	NA
			No	11	1	0	Homozygous	NA
				50.0%	50.0%	0.0%	Heterozygous	1.000
**D**	rs17220087	HLA-DOB	relapse	CC	AC	AA	Additive	1.000
			Yes	11	1	0	Dominant	1.000
				47.8%	100.0%	0.0%	Recessive	NA
			No	12	0	0	Homozygous	NA
				52.2%	0.0%	0.0%	Heterozygous	1.000
**D**	rs2071479	HLA-DOB	relapse	CC	CT	TT	Additive	1.000
			Yes	11	1	0	Dominant	1.000
				47.8%	100.0%	0.0%	Recessive	NA

			No	12	0	0	Homozygous	NA
				52.2%	0.0%	0.0%	Heterozygous	1.000
**D**	rs17213693	HLA-DOB	relapse	GG	CG	CC	Additive	1.000
			Yes	11	1	0	Dominant	1.000
				47.8%	100.0%	0.0%	Recessive	NA
			No	12	0	0	Homozygous	NA
				52.2%	0.0%	0.0%	Heterozygous	1.000
**D**	rs3130048	BAG6	relapse	TT	CT	CC	Additive	1.000
			Yes	7	3	2	Dominant	1.000
				50.0%	50.0%	50.0%	Recessive	1.000
			No	7	3	2	Homozygous	1.000
				50.0%	50.0%	50.0%	Heterozygous	1.000
**D**	rs2844464	BAG6	relapse	GG	AG	AA	Additive	0.317
			Yes	11	1	0	Dominant	0.317
				57.9%	20.0%	0.0%	Recessive	NA
			No	8	4	0	Homozygous	NA
				42.1%	80.0%	0.0%	Heterozygous	0.317
**D**	rs2242656	BAG6	relapse	TT	CT	CC	Additive	0.317
			Yes	11	1	0	Dominant	0.317
				57.9%	20.0%	0.0%	Recessive	NA
			No	8	4	0	Homozygous	NA
				42.1%	80.0%	0.0%	Heterozygous	0.317
**M**	rs107822	RING1	relapse		matched		unmatched	**0.006**
			Yes		9		3	
					81.8%		25.0%	
			No		2		9	
					18.2%		75.0%	
**M**	rs213210	RING1	relapse		matched		unmatched	0.371
			Yes		5		7	
					71.4%		43.8%	
			No		2		9	
					28.6%		56.3%	
**M**	rs209132	TRIM27	relapse		matched		unmatched	0.089
			Yes		10		2	
					66.7%		22.2%	
			No		5		7	
					33.3%		77.8%	
**M**	rs209131	TRIM27	relapse		matched		unmatched	0.408
			Yes		8		4	
					57.1%		40.0%	
			No		6		6	
					42.9%		60.0%	
**M**	rs209130	TRIM27	relapse		matched		unmatched	0.098
			Yes		9		3	
					64.3%		30.0%	

			No	5	7	
				35.7%	70.0%	
**M**	rs1536215	TRIM27	relapse	matched	unmatched	1.000
			Yes	10	2	
				50.0%	50.0%	
			No	10	2	
				50.0%	50.0%	
**M**	rs139791445	TRIM27	relapse	matched	unmatched	1.000
			Yes	11	1	
				47.8%	100.0%	
			No	12	0	
				52.2%	0.0%	
**M**	rs11244	HLA-DOB	relapse	matched	unmatched	1.000
			Yes	6	5	
				46.2%	50.0%	
			No	7	5	
				53.8%	50.0%	
**M**	rs2070120	HLA-DOB	relapse	matched	unmatched	1.000
			Yes	11	0	
				50.0%	0.0%	
			No	11	1	
				50.0%	100.0%	
**M**	rs56150445	HLA-DOB	relapse	matched	unmatched	NA
			Yes	11	0	
				47.8%	0.0%	
			No	12	0	
				52.2%	0.0%	
**M**	rs41258084	HLA-DOB	relapse	matched	unmatched	NA
			Yes	11	0	
				47.8%	0.0%	
			No	12	0	
				52.2%	0.0%	
**M**	rs17220087	HLA-DOB	relapse	matched	unmatched	1.000
			Yes	10	1	
				47.6%	50.0%	
			No	11	1	
				52.4%	50.0%	
**M**	rs2071479	HLA-DOB	relapse	matched	unmatched	1.000
			Yes	11	0	
				50.0%	0.0%	
			No	11	1	
				50.0%	100.0%	
**M**	rs17213693	HLA-DOB	relapse	matched	unmatched	1.000
			Yes	11	0	
				50.0%	0.0%	

			No	11	1	
				50.0%	100.0%	
**M**	rs3130048	BAG6	relapse	matched	unmatched	0.682
			Yes	6	6	
				46.2%	54.5%	
			No	7	5	
				53.8%	45.5%	
**M**	rs2844464	BAG6	relapse	matched	unmatched	1.000
			Yes	9	3	
				52.9%	42.9%	
			No	8	4	
				47.1%	57.1%	
**M**	rs2242656	BAG6	relapse	matched	unmatched	0.640
			Yes	10	2	
				55.6%	33.3%	
			No	8	4	
				44.4%	66.7%	

**Source of DNA[a]**	**SNP**	**Gene**	**Outcome /status**	**Number of patients**			**Model**	***p*-value**
**D**	rs11571315	CTLA4	relapse	TT	CT	CC	Additive	0.584
			Yes	7	5	0	Dominant	0.682
				53.8%	50.0%	0.0%	Recessive	1.000
			No	6	5	1	Homozygous	1.000
				46.2%	50.0%	100.0%	Heterozygous	1.000
**D**	rs733618	CTLA4	relapse	CC	CT	TT	Additive	1.000
			Yes	3	7	2	Dominant	1.000
				50.0%	50.0%	50.0%	Recessive	1.000
			No	3	7	2	Homozygous	1.000
				50.0%	50.0%	50.0%	Heterozygous	1.000
**D**	rs4553808	CTLA4	relapse	AA	AG	GG	Additive	1.000
			Yes	11	1	0	Dominant	1.000
				52.4%	33.3%	0.0%	Recessive	NA
			No	10	2	0	Homozygous	NA
				47.6%	66.7%	0.0%	Heterozygous	1.000
**D**	rs11571316	CTLA4	relapse	GG	AG	AA	Additive	1.000
			Yes	7	5	0	Dominant	1.000
				50.0%	50.0%	0.0%	Recessive	NA
			No	7	5	0	Homozygous	NA
				50.0%	50.0%	0.0%	Heterozygous	1.000
**D**	rs62182595	CTLA4	relapse	GG	AG	AA	Additive	1.000
			Yes	11	1	0	Dominant	1.000
				52.4%	33.3%	0.0%	Recessive	NA
			No	10	2	0	Homozygous	NA
				47.6%	66.7%	0.0%	Heterozygous	1.000
**D**	rs573554201	CTLA4	relapse	AA	AG	GG	Additive	NA
			Yes	12	0	0	Dominant	NA

				50.0%	0.0%	0.0%	Recessive	NA
			No	12	0	0	Homozygous	NA
				50.0%	0.0%	0.0%	Heterozygous	NA
**D**	rs16840252	CTLA4	relapse	CC	CT	TT	Additive	1.000
			Yes	11	1	0	Dominant	1.000
				52.4%	33.3%	0.0%	Recessive	NA
			No	10	2	0	Homozygous	NA
				47.6%	66.7%	0.0%	Heterozygous	1.000
**D**	rs945677329	CTLA4	relapse	AA	AG	GG	Additive	1.000
			Yes	12	0	0	Dominant	1.000
				52.2%	0.0%	0.0%	Recessive	NA
			No	11	1	0	Homozygous	NA
				47.8%	100.0%	0.0%	Heterozygous	1.000
**D**	rs5742909	CTLA4	relapse	CC	CT	TT	Additive	1.000
			Yes	10	2	0	Dominant	1.000
				52.6%	40.0%	0.0%	Recessive	NA
			No	9	3	0	Homozygous	NA
				47.4%	60.0%	0.0%	Heterozygous	1.000
**D**	rs231775	CTLA4	relapse	GG	AG	AA	Additive	0.580
			Yes	6	6	0	Dominant	0.682
				54.5%	50.0%	0.0%	Recessive	1.000
			No	5	6	1	Homozygous	1.000
				45.5%	50.0%	100.0%	Heterozygous	0.827
**D**	rs56102377	CTLA4	relapse	GG	AG	AA	Additive	0.478
			Yes	10	2	0	Dominant	0.478
				45.5%	100.0%	0.0%	Recessive	NA
			No	12	0	0	Homozygous	NA
				54.5%	0.0%	0.0%	Heterozygous	0.478
**D**	rs56217811	CTLA4	relapse	CC	CA	AA	Additive	NA
			Yes	12	0	0	Dominant	NA
				50.0%	0.0%	0.0%	Recessive	NA
			No	12	0	0	Homozygous	NA
				50.0%	0.0%	0.0%	Heterozygous	NA
**D**	rs55696217	CTLA4	relapse	GG	GA	AA	Additive	NA
			Yes	12	0	0	Dominant	NA
				50.0%	0.0%	0.0%	Recessive	NA
			No	12	0	0	Homozygous	NA
				50.0%	0.0%	0.0%	Heterozygous	NA
**D**	rs231721	CTLA4	relapse	TT	CT	CC	Additive	NA
			Yes	12	0	0	Dominant	NA
				50.0%	0.0%	0.0%	Recessive	NA
			No	12	0	0	Homozygous	NA
				50.0%	0.0%	0.0%	Heterozygous	NA
**D**	rs778932058	CTLA4	relapse	AA	AG	GG	Additive	NA
			Yes	12	0	0	Dominant	NA

				50.0%	0.0%	0.0%	Recessive	NA
			No	12	0	0	Homozygous	NA
				50.0%	0.0%	0.0%	Heterozygous	NA
**D**	rs3087243	CTLA4	relapse	GG	AG	AA	Additive	0.682
			Yes	7	5	0	Dominant	0.682
				53.8%	45.5%	0.0%	Recessive	NA
			No	6	6	0	Homozygous	NA
				46.2%	54.5%	0.0%	Heterozygous	0.682
**D**	rs11571319	CTLA4	relapse	GG	AG	AA	Additive	0.590
			Yes	11	1	0	Dominant	0.590
				55.0%	25.0%	0.0%	Recessive	NA
			No	9	3	0	Homozygous	NA
				45.0%	75.0%	0.0%	Heterozygous	0.590
**D**	rs1234314	TNFSF4	relapse	CC	CG	GG	Additive	0.102
			Yes	3	8	1	Dominant	0.667
				37.5%	72.7%	20.0%	Recessive	0.317
			No	5	3	4	Homozygous	1.000
				62.5%	27.3%	80.0%	Heterozygous	0.181
**D**	rs45454293	TNFSF4	relapse	CC	CT	TT	Additive	0.901
			Yes	8	3	1	Dominant	1.000
				53.3%	42.9%	50.0%	Recessive	1.000
			No	7	4	1	Homozygous	1.000
				46.7%	57.1%	50.0%	Heterozygous	1.000
**D**	rs181758110	TNFSF4	relapse	CC	CT	TT	Additive	NA
			Yes	12	0	0	Dominant	NA
				50.0%	0.0%	0.0%	Recessive	NA
			No	12	0	0	Homozygous	NA
				50.0%	0.0%	0.0%	Heterozygous	NA
**D**	rs1879877	CD28	relapse	TT	GT	GG	Additive	0.128
			Yes	2	9	1	Dominant	0.089
				25.0%	64.3%	100.0%	Recessive	1.000
			No	6	5	0	Homozygous	0.333
				75.0%	35.7%	0.0%	Heterozygous	0.183
**D**	rs3181096	CD28	relapse	CC	CT	TT	Additive	0.400
			Yes	6	6	0	Dominant	0.400
				42.9%	66.7%	0.0%	Recessive	NA
			No	8	3	0	Homozygous	NA
				57.1%	33.3%	0.0%	Heterozygous	0.400
**D**	rs3181097	CD28	relapse	AA	AG	GG	Additive	0.163
			Yes	2	7	3	Dominant	0.371
				33.3%	50.0%	100.0%	Recessive	0.217
			No	4	7	0	Homozygous	0.167
				66.7%	50.0%	0.0%	Heterozygous	0.642
**D**	rs3181098	CD28	relapse	GG	AG	AA	Additive	0.400
			Yes	6	6	0	Dominant	0.400

				42.9%	66.7%	0.0%	Recessive	NA
			No	8	3	0	Homozygous	NA
				57.1%	33.3%	0.0%	Heterozygous	0.400
**D**	rs28718975	CD28	relapse	TT	CT	CC	Additive	0.144
			Yes	7	5	0	Dominant	0.371
				43.8%	83.3%	0.0%	Recessive	0.478
			No	9	1	1	Homozygous	1.000
				56.3%	16.7%	100.0%	Heterozygous	0.162
**D**	rs28688913	CD28	relapse	CC	CT	TT	Additive	0.371
			Yes	7	5	0	Dominant	0.371
				43.8%	71.4%	0.0%	Recessive	NA
			No	9	2	0	Homozygous	NA
				56.3%	28.6%	0.0%	Heterozygous	0.371
**D**	rs28541784	CD28	relapse	CC	CT	TT	Additive	0.155
			Yes	7	5	0	Dominant	0.155
				41.2%	83.3%	0.0%	Recessive	NA
			No	10	1	0	Homozygous	NA
				58.8%	16.7%	0.0%	Heterozygous	0.155
**D**	rs201801072	CD28	relapse	TT	CT	CC	Additive	0.371
			Yes	7	5	0	Dominant	0.371
				43.8%	71.4%	0.0%	Recessive	NA
			No	9	2	0	Homozygous	NA
				56.3%	28.6%	0.0%	Heterozygous	0.371
**D**	rs200353921	CD28	relapse	TT	AT	AA	Additive	0.371
			Yes	7	5	0	Dominant	0.371
				43.8%	71.4%	0.0%	Recessive	NA
			No	9	2	0	Homozygous	NA
				56.3%	28.6%	0.0%	Heterozygous	0.371
**D**	rs56228674	CD28	relapse	CC	CT	TT	Additive	0.478
			Yes	10	2	0	Dominant	0.478
				47.6%	100.0%	0.0%	Recessive	NA
			No	11	0	0	Homozygous	NA
				52.4%	0.0%	0.0%	Heterozygous	0.478
**D**	rs1290180288	CD28	relapse	TT	CT	CC	Additive	NA
			Yes	12	0	0	Dominant	NA
				52.2%	0.0%	0.0%	Recessive	NA
			No	11	0	0	Homozygous	NA
				47.8%	0.0%	0.0%	Heterozygous	NA
**D**	rs3116496	CD28	relapse	TT	CT	CC	Additive	0.478
			Yes	10	2	0	Dominant	0.478
				47.6%	100.0%	0.0%	Recessive	NA
			No	11	0	0	Homozygous	NA
				52.4%	0.0%	0.0%	Heterozygous	0.478
**D**	rs375758971	CD28	relapse	TT	CT	CC	Additive	NA
			Yes	12	0	0	Dominant	NA

				52.2%	0.0%	0.0%	Recessive	NA
			No	11	0	0	Homozygous	NA
				47.8%	0.0%	0.0%	Heterozygous	NA
**D**	rs36084323	PD1	relapse	CC	CT	TT	Additive	**0.042**
			Yes	1	6	5	Dominant	0.069
				14.3%	54.5%	83.3%	Recessive	0.155
			No	6	5	1	Homozygous	0.069
				85.7%	45.5%	16.7%	Heterozygous	0.151
**D**	rs5839828	PD1	relapse	del	delG	GG	Additive	**0.014**
			Yes	6	6	0	Dominant	0.069
				85.7%	50.0%	0.0%	Recessive	**0.037**
			No	1	6	5	Homozygous	**0.015**
				14.3%	50.0%	100.0%	Heterozygous	0.173
**D**	rs11568821	PD1	relapse	CC	CA	AA	Additive	NA
			Yes	12	0	0	Dominant	NA
				50.0%	0.0%	0.0%	Recessive	NA
			No	12	0	0	Homozygous	NA
				50.0%	0.0%	0.0%	Heterozygous	NA
**D**	rs41386349	PD1	relapse	GG	AG	AA	Additive	0.281
			Yes	9	2	1	Dominant	0.667
				56.3%	28.6%	100.0%	Recessive	1.000
			No	7	5	0	Homozygous	1.000
				43.8%	71.4%	0.0%	Heterozygous	0.371
**D**	rs6705653	PD1	relapse	CC	CT	TT	Additive	0.341
			Yes	7	4	1	Dominant	0.414
				58.3%	36.4%	100.0%	Recessive	1.000
			No	5	7	0	Homozygous	0.414
				41.7%	63.6%	0.0%	Heterozygous	0.292
**D**	rs2227982	PD1	relapse	GG	AG	AA	Additive	0.072
			Yes	1	6	5	Dominant	0.069
				14.3%	60.0%	71.4%	Recessive	0.371
			No	6	4	2	Homozygous	0.103
				85.7%	40.0%	28.6%	Heterozygous	0.134

[a]P: patient; D: donor; M: patient and donor mismatch status.
