# Supplementary material for: Preliminary Data on SNP of Transplantation-Related Genes after Haploidentical Stem Cell Transplantation
Source: J Clin Med. 2024 Aug 9;13(16):4681. doi: 10.3390/jcm13164681 (PMC11354871; doi:10.3390/jcm13164681)
Supplement: Supplementary file 1 [file jcm-13-04681-s001.zip › Supplementary Table 4_20240718.pdf]

Table S4. Association analysis between SNPs and GVHD status post-haplo-HSCT.

Source of DNA[a]	SNP	Gene	Outcome /status	Number of patients			Model	*p*-value
**P**	rs9276982	HLA-DOA	GVHD	GG	AG	AA	Additive	0.673
			Yes	10	4	0	Dominant	0.673
				62.5%	50.0%	0.0%	Recessive	NA
			No	6	4	0	Homozygous	NA
				37.5%	50.0%	0.0%	Heterozygous	0.673
**P**	rs71565361	HLA-DOA	GVHD	CC	CA	AA	Additive	1.000
			Yes	13	1	0	Dominant	1.000
				56.5%	100.0%	0.0%	Recessive	NA
			No	10	0	0	Homozygous	NA
				43.5%	0.0%	0.0%	Heterozygous	1.000
**P**	rs79327197	HLA-DOA	GVHD	AA	AG	GG	Additive	1.000
			Yes	12	2	0	Dominant	1.000
				60.0%	50.0%	0.0%	Recessive	NA
			No	8	2	0	Homozygous	NA
				40.0%	50.0%	0.0%	Heterozygous	1.000
**P**	rs151190962	HLA-DOA	GVHD	AA	delA	del	Additive	1.000
			Yes	13	1	0	Dominant	1.000
				56.5%	100.0%	0.0%	Recessive	NA
			No	10	0	0	Homozygous	NA
				43.5%	0.0%	0.0%	Heterozygous	1.000
**P**	rs9282369	HLA-DOA	GVHD	ins	insT	TT	Additive	0.319
			Yes	4	7	3	Dominant	0.403
				44.4%	77.8%	50.0%	Recessive	0.665
			No	5	2	3	Homozygous	1.000
				55.6%	22.2%	50.0%	Heterozygous	0.335
**P**	rs2009658	LTA	GVHD	CC	CG	GG	Additive	0.204
			Yes	11	3	0	Dominant	0.204
				68.8%	37.5%	0.0%	Recessive	NA
			No	5	5	0	Homozygous	NA
				31.3%	62.5%	0.0%	Heterozygous	0.204
**P**	rs111250247	LTA	GVHD	TT	TA	AA	Additive	NA
			Yes	14	0	0	Dominant	NA
				58.3%	0.0%	0.0%	Recessive	NA
			No	10	0	0	Homozygous	NA
				41.7%	0.0%	0.0%	Heterozygous	NA
**P**	rs915654	LTA	GVHD	AA	TA	TT	Additive	0.640
			Yes	2	10	2	Dominant	0.615
				40.0%	62.5%	66.7%	Recessive	1.000
			No	3	6	1	Homozygous	1.000
				60.0%	37.5%	33.3%	Heterozygous	0.611
**P**	rs34324979	HSPA1L	GVHD	GG	Gins	ins	Additive	0.665
			Yes	11	3	0	Dominant	0.665

				61.1%	50.0%	0.0%	Recessive	NA
			No	7	3	0	Homozygous	NA
				38.9%	50.0%	0.0%	Heterozygous	0.665
**P**	rs2075800	HSPA1L	GVHD	CC	CT	TT	Additive	0.783
			Yes	3	9	2	Dominant	0.665
				50.0%	64.3%	50.0%	Recessive	1.000
			No	3	5	2	Homozygous	1.000
				50.0%	35.7%	50.0%	Heterozygous	0.642
**P**	rs2227956	HSPA1L	GVHD	AA	GA	GG	Additive	1.000
			Yes	12	2	0	Dominant	1.000
				60.0%	50.0%	0.0%	Recessive	NA
			No	8	2	0	Homozygous	NA
				40.0%	50.0%	0.0%	Heterozygous	1.000
**P**	rs107822	RING1	GVHD	TT	CT	CC	Additive	0.575
			Yes	9	4	1	Dominant	1.000
				64.3%	50.0%	100.0%	Recessive	1.000
			No	5	4	0	Homozygous	1.000
				35.7%	50.0%	0.0%	Heterozygous	0.662
**P**	rs213210	RING1	GVHD	GG	AG	AA	Additive	0.431
			Yes	8	6	0	Dominant	1.000
				61.5%	66.7%	0.0%	Recessive	0.391
			No	5	3	1	Homozygous	0.429
				38.5%	33.3%	100.0%	Heterozygous	1.000
**P**	rs209132	TRIM27	GVHD	GG	AG	AA	Additive	0.643
			Yes	8	5	1	Dominant	1.000
				61.5%	62.5%	33.3%	Recessive	0.550
			No	5	3	2	Homozygous	0.550
				38.5%	37.5%	66.7%	Heterozygous	1.000
**P**	rs209131	TRIM27	GVHD	GG	AG	AA	Additive	0.938
			Yes	5	6	3	Dominant	1.000
				62.5%	54.5%	60.0%	Recessive	1.000
			No	3	5	2	Homozygous	1.000
				37.5%	45.5%	40.0%	Heterozygous	1.000
**P**	rs209130	TRIM27	GVHD	TT	CT	CC	Additive	0.211
			Yes	8	6	0	Dominant	1.000
				61.5%	66.7%	0.0%	Recessive	0.163
			No	5	3	2	Homozygous	0.200
				38.5%	33.3%	100.0%	Heterozygous	1.000
**P**	rs1536215	TRIM27	GVHD	CC	CG	GG	Additive	0.653
			Yes	9	5	0	Dominant	0.653
				52.9%	71.4%	0.0%	Recessive	NA
			No	8	2	0	Homozygous	NA
				47.1%	28.6%	0.0%	Heterozygous	0.653
**P**	rs139791445	TRIM27	GVHD	CC	CG	GG	Additive	NA
			Yes	14	0	0	Dominant	NA

				58.3%	0.0%	0.0%	Recessive	NA
			No	10	0	0	Homozygous	NA
				41.7%	0.0%	0.0%	Heterozygous	NA
**P**	rs11244	HLA-DOB	GVHD	GG	AG	AA	Additive	0.981
			Yes	8	4	1	Dominant	1.000
				57.1%	57.1%	50.0%	Recessive	1.000
			No	6	3	1	Homozygous	1.000
				42.9%	42.9%	50.0%	Heterozygous	1.000
**P**	rs2070120	HLA-DOB	GVHD	GG	AG	AA	Additive	1.000
			Yes	11	2	0	Dominant	1.000
				55.0%	66.7%	0.0%	Recessive	NA
			No	9	1	0	Homozygous	NA
				45.0%	33.3%	0.0%	Heterozygous	1.000
**P**	rs56150445	HLA-DOB	GVHD	GG	CG	CC	Additive	NA
			Yes	13	0	0	Dominant	NA
				56.5%	0.0%	0.0%	Recessive	NA
			No	10	0	0	Homozygous	NA
				43.5%	0.0%	0.0%	Heterozygous	NA
**P**	rs41258084	HLA-DOB	GVHD	CC	CT	TT	Additive	1.000
			Yes	12	1	0	Dominant	1.000
				57.1%	50.0%	0.0%	Recessive	NA
			No	9	1	0	Homozygous	NA
				42.9%	50.0%	0.0%	Heterozygous	1.000
**P**	rs17220087	HLA-DOB	GVHD	CC	AC	AA	Additive	1.000
			Yes	11	2	0	Dominant	1.000
				55.0%	66.7%	0.0%	Recessive	NA
			No	9	1	0	Homozygous	NA
				45.0%	33.3%	0.0%	Heterozygous	1.000
**P**	rs2071479	HLA-DOB	GVHD	CC	CT	TT	Additive	0.435
			Yes	13	0	0	Dominant	0.435
				59.1%	0.0%	0.0%	Recessive	NA
			No	9	1	0	Homozygous	NA
				40.9%	100.0%	0.0%	Heterozygous	0.435
**P**	rs17213693	HLA-DOB	GVHD	GG	CG	CC	Additive	0.604
			Yes	10	3	0	Dominant	0.604
				52.6%	75.0%	0.0%	Recessive	NA
			No	9	1	0	Homozygous	NA
				47.4%	25.0%	0.0%	Heterozygous	0.604
**P**	rs3130048	BAG6	GVHD	TT	CT	CC	Additive	0.435
			Yes	8	6	0	Dominant	1.000
				57.1%	66.7%	0.0%	Recessive	0.417
			No	6	3	1	Homozygous	0.467
				42.9%	33.3%	100.0%	Heterozygous	1.000
**P**	rs2844464	BAG6	GVHD	GG	AG	AA	Additive	0.481
			Yes	11	3	0	Dominant	0.665

				61.1%	60.0%	0.0%	Recessive	0.417
			No	7	2	1	Homozygous	0.421
				38.9%	40.0%	100.0%	Heterozygous	1.000
**P**	rs2242656	BAG6	GVHD	TT	CT	CC	Additive	1.000
			Yes	11	3	0	Dominant	1.000
				57.9%	60.0%	0.0%	Recessive	NA
			No	8	2	0	Homozygous	NA
				42.1%	40.0%	0.0%	Heterozygous	1.000
**D**	rs61365987	NOTCH4	GVHD	CC	CT	TT	Additive	0.239
			Yes	11	3	0	Dominant	0.239
				52.4%	100.0%	0.0%	Recessive	NA
			No	10	0	0	Homozygous	NA
				47.6%	0.0%	0.0%	Heterozygous	0.239
**D**	rs444472	NOTCH4	GVHD	GG	GA	AA	Additive	0.688
			Yes	9	4	1	Dominant	1.000
				56.3%	57.1%	100.0%	Recessive	1.000
			No	7	3	0	Homozygous	1.000
				43.8%	42.9%	0.0%	Heterozygous	1.000
**D**	rs2256594	NOTCH4	GVHD	AA	AG	GG	Additive	0.418
			Yes	5	6	3	Dominant	0.653
				71.4%	46.2%	75.0%	Recessive	0.615
			No	2	7	1	Homozygous	1.000
				28.6%	53.8%	25.0%	Heterozygous	0.374
**D**	rs394657	NOTCH4	GVHD	AA	AG	GG	Additive	0.688
			Yes	9	4	1	Dominant	1.000
				56.3%	57.1%	100.0%	Recessive	1.000
			No	7	3	0	Homozygous	1.000
				43.8%	42.9%	0.0%	Heterozygous	1.000
**D**	rs429853	NOTCH4	GVHD	TT	TC	CC	Additive	0.653
			Yes	9	5	0	Dominant	0.653
				52.9%	71.4%	0.0%	Recessive	NA
			No	8	2	0	Homozygous	NA
				47.1%	28.6%	0.0%	Heterozygous	0.653
**D**	rs111394117	NOTCH4	GVHD	GG	GA	AA	Additive	0.550
			Yes	13	1	0	Dominant	0.550
				61.9%	33.3%	0.0%	Recessive	NA
			No	8	2	0	Homozygous	NA
				38.1%	66.7%	0.0%	Heterozygous	0.550
**D**	rs9281491	HCP5	GVHD	ins	insA	AA	Additive	0.395
			Yes	11	3	0	Dominant	0.393
				64.7%	50.0%	0.0%	Recessive	0.417
			No	6	3	1	Homozygous	0.389
				35.3%	50.0%	100.0%	Heterozygous	0.643
**D**	rs2244546	HCP5	GVHD	CC	CG	GG	Additive	1.000
			Yes	9	5	0	Dominant	1.000

				56.3%	62.5%	0.0%	Recessive	NA
			No	7	3	0	Homozygous	NA
				43.8%	37.5%	0.0%	Heterozygous	1.000
**D**	rs4713466	HCP5	GVHD	CC	CT	TT	Additive	0.240
			Yes	6	8	0	Dominant	0.240
				46.2%	72.7%	0.0%	Recessive	NA
			No	7	3	0	Homozygous	NA
				53.8%	27.3%	0.0%	Heterozygous	0.240
**D**	rs2523676	HCP5	GVHD	CC	CT	TT	Additive	**0.026**
			Yes	4	10	0	Dominant	0.211
				40.0%	83.3%	0.0%	Recessive	0.163
			No	6	2	2	Homozygous	0.515
				60.0%	16.7%	100.0%	Heterozygous	0.074
**D**	rs2523675	HCP5	GVHD	GG	GA	AA	Additive	0.504
			Yes	3	10	1	Dominant	0.665
				50.0%	66.7%	33.3%	Recessive	0.550
			No	3	5	2	Homozygous	1.000
				50.0%	33.3%	66.7%	Heterozygous	0.631
**D**	rs2518028	HCP5	GVHD	CC	TC	TT	Additive	0.615
			Yes	12	2	0	Dominant	0.615
				63.2%	40.0%	0.0%	Recessive	NA
			No	7	3	0	Homozygous	NA
				36.8%	60.0%	0.0%	Heterozygous	0.615
**D**	rs141431529	HCP5	GVHD	GG	GT	TT	Additive	1.000
			Yes	12	2	0	Dominant	1.000
				60.0%	50.0%	0.0%	Recessive	NA
			No	8	2	0	Homozygous	NA
				40.0%	50.0%	0.0%	Heterozygous	1.000
**D**	rs107822	RING1	GVHD	TT	CT	CC	Additive	0.710
			Yes	6	6	2	Dominant	0.408
				50.0%	66.7%	66.7%	Recessive	1.000
			No	6	3	1	Homozygous	1.000
				50.0%	33.3%	33.3%	Heterozygous	0.685
**D**	rs213210	RING1	GVHD	GG	AG	AA	Additive	0.977
			Yes	5	6	3	Dominant	1.000
				55.6%	60.0%	60.0%	Recessive	1.000
			No	4	4	2	Homozygous	1.000
				44.4%	40.0%	40.0%	Heterozygous	1.000
**D**	rs209132	TRIM27	GVHD	GG	AG	AA	Additive	0.883
			Yes	6	7	1	Dominant	1.000
				54.5%	63.6%	50.0%	Recessive	1.000
			No	5	4	1	Homozygous	1.000
				45.5%	36.4%	50.0%	Heterozygous	1.000
**D**	rs209131	TRIM27	GVHD	GG	AG	AA	Additive	0.710

			Yes	4	7	3	Dominant	0.673
				50.0%	58.3%	75.0%	Recessive	0.615
			No	4	5	1	Homozygous	0.573
				50.0%	41.7%	25.0%	Heterozygous	1.000
**D**	rs209130	TRIM27	GVHD	TT	CT	CC	Additive	0.466
			Yes	7	7	0	Dominant	1.000
				58.3%	63.6%	0.0%	Recessive	0.417
			No	5	4	1	Homozygous	0.462
				41.7%	36.4%	100.0%	Heterozygous	1.000
**D**	rs1536215	TRIM27	GVHD	CC	CG	GG	Additive	0.653
			Yes	9	5	0	Dominant	0.653
				52.9%	71.4%	0.0%	Recessive	NA
			No	8	2	0	Homozygous	NA
				47.1%	28.6%	0.0%	Heterozygous	0.653
**D**	rs139791445	TRIM27	GVHD	CC	CG	GG	Additive	0.417
			Yes	14	0	0	Dominant	0.417
				60.9%	0.0%	0.0%	Recessive	NA
			No	9	1	0	Homozygous	NA
				39.1%	100.0%	0.0%	Heterozygous	0.417
**D**	rs11244	HLA-DOB	GVHD	GG	AG	AA	Additive	0.053
			Yes	3	9	2	Dominant	0.035
				30.0%	81.8%	66.7%	Recessive	1.000
			No	7	2	1	Homozygous	0.510
				70.0%	18.2%	33.3%	Heterozygous	0.030
**D**	rs2070120	HLA-DOB	GVHD	GG	AG	AA	Additive	0.417
			Yes	14	0	0	Dominant	0.417
				60.9%	0.0%	0.0%	Recessive	NA
			No	9	1	0	Homozygous	NA
				39.1%	100.0%	0.0%	Heterozygous	0.417
**D**	rs56150445	HLA-DOB	GVHD	GG	CG	CC	Additive	NA
			Yes	14	0	0	Dominant	NA
				58.3%	0.0%	0.0%	Recessive	NA
			No	10	0	0	Homozygous	NA
				41.7%	0.0%	0.0%	Heterozygous	NA
**D**	rs41258084	HLA-DOB	GVHD	CC	CT	TT	Additive	1.000
			Yes	13	1	0	Dominant	1.000
				59.1%	50.0%	0.0%	Recessive	NA
			No	9	1	0	Homozygous	NA
				40.9%	50.0%	0.0%	Heterozygous	1.000
**D**	rs17220087	HLA-DOB	GVHD	CC	AC	AA	Additive	0.417
			Yes	14	0	0	Dominant	0.417
				60.9%	0.0%	0.0%	Recessive	NA
			No	9	1	0	Homozygous	NA
				39.1%	100.0%	0.0%	Heterozygous	0.417
**D**	rs2071479	HLA-DOB	GVHD	CC	CT	TT	Additive	1.000

			Yes	13	1	0	Dominant	1.000
				56.5%	100.0%	0.0%	Recessive	NA
			No	10	0	0	Homozygous	NA
				43.5%	0.0%	0.0%	Heterozygous	1.000
**D**	rs17213693	HLA-DOB	GVHD	GG	CG	CC	Additive	0.417
			Yes	14	0	0	Dominant	0.417
				60.9%	0.0%	0.0%	Recessive	NA
			No	9	1	0	Homozygous	NA
				39.1%	100.0%	0.0%	Heterozygous	0.417
**D**	rs3130048	BAG6	GVHD	TT	CT	CC	Additive	0.727
			Yes	8	3	3	Dominant	1.000
				57.1%	50.0%	75.0%	Recessive	0.615
			No	6	3	1	Homozygous	1.000
				42.9%	50.0%	25.0%	Heterozygous	1.000
**D**	rs2844464	BAG6	GVHD	GG	AG	AA	Additive	0.615
			Yes	12	2	0	Dominant	0.615
				63.2%	40.0%	0.0%	Recessive	NA
			No	7	3	0	Homozygous	NA
				36.8%	60.0%	0.0%	Heterozygous	0.615
**D**	rs2242656	BAG6	GVHD	TT	CT	CC	Additive	0.615
			Yes	12	2	0	Dominant	0.615
				63.2%	40.0%	0.0%	Recessive	NA
			No	7	3	0	Homozygous	NA
				36.8%	60.0%	0.0%	Heterozygous	0.615
**M**	rs107822	RING1	GVHD		matched		unmatched	0.680
			Yes		6		8	
					54.5%		66.7%	
			No		5		4	
					45.5%		33.3%	
**M**	rs213210	RING1	GVHD		matched		unmatched	0.363
			Yes		3		11	
					42.9%		68.8%	
			No		4		5	
					57.1%		31.3%	
**M**	rs209132	TRIM27	GVHD		matched		unmatched	0.678
			Yes		8		6	
					53.3%		66.7%	
			No		7		3	
					46.7%		33.3%	
**M**	rs209131	TRIM27	GVHD		matched		unmatched	0.421
			Yes		7		7	
					50.0%		70.0%	
			No		7		3	
					50.0%		30.0%	

**M**	rs209130	TRIM27	GVHD	matched	unmatched	0.421
			Yes	7	7	
				50.0%	70.0%	
			No	7	3	
				50.0%	30.0%	
**M**	rs1536215	TRIM27	GVHD	matched	unmatched	1.000
			Yes	12	2	
				60.0%	50.0%	
			No	8	2	
				40.0%	50.0%	
**M**	rs139791445	TRIM27	GVHD	matched	unmatched	0.417
			Yes	14	0	
				60.9%	0.0%	
			No	9	1	
				39.1%	100.0%	
**M**	rs11244	HLA-DOB	GVHD	matched	unmatched	0.010
			Yes	4	9	
				30.8%	90.0%	
			No	9	1	
				69.2%	10.0%	
**M**	rs2070120	HLA-DOB	GVHD	matched	unmatched	1.000
			Yes	12	1	
				54.5%	100.0%	
			No	10	0	
				45.5%	0.0%	
**M**	rs56150445	HLA-DOB	GVHD	matched	unmatched	NA
			Yes	13	0	
				56.5%	0.0%	
			No	10	0	
				43.5%	0.0%	
**M**	rs41258084	HLA-DOB	GVHD	matched	unmatched	NA
			Yes	13	0	
				56.5%	0.0%	
			No	10	0	
				43.5%	0.0%	
**M**	rs17220087	HLA-DOB	GVHD	matched	unmatched	0.486
			Yes	11	2	
				52.4%	100.0%	
			No	10	0	
				47.6%	0.0%	
**M**	rs2071479	HLA-DOB	GVHD	matched	unmatched	0.435
			Yes	13	0	
				59.1%	0.0%	
			No	9	1	
				40.9%	100.0%	

**M**	rs17213693	HLA-DOB	GVHD		matched		unmatched	1.000
			Yes		12		1	
					54.5%		100.0%	
			No		10		0	
					45.5%		0.0%	
**M**	rs3130048	BAG6	GVHD		matched		unmatched	0.697
			Yes		7		7	
					53.8%		63.6%	
			No		6		4	
					46.2%		36.4%	
**M**	rs2844464	BAG6	GVHD		matched		unmatched	0.393
			Yes		11		3	
					64.7%		42.9%	
			No		6		4	
					35.3%		57.1%	
**M**	rs2242656	BAG6	GVHD		matched		unmatched	0.665
			Yes		11		3	
					61.1%		50.0%	
			No		7		3	
					38.9%		50.0%	
**Source of DNAa**	**SNP**	**Gene**	**Outcome /status**	**Number of patients**			**Model**	***p*-value**
**D**	rs11571315	CTLA4	GVHD	TT	CT	CC	Additive	0.356
			Yes	7	7	0	Dominant	0.697
				53.8%	70.0%	0.0%	Recessive	0.417
			No	6	3	1	Homozygous	1.000
				46.2%	30.0%	100.0%	Heterozygous	0.669
**D**	rs733618	CTLA4	GVHD	CC	CT	TT	Additive	0.225
			Yes	3	10	1	Dominant	0.665
				50.0%	71.4%	25.0%	Recessive	0.272
			No	3	4	3	Homozygous	0.571
				50.0%	28.6%	75.0%	Heterozygous	0.613
**D**	rs4553808	CTLA4	GVHD	AA	AG	GG	Additive	1.000
			Yes	12	2	0	Dominant	1.000
				57.1%	66.7%	0.0%	Recessive	NA
			No	9	1	0	Homozygous	NA
				42.9%	33.3%	0.0%	Heterozygous	1.000
**D**	rs11571316	CTLA4	GVHD	GG	AG	AA	Additive	0.678
			Yes	9	5	0	Dominant	0.678
				64.3%	50.0%	0.0%	Recessive	NA
			No	5	5	0	Homozygous	NA
				35.7%	50.0%	0.0%	Heterozygous	0.678
**D**	rs62182595	CTLA4	GVHD	GG	AG	AA	Additive	1.000
			Yes	12	2	0	Dominant	1.000
				57.1%	66.7%	0.0%	Recessive	NA

			No	9	1	0	Homozygous	NA
				42.9%	33.3%	0.0%	Heterozygous	1.000
**D**	rs573554201	CTLA4	GVHD	AA	AG	GG	Additive	NA
			Yes	14	0	0	Dominant	NA
				58.3%	0.0%	0.0%	Recessive	NA
			No	10	0	0	Homozygous	NA
				41.7%	0.0%	0.0%	Heterozygous	NA
**D**	rs16840252	CTLA4	GVHD	CC	CT	TT	Additive	1.000
			Yes	12	2	0	Dominant	1.000
				57.1%	66.7%	0.0%	Recessive	NA
			No	9	1	0	Homozygous	NA
				42.9%	33.3%	0.0%	Heterozygous	1.000
**D**	rs945677329	CTLA4	GVHD	AA	AG	GG	Additive	1.000
			Yes	13	1	0	Dominant	1.000
				56.5%	100.0%	0.0%	Recessive	NA
			No	10	0	0	Homozygous	NA
				43.5%	0.0%	0.0%	Heterozygous	1.000
**D**	rs5742909	CTLA4	GVHD	CC	CT	TT	Additive	0.615
			Yes	12	2	0	Dominant	0.615
				63.2%	40.0%	0.0%	Recessive	NA
			No	7	3	0	Homozygous	NA
				36.8%	60.0%	0.0%	Heterozygous	0.615
**D**	rs231775	CTLA4	GVHD	GG	AG	AA	Additive	0.391
			Yes	7	7	0	Dominant	0.697
				63.6%	58.3%	0.0%	Recessive	0.417
			No	4	5	1	Homozygous	0.417
				36.4%	41.7%	100.0%	Heterozygous	1.000
**D**	rs56102377	CTLA4	GVHD	GG	AG	AA	Additive	0.493
			Yes	12	2	0	Dominant	0.493
				54.5%	100.0%	0.0%	Recessive	NA
			No	10	0	0	Homozygous	NA
				45.5%	0.0%	0.0%	Heterozygous	0.493
**D**	rs56217811	CTLA4	GVHD	CC	CA	AA	Additive	NA
			Yes	14	0	0	Dominant	NA
				58.3%	0.0%	0.0%	Recessive	NA
			No	10	0	0	Homozygous	NA
				41.7%	0.0%	0.0%	Heterozygous	NA
**D**	rs55696217	CTLA4	GVHD	GG	GA	AA	Additive	NA
			Yes	14	0	0	Dominant	NA
				58.3%	0.0%	0.0%	Recessive	NA
			No	10	0	0	Homozygous	NA
				41.7%	0.0%	0.0%	Heterozygous	NA
**D**	rs231721	CTLA4	GVHD	TT	CT	CC	Additive	NA
			Yes	14	0	0	Dominant	NA
				58.3%	0.0%	0.0%	Recessive	NA

			No	10	0	0	Homozygous	NA
				41.7%	0.0%	0.0%	Heterozygous	NA
**D**	rs778932058	CTLA4	GVHD	AA	AG	GG	Additive	NA
			Yes	14	0	0	Dominant	NA
				58.3%	0.0%	0.0%	Recessive	NA
			No	10	0	0	Homozygous	NA
				41.7%	0.0%	0.0%	Heterozygous	NA
**D**	rs3087243	CTLA4	GVHD	GG	AG	AA	Additive	1.000
			Yes	8	6	0	Dominant	1.000
				61.5%	54.5%	0.0%	Recessive	NA
			No	5	5	0	Homozygous	NA
				38.5%	45.5%	0.0%	Heterozygous	1.000
**D**	rs11571319	CTLA4	GVHD	GG	AG	AA	Additive	1.000
			Yes	12	2	0	Dominant	1.000
				60.0%	50.0%	0.0%	Recessive	NA
			No	8	2	0	Homozygous	NA
				40.0%	50.0%	0.0%	Heterozygous	1.000
**D**	rs1234314	TNFSF4	GVHD	CC	CG	GG	Additive	0.527
			Yes	4	6	4	Dominant	1.000
				50.0%	54.5%	80.0%	Recessive	0.358
			No	4	5	1	Homozygous	0.580
				50.0%	45.5%	20.0%	Heterozygous	1.000
**D**	rs45454293	TNFSF4	GVHD	CC	CT	TT	Additive	0.217
			Yes	7	6	1	Dominant	0.210
				46.7%	85.7%	50.0%	Recessive	1.000
			No	8	1	1	Homozygous	1.000
				53.3%	14.3%	50.0%	Heterozygous	0.165
**D**	rs181758110	TNFSF4	GVHD	CC	CT	TT	Additive	NA
			Yes	14	0	0	Dominant	NA
				58.3%	0.0%	0.0%	Recessive	NA
			No	10	0	0	Homozygous	NA
				41.7%	0.0%	0.0%	Heterozygous	NA
**D**	rs1879877	CD28	GVHD	TT	GT	GG	Additive	0.492
			Yes	5	8	0	Dominant	1.000
				62.5%	57.1%	0.0%	Recessive	0.409
			No	3	6	1	Homozygous	0.429
				37.5%	42.9%	100.0%	Heterozygous	1.000
**D**	rs3181096	CD28	GVHD	CC	CT	TT	Additive	1.000
			Yes	8	5	0	Dominant	1.000
				57.1%	55.6%	0.0%	Recessive	NA
			No	6	4	0	Homozygous	NA
				42.9%	44.4%	0.0%	Heterozygous	1.000
**D**	rs3181097	CD28	GVHD	AA	AG	GG	Additive	0.265
			Yes	5	7	1	Dominant	0.179
				83.3%	50.0%	33.3%	Recessive	0.560

			No	1	7	2	Homozygous	0.226
				16.7%	50.0%	66.7%	Heterozygous	0.325
**D**	rs3181098	CD28	GVHD	GG	AG	AA	Additive	1.000
			Yes	8	5	0	Dominant	1.000
				57.1%	55.6%	0.0%	Recessive	NA
			No	6	4	0	Homozygous	NA
				42.9%	44.4%	0.0%	Heterozygous	1.000
**D**	rs28718975	CD28	GVHD	TT	CT	CC	Additive	0.314
			Yes	10	2	1	Dominant	0.650
				62.5%	33.3%	100.0%	Recessive	1000
			No	6	4	0	Homozygous	1000
				37.5%	66.7%	0.0%	Heterozygous	0.348
**D**	rs28688913	CD28	GVHD	CC	CT	TT	Additive	0.650
			Yes	10	3	0	Dominant	0.650
				62.5%	42.9%	0.0%	Recessive	NA
			No	6	4	0	Homozygous	NA
				37.5%	57.1%	0.0%	Heterozygous	0.650
**D**	rs28541784	CD28	GVHD	CC	CT	TT	Additive	0.341
			Yes	11	2	0	Dominant	0.341
				64.7%	33.3%	0.0%	Recessive	NA
			No	6	4	0	Homozygous	NA
				35.3%	66.7%	0.0%	Heterozygous	0.341
**D**	rs201801072	CD28	GVHD	TT	CT	CC	Additive	0.650
			Yes	10	3	0	Dominant	0.650
				62.5%	42.9%	0.0%	Recessive	NA
			No	6	4	0	Homozygous	NA
				37.5%	57.1%	0.0%	Heterozygous	0.650
**D**	rs200353921	CD28	GVHD	TT	AT	AA	Additive	0.650
			Yes	10	3	0	Dominant	0.650
				62.5%	42.9%	0.0%	Recessive	NA
			No	6	4	0	Homozygous	NA
				37.5%	57.1%	0.0%	Heterozygous	0.650
**D**	rs56228674	CD28	GVHD	CC	CT	TT	Additive	1.000
			Yes	12	1	0	Dominant	1.000
				57.1%	50.0%	0.0%	Recessive	NA
			No	9	1	0	Homozygous	NA
				42.9%	50.0%	0.0%	Heterozygous	1.000
**D**	rs1290180288	CD28	GVHD	TT	CT	CC	Additive	NA
			Yes	13	0	0	Dominant	NA
				56.5%	0.0%	0.0%	Recessive	NA
			No	10	0	0	Homozygous	NA
				43.5%	0.0%	0.0%	Heterozygous	NA
**D**	rs3116496	CD28	GVHD	TT	CT	CC	Additive	1.000
			Yes	12	1	0	Dominant	1.000
				57.1%	50.0%	0.0%	Recessive	NA

			No	9	1	0	Homozygous	NA
				42.9%	50.0%	0.0%	Heterozygous	1.000
**D**	rs375758971	CD28	GVHD	TT	CT	CC	Additive	NA
			Yes	13	0	0	Dominant	NA
				56.5%	0.0%	0.0%	Recessive	NA
			No	10	0	0	Homozygous	NA
				43.5%	0.0%	0.0%	Heterozygous	NA
**D**	rs36084323	PD1	GVHD	CC	CT	TT	Additive	0.407
			Yes	3	8	3	Dominant	0.393
				42.9%	72.7%	50.0%	Recessive	0.665
			No	4	3	3	Homozygous	1.000
				57.1%	27.3%	50.0%	Heterozygous	0.322
**D**	rs5839828	PD1	GVHD	del	delG	GG	Additive	0.595
			Yes	4	8	2	Dominant	1.000
				57.1%	66.7%	40.0%	Recessive	0.615
			No	3	4	3	Homozygous	1.000
				42.9%	33.3%	60.0%	Heterozygous	1.000
**D**	rs11568821	PD1	GVHD	CC	CA	AA	Additive	NA
			Yes	14	0	0	Dominant	NA
				58.3%	0.0%	0.0%	Recessive	NA
			No	10	0	0	Homozygous	NA
				41.7%	0.0%	0.0%	Heterozygous	NA
**D**	rs41386349	PD1	GVHD	GG	AG	AA	Additive	0.137
			Yes	11	2	1	Dominant	0.204
				68.8%	28.6%	100.0%	Recessive	1.000
			No	5	5	0	Homozygous	1.000
				31.3%	71.4%	0.0%	Heterozygous	0.169
**D**	rs6705653	PD1	GVHD	CC	CT	TT	Additive	0.405
			Yes	8	5	1	Dominant	0.408
				66.7%	45.5%	100.0%	Recessive	1.000
			No	4	6	0	Homozygous	1.000
				33.3%	54.5%	0.0%	Heterozygous	0.414
**D**	rs2227982	PD1	GVHD	GG	AG	AA	Additive	0.534
			Yes	3	7	4	Dominant	0.393
				42.9%	70.0%	57.1%	Recessive	1.000
			No	4	3	3	Homozygous	1.000
				57.1%	30.0%	42.9%	Heterozygous	0.350

[a]P: patient; D: donor; M: patient and donor mismatch status.
