# Supplementary material for: Preliminary Data on SNP of Transplantation-Related Genes after Haploidentical Stem Cell Transplantation
Source: J Clin Med. 2024 Aug 9;13(16):4681. doi: 10.3390/jcm13164681 (PMC11354871; doi:10.3390/jcm13164681)
Supplement: Supplementary file 1 [file jcm-13-04681-s001.zip › Supplementary Table 5_20240718.pdf]

Table S5. Association analysis between SNPs and GVHD I-II status post-haplo-HSCT.

Source of DNA[a]	SNP	Gene	Outcome/ status	Number of patients			Model	*p*-value
**P**	rs9276982	HLA-DOA	GVHD I-II	GG	AG	AA	Additive	0.189
			Yes	7	1	0	Dominant	0.189
				43.8%	12.5%	0.0%	Recessive	NA
			No	9	7	0	Homozygous	NA
				56.3%	87.5%	0.0%	Heterozygous	0.189
**P**	rs7156536	HLA-DOA	GVHD I-II	CC	CA	AA	Additive	0.333
			Yes	7	1	0	Dominant	0.333
				30.4%	100.0%	0.0%	Recessive	NA
			No	16	0	0	Homozygous	NA
				69.6%	0.0%	0.0%	Heterozygous	0.333
**P**	rs79327197	HLA-DOA	GVHD I-II	AA	AG	GG	Additive	0.262
			Yes	8	0	0	Dominant	0.262
				40.0%	0.0%	0.0%	Recessive	NA
			No	12	4	0	Homozygous	NA
				60.0%	100.0%	0.0%	Heterozygous	0.262
**P**	rs151190962	HLA-DOA	GVHD I-II	AA	delA	del	Additive	1.000
			Yes	8	0	0	Dominant	1.000
				34.8%	0.0%	0.0%	Recessive	NA
			No	15	1	0	Homozygous	NA
				65.2%	100.0%	0.0%	Heterozygous	1.000
**P**	rs9282369	HLA-DOA	GVHD I-II	ins	insT	TT	Additive	0.535
			Yes	2	3	3	Dominant	0.657
				22.2%	33.3%	50.0%	Recessive	0.362
			No	7	6	3	Homozygous	0.329
				77.8%	66.7%	50.0%	Heterozygous	1.000
**P**	rs2009658	LTA	GVHD I-II	CC	CG	GG	Additive	0.189
			Yes	7	1	0	Dominant	0.189
				43.8%	12.5%	0.0%	Recessive	NA
			No	9	7	0	Homozygous	NA
				56.3%	87.5%	0.0%	Heterozygous	0.189
**P**	rs111250247	LTA	GVHD I-II	TT	TA	AA	Additive	NA
			Yes	8	0	0	Dominant	NA
				33.3%	0.0%	0.0%	Recessive	NA
			No	16	0	0	Homozygous	NA
				66.7%	0.0%	0.0%	Heterozygous	NA
**P**	rs915654	LTA	GVHD I-II	AA	TA	TT	Additive	0.769
			Yes	1	6	1	Dominant	0.631
				20.0%	37.5%	33.3%	Recessive	1.000
			No	4	10	2	Homozygous	1.000
				80.0%	62.5%	66.7%	Heterozygous	0.624
**P**	rs34324979	HSPA1L	GVHD I-II	GG	Gins	ins	Additive	0.621
			Yes	7	1	0	Dominant	0.621
				38.9%	16.7%	0.0%	Recessive	NA
			No	11	5	0	Homozygous	NA
				61.1%	83.3%	0.0%	Heterozygous	0.621
**P**	rs2075800	HSPA1L	GVHD I-II	CC	CT	TT	Additive	0.725

			Yes	2	4	2	Dominant	1.000
				33.3%	28.6%	50.0%	Recessive	0.578
			No	4	10	2	Homozygous	1.000
				66.7%	71.4%	50.0%	Heterozygous	1.000
**P**	rs2227956	HSPA1L	GVHD I-II	AA	GA	GG	Additive	0.262
			Yes	8	0	0	Dominant	0.262
				40.0%	0.0%	0.0%	Recessive	NA
			No	12	4	0	Homozygous	NA
				60.0%	100.0%	0.0%	Heterozygous	0.262
**P**	rs107822	RING1	GVHD I-II	TT	CT	CC	Additive	0.529
			Yes	6	2	0	Dominant	0.400
				42.9%	25.0%	0.0%	Recessive	1.000
			No	8	6	1	Homozygous	1.000
				57.1%	75.0%	100.0%	Heterozygous	0.649
**P**	rs213210	RING1	GVHD I-II	GG	AG	AA	Additive	0.387
			Yes	6	2	0	Dominant	0.379
				46.2%	22.2%	0.0%	Recessive	1.000
			No	7	7	1	Homozygous	1.000
				53.8%	77.8%	100.0%	Heterozygous	0.380
**P**	rs209132	TRIM27	GVHD I-II	GG	AG	AA	Additive	0.424
			Yes	5	3	0	Dominant	0.679
				38.5%	37.5%	0.0%	Recessive	0.526
			No	8	5	3	Homozygous	0.509
				61.5%	62.5%	100.0%	Heterozygous	1.000
**P**	rs209131	TRIM27	GVHD I-II	GG	AG	AA	Additive	0.842
			Yes	3	3	2	Dominant	1.000
				37.5%	27.3%	40.0%	Recessive	1.000
			No	5	8	3	Homozygous	1.000
				62.5%	72.7%	60.0%	Heterozygous	1.000
**P**	rs209130	TRIM27	GVHD I-II	TT	CT	CC	Additive	0.562
			Yes	5	3	0	Dominant	0.679
				38.5%	33.3%	0.0%	Recessive	0.536
			No	8	6	2	Homozygous	0.524
				61.5%	66.7%	100.0%	Heterozygous	1.000
**P**	rs1536215	TRIM27	GVHD I-II	CC	CG	GG	Additive	0.647
			Yes	5	3	0	Dominant	1.000
				29.4%	42.9%	0.0%	Recessive	NA
			No	12	4	0	Homozygous	NA
				70.6%	57.1%	0.0%	Heterozygous	1.000
**P**	rs139791445	TRIM27	GVHD I-II	CC	CG	GG	Additive	NA
			Yes	8	0	0	Dominant	NA
				33.3%	0.0%	0.0%	Recessive	NA
			No	16	0	0	Homozygous	NA
				66.7%	0.0%	0.0%	Heterozygous	NA
**P**	rs11244	HLA-DOB	GVHD I-II	GG	AG	AA	Additive	0.725
			Yes	4	3	1	Dominant	0.657
				28.6%	42.9%	50.0%	Recessive	1.000
			No	10	4	1	Homozygous	1.000
				71.4%	57.1%	50.0%	Heterozygous	0.638

**P**	rs2070120	HLA-DOB	GVHD I-II	GG	AG	AA	Additive	1.000
			Yes	7	1	0	Dominant	1.000
				35.0%	33.3%	0.0%	Recessive	NA
			No	13	2	0	Homozygous	NA
				65.0%	66.7%	0.0%	Heterozygous	1.000
**P**	rs56150445	HLA-DOB	GVHD I-II	GG	CG	CC	Additive	NA
			Yes	8	0	0	Dominant	NA
				34.8%	0.0%	0.0%	Recessive	NA
			No	15	0	0	Homozygous	NA
				65.2%	0.0%	0.0%	Heterozygous	NA
**P**	rs41258084	HLA-DOB	GVHD I-II	CC	CT	TT	Additive	1.000
			Yes	7	1	0	Dominant	1.000
				33.3%	50.0%	0.0%	Recessive	NA
			No	14	1	0	Homozygous	NA
				66.7%	50.0%	0.0%	Heterozygous	1.000
**P**	rs17220087	HLA-DOB	GVHD I-II	CC	AC	AA	Additive	1.000
			Yes	7	1	0	Dominant	1.000
				35.0%	33.3%	0.0%	Recessive	NA
			No	13	2	0	Homozygous	NA
				65.0%	66.7%	0.0%	Heterozygous	1.000
**P**	rs2071479	HLA-DOB	GVHD I-II	CC	CT	TT	Additive	1.000
			Yes	8	0	0	Dominant	1.000
				36.4%	0.0%	0.0%	Recessive	NA
			No	14	1	0	Homozygous	NA
				63.6%	100.0%	0.0%	Heterozygous	1.000
**P**	rs17213693	HLA-DOB	GVHD I-II	GG	CG	CC	Additive	0.589
			Yes	6	2	0	Dominant	0.589
				31.6%	50.0%	0.0%	Recessive	NA
			No	13	2	0	Homozygous	NA
				68.4%	50.0%	0.0%	Heterozygous	0.589
**P**	rs3130048	BAG6	GVHD I-II	TT	CT	CC	Additive	0.456
			Yes	6	2	0	Dominant	0.388
				42.9%	22.2%	0.0%	Recessive	1.000
			No	8	7	1	Homozygous	1.000
				57.1%	77.8%	100.0%	Heterozygous	0.400
**P**	rs2844464	BAG6	GVHD I-II	GG	AG	AA	Additive	0.741
			Yes	6	2	0	Dominant	1.000
				33.3%	40.0%	0.0%	Recessive	1.000
			No	12	3	1	Homozygous	1.000
				66.7%	60.0%	100.0%	Heterozygous	1.000
**P**	rs2242656	BAG6	GVHD I-II	TT	CT	CC	Additive	1.000
			Yes	6	2	0	Dominant	1.000
				31.6%	40.0%	0.0%	Recessive	NA
			No	13	3	0	Homozygous	NA
				68.4%	60.0%	0.0%	Heterozygous	1.000
**D**	rs61365987	NOTCH4	GVHD I-II	CC	CT	TT	Additive	0.249
			Yes	6	2	0	Dominant	0.249
				28.6%	66.7%	0.0%	Recessive	NA
			No	15	1	0	Homozygous	NA
				71.4%	33.3%	0.0%	Heterozygous	0.249

**D**	rs444472	NOTCH4	GVHD I-II	GG	GA	AA	Additive	0.665
			Yes	5	3	0	Dominant	1.000
				31.3%	42.9%	0.0%	Recessive	1.000
			No	11	4	1	Homozygous	1.000
				68.8%	57.1%	100.0%	Heterozygous	0.657
**D**	rs2256594	NOTCH4	GVHD I-II	AA	AG	GG	Additive	0.283
			Yes	4	3	1	Dominant	0.167
				57.1%	23.1%	25.0%	Recessive	1.000
			No	3	10	3	Homozygous	0.545
				42.9%	76.9%	75.0%	Heterozygous	0.174
**D**	rs394657	NOTCH4	GVHD I-II	AA	AG	GG	Additive	0.665
			Yes	5	3	0	Dominant	1.000
				31.3%	42.9%	0.0%	Recessive	1.000
			No	11	4	1	Homozygous	1.000
				68.8%	57.1%	100.0%	Heterozygous	0.657
**D**	rs429853	NOTCH4	GVHD I-II	TT	TC	CC	Additive	0.647
			Yes	5	3	0	Dominant	0.647
				29.4%	42.9%	0.0%	Recessive	NA
			No	12	4	0	Homozygous	NA
				70.6%	57.1%	0.0%	Heterozygous	0.647
**D**	rs111394117	NOTCH4	GVHD I-II	GG	GA	AA	Additive	0.562
			Yes	8	0	0	Dominant	0.562
				38.1%	0.0%	0.0%	Recessive	NA
			No	13	3	0	Homozygous	NA
				61.9%	100.0%	0.0%	Heterozygous	0.562
**D**	rs9281491	HCP5	GVHD I-II	ins	insA	AA	Additive	0.423
			Yes	7	1	0	Dominant	0.352
				41.2%	16.7%	0.0%	Recessive	1.000
			No	10	5	1	Homozygous	1.000
				58.8%	83.3%	100.0%	Heterozygous	0.369
**D**	rs2244546	HCP5	GVHD I-II	CC	CG	GG	Additive	0.189
			Yes	7	1	0	Dominant	0.189
				43.8%	12.5%	0.0%	Recessive	NA
			No	9	7	0	Homozygous	NA
				56.3%	87.5%	0.0%	Heterozygous	0.189
**D**	rs4713466	HCP5	GVHD I-II	CC	CT	TT	Additive	0.390
			Yes	3	5	0	Dominant	0.390
				23.1%	45.5%	0.0%	Recessive	NA
			No	10	6	0	Homozygous	NA
				76.9%	54.5%	0.0%	Heterozygous	0.390
**D**	rs2523676	HCP5	GVHD I-II	CC	CT	TT	Additive	0.192
			Yes	2	6	0	Dominant	0.388
				20.0%	50.0%	0.0%	Recessive	0.536
			No	8	6	2	Homozygous	1.000
				80.0%	50.0%	100.0%	Heterozygous	0.204
**D**	rs2523675	HCP5	GVHD I-II	GG	GA	AA	Additive	0.592
			Yes	1	6	1	Dominant	0.621
				16.7%	40.0%	33.3%	Recessive	1.000
			No	5	9	2	Homozygous	1.000

				83.3%	60.0%	66.7%	Heterozygous	0.613
**D**	rs2518028	HCP5	GVHD I-II	CC	TC	TT	Additive	0.631
			Yes	7	1	0	Dominant	0.631
				36.8%	20.0%	0.0%	Recessive	NA
			No	12	4	0	Homozygous	NA
				63.2%	80.0%	0.0%	Heterozygous	0.631
**D**	rs141431529	HCP5	GVHD I-II	GG	GT	TT	Additive	0.262
			Yes	8	0	0	Dominant	0.262
				40.0%	0.0%	0.0%	Recessive	NA
			No	12	4	0	Homozygous	NA
				60.0%	100.0%	0.0%	Heterozygous	0.262
**D**	rs107822	RING1	GVHD I-II	TT	CT	CC	Additive	0.144
			Yes	3	5	0	Dominant	0.667
				25.0%	55.6%	0.0%	Recessive	0.526
			No	9	4	3	Homozygous	1.000
				75.0%	44.4%	100.0%	Heterozygous	0.203
**D**	rs213210	RING1	GVHD I-II	GG	AG	AA	Additive	**0.045**
			Yes	2	6	0	Dominant	0.657
				22.2%	60.0%	0.0%	Recessive	0.130
			No	7	4	5	Homozygous	0.505
				77.8%	40.0%	100.0%	Heterozygous	0.170
**D**	rs209132	TRIM27	GVHD I-II	GG	AG	AA	Additive	0.385
			Yes	3	5	0	Dominant	0.679
				27.3%	45.5%	0.0%	Recessive	0.536
			No	8	6	2	Homozygous	1.000
				72.7%	54.5%	100.0%	Heterozygous	0.659
**D**	rs209131	TRIM27	GVHD I-II	GG	AG	AA	Additive	0.687
			Yes	2	4	2	Dominant	0.667
				25.0%	33.3%	50.0%	Recessive	0.578
			No	6	8	2	Homozygous	0.547
				75.0%	66.7%	50.0%	Heterozygous	1.000
**D**	rs209130	TRIM27	GVHD I-II	TT	CT	CC	Additive	0.761
			Yes	4	4	0	Dominant	1.000
				33.3%	36.4%	0.0%	Recessive	1.000
			No	8	7	1	Homozygous	1.000
				66.7%	63.6%	100.0%	Heterozygous	1.000
**D**	rs1536215	TRIM27	GVHD I-II	CC	CG	GG	Additive	0.647
			Yes	5	3	0	Dominant	0.647
				29.4%	42.9%	0.0%	Recessive	NA
			No	12	4	0	Homozygous	NA
				70.6%	57.1%	0.0%	Heterozygous	0.647
**D**	rs139791445	TRIM27	GVHD I-II	CC	CG	GG	Additive	1.000
			Yes	8	0	0	Dominant	1.000
				34.8%	0.0%	0.0%	Recessive	NA
			No	15	1	0	Homozygous	NA
				65.2%	100.0%	0.0%	Heterozygous	1.000
**D**	rs11244	HLA-DOB	GVHD I-II	GG	AG	AA	Additive	0.320
			Yes	3	5	0	Dominant	1.000
				30.0%	45.5%	0.0%	Recessive	0.526

			No	7	6	3	Homozygous	0.528
				70.0%	54.5%	100.0%	Heterozygous	0.659
**D**	rs2070120	HLA-DOB	GVHD I-II	GG	AG	AA	Additive	1.000
			Yes	8	0	0	Dominant	1.000
				34.8%	0.0%	0.0%	Recessive	NA
			No	15	1	0	Homozygous	NA
				65.2%	100.0%	0.0%	Heterozygous	1.000
**D**	rs56150445	HLA-DOB	GVHD I-II	GG	CG	CC	Additive	NA
			Yes	8	0	0	Dominant	NA
				33.3%	0.0%	0.0%	Recessive	NA
			No	16	0	0	Homozygous	NA
				66.7%	0.0%	0.0%	Heterozygous	NA
**D**	rs41258084	HLA-DOB	GVHD I-II	CC	CT	TT	Additive	1.000
			Yes	7	1	0	Dominant	1.000
				31.8%	50.0%	0.0%	Recessive	NA
			No	15	1	0	Homozygous	NA
				68.2%	50.0%	0.0%	Heterozygous	1.000
**D**	rs17220087	HLA-DOB	GVHD I-II	CC	AC	AA	Additive	NA
			Yes	8	0	0	Dominant	NA
				34.8%	0.0%	0.0%	Recessive	NA
			No	15	1	0	Homozygous	NA
				65.2%	100.0%	0.0%	Heterozygous	NA
**D**	rs2071479	HLA-DOB	GVHD I-II	CC	CT	TT	Additive	1.000
			Yes	8	0	0	Dominant	1.000
				34.8%	0.0%	0.0%	Recessive	NA
			No	15	1	0	Homozygous	NA
				65.2%	100.0%	0.0%	Heterozygous	1.000
**D**	rs17213693	HLA-DOB	GVHD I-II	GG	CG	CC	Additive	1.000
			Yes	8	0	0	Dominant	1.000
				34.8%	0.0%	0.0%	Recessive	NA
			No	15	1	0	Homozygous	NA
				65.2%	100.0%	0.0%	Heterozygous	1.000
**D**	rs3130048	BAG6	GVHD I-II	TT	CT	CC	Additive	0.725
			Yes	4	2	2	Dominant	0.673
				28.6%	33.3%	50.0%	Recessive	0.578
			No	10	4	2	Homozygous	0.569
				71.4%	66.7%	50.0%	Heterozygous	1.000
**D**	rs2844464	BAG6	GVHD I-II	GG	AG	AA	Additive	0.631
			Yes	7	1	0	Dominant	0.631
				36.8%	20.0%	0.0%	Recessive	NA
			No	12	4	0	Homozygous	NA
				63.2%	80.0%	0.0%	Heterozygous	0.631
**D**	rs2242656	BAG6	GVHD I-II	TT	CT	CC	Additive	0.631
			Yes	7	1	0	Dominant	0.631
				36.8%	20.0%	0.0%	Recessive	NA
			No	12	4	0	Homozygous	NA
				63.2%	80.0%	0.0%	Heterozygous	0.631
**M**	rs107822	RING1	GVHD I-II		matched		unmatched	
			Yes		3		5	0.667

				27.3%	41.7%	
			No	8	7	
				72.7%	58.3%	
**M**	rs213210	RING1	GVHD I-II	matched	unmatched	
			Yes	2	6	1.000
				28.6%	37.5%	
			No	5	10	
				71.4%	62.5%	
**M**	rs209132	TRIM27	GVHD I-II	matched	unmatched	
			Yes	4	4	0.412
				26.7%	44.4%	
			No	11	5	
				73.3%	55.6%	
**M**	rs209131	TRIM27	GVHD I-II	matched	unmatched	
			Yes	4	4	0.673
				28.6%	40.0%	
			No	10	6	
				71.4%	60.0%	
**M**	rs209130	TRIM27	GVHD I-II	matched	unmatched	
			Yes	3	5	0.204
				21.4%	50.0%	
			No	11	5	
				78.6%	50.0%	
**M**	rs1536215	TRIM27	GVHD I-II	matched	unmatched	
			Yes	6	2	0.578
				30.0%	50.0%	
			No	14	2	
				70.0%	50.0%	
**M**	rs13979144	TRIM27	GVHD I-II	matched	unmatched	
			Yes	8	0	1.000
				34.8%	0.0%	
			No	15	1	
				65.2%	100.0%	
**M**	rs11244	HLA-DOB	GVHD I-II	matched	unmatched	
			Yes	4	4	0.685
				30.8%	40.0%	
			No	9	6	
				69.2%	60.0%	
**M**	rs2070120	HLA-DOB	GVHD I-II	matched	unmatched	
			Yes	7	1	0.348
				31.8%	100.0%	
			No	15	0	
				68.2%	0.0%	
**M**	rs56150445	HLA-DOB	GVHD I-II	matched	unmatched	
			Yes	8	0	NA
				34.8%	0.0%	
			No	15	0	
				65.2%	0.0%	
**M**	rs41258084	HLA-DOB	GVHD I-II	matched	unmatched	

			Yes	8	0	NA
				34.8%	0.0%	
			No	15	0	
				65.2%	0.0%	
**M**	rs17220087	HLA-DOB	GVHD I-II	matched	unmatched	
			Yes	7	1	1.000
				33.3%	50.0%	
			No	14	1	
				66.7%	50.0%	
**M**	rs2071479	HLA-DOB	GVHD I-II	matched	unmatched	
			Yes	8	0	1.000
				36.4%	0.0%	
			No	14	1	
				63.6%	100.0%	
**M**	rs17213693	HLA-DOB	GVHD I-II	matched	unmatched	
			Yes	7	1	0.348
				31.8%	100.0%	
			No	15	0	
				68.2%	0.0%	
**M**	rs3130048	BAG6	GVHD I-II	matched	unmatched	
			Yes	4	4	1.000
				30.8%	36.4%	
			No	9	7	
				69.2%	63.6%	
**M**	rs2844464	BAG6	GVHD I-II	matched	unmatched	
			Yes	5	3	0.647
				29.4%	42.9%	
			No	12	4	
				70.6%	57.1%	
**M**	rs2242656	BAG6	GVHD I-II	matched	unmatched	
			Yes	5	3	0.362
				27.8%	50.0%	
			No	13	3	
				72.2%	50.0%	

**Source of DNA[a]**	**SNP**	**Gene**	**Outcome/ status**	**Number of patients**			**Model**	***p*-value**
**D**	rs11571315	CTLA4	GVHD I-II	TT	CT	CC	Additive	0.703
			Yes	5	3	0	Dominant	0.679
				38.5%	30.0%	0.0%	Recessive	1.000
			No	8	7	1	Homozygous	1.000
				61.5%	70.0%	100.0%	Heterozygous	1.000
**D**	rs733618	CTLA4	GVHD I-II	CC	CT	TT	Additive	0.276
			Yes	2	6	0	Dominant	1.000
				33.3%	42.9%	0.0%	Recessive	0.262
			No	4	8	4	Homozygous	1.000
				66.7%	57.1%	100.0%	Heterozygous	1.000
**D**	rs4553808	CTLA4	GVHD I-II	AA	AG	GG	Additive	1.000
			Yes	7	1	0	Dominant	1.000
				33.3%	33.3%	0.0%	Recessive	NA
			No	14	2	0	Homozygous	NA
				66.7%	66.7%	0.0%	Heterozygous	1.000

**D**	rs11571316	CTLA4	GVHD I-II	GG	AG	AA	Additive	0.388
			Yes	6	2	0	Dominant	0.388
				42.9%	20.0%	0.0%	Recessive	NA
			No	8	8	0	Homozygous	NA
				57.1%	80.0%	0.0%	Heterozygous	0.388
**D**	rs62182595	CTLA4	GVHD I-II	GG	AG	AA	Additive	1.000
			Yes	7	1	0	Dominant	1.000
				33.3%	33.3%	0.0%	Recessive	NA
			No	14	2	0	Homozygous	NA
				66.7%	66.7%	0.0%	Heterozygous	1.000
**D**	rs573554201	CTLA4	GVHD I-II	AA	AG	GG	Additive	NA
			Yes	8	0	0	Dominant	NA
				33.3%	0.0%	0.0%	Recessive	NA
			No	16	0	0	Homozygous	NA
				66.7%	0.0%	0.0%	Heterozygous	NA
**D**	rs16840252	CTLA4	GVHD I-II	CC	CT	TT	Additive	1.000
			Yes	7	1	0	Dominant	1.000
				33.3%	33.3%	0.0%	Recessive	NA
			No	14	2	0	Homozygous	NA
				66.7%	66.7%	0.0%	Heterozygous	1.000
**D**	rs945677329	CTLA4	GVHD I-II	AA	AG	GG	Additive	0.333
			Yes	7	1	0	Dominant	0.333
				30.4%	100.0%	0.0%	Recessive	NA
			No	16	0	0	Homozygous	NA
				69.6%	0.0%	0.0%	Heterozygous	0.333
**D**	rs5742909	CTLA4	GVHD I-II	CC	CT	TT	Additive	0.631
			Yes	7	1	0	Dominant	0.631
				36.8%	20.0%	0.0%	Recessive	NA
			No	12	4	0	Homozygous	NA
				63.2%	80.0%	0.0%	Heterozygous	0.631
**D**	rs231775	CTLA4	GVHD I-II	GG	AG	AA	Additive	0.449
			Yes	5	3	0	Dominant	0.390
				45.5%	25.0%	0.0%	Recessive	1.000
			No	6	9	1	Homozygous	1.000
				54.5%	75.0%	100.0%	Heterozygous	0.400
**D**	rs56102377	CTLA4	GVHD I-II	GG	AG	AA	Additive	1.000
			Yes	7	1	0	Dominant	1.000
				31.8%	50.0%	0.0%	Recessive	NA
			No	15	1	0	Homozygous	NA
				68.2%	50.0%	0.0%	Heterozygous	1.000
**D**	rs56217811	CTLA4	GVHD I-II	CC	CA	AA	Additive	NA
			Yes	8	0	0	Dominant	NA
				33.3%	0.0%	0.0%	Recessive	NA
			No	16	0	0	Homozygous	NA
				66.7%	0.0%	0.0%	Heterozygous	NA
**D**	rs55696217	CTLA4	GVHD I-II	GG	GA	AA	Additive	NA
			Yes	8	0	0	Dominant	NA
				33.3%	0.0%	0.0%	Recessive	NA
			No	16	0	0	Homozygous	NA

				66.7%	0.0%	0.0%	Heterozygous	NA
**D**	rs231721	CTLA4	GVHD I-II	TT	CT	CC	Additive	NA
			Yes	8	0	0	Dominant	NA
				33.3%	0.0%	0.0%	Recessive	NA
			No	16	0	0	Homozygous	NA
				66.7%	0.0%	0.0%	Heterozygous	NA
**D**	rs778932058	CTLA4	GVHD I-II	AA	AG	GG	Additive	NA
			Yes	8	0	0	Dominant	NA
				33.3%	0.0%	0.0%	Recessive	NA
			No	16	0	0	Homozygous	NA
				66.7%	0.0%	0.0%	Heterozygous	NA
**D**	rs3087243	CTLA4	GVHD I-II	GG	AG	AA	Additive	0.679
			Yes	5	3	0	Dominant	0.679
				38.5%	27.3%	0.0%	Recessive	NA
			No	8	8	0	Homozygous	NA
				61.5%	72.7%	0.0%	Heterozygous	0.679
**D**	rs11571319	CTLA4	GVHD I-II	GG	AG	AA	Additive	1.000
			Yes	7	1	0	Dominant	1.000
				35.0%	25.0%	0.0%	Recessive	NA
			No	13	3	0	Homozygous	NA
				65.0%	75.0%	0.0%	Heterozygous	1.000
**D**	rs1234314	TNFSF4	GVHD I-II	CC	CG	GG	Additive	0.247
			Yes	3	2	3	Dominant	1.000
				37.5%	18.2%	60.0%	Recessive	0.289
			No	5	9	2	Homozygous	0.592
				62.5%	81.8%	40.0%	Heterozygous	0.603
**D**	rs45454293	TNFSF4	GVHD I-II	CC	CT	TT	Additive	0.658
			Yes	4	3	1	Dominant	0.412
				26.7%	42.9%	50.0%	Recessive	1.000
			No	11	4	1	Homozygous	0.515
				73.3%	57.1%	50.0%	Heterozygous	0.630
**D**	rs181758110	TNFSF4	GVHD I-II	CC	CT	TT	Additive	NA
			Yes	8	0	0	Dominant	NA
				33.3%	0.0%	0.0%	Recessive	NA
			No	16	0	0	Homozygous	NA
				66.7%	0.0%	0.0%	Heterozygous	NA
**D**	rs1879877	CD28	GVHD I-II	TT	GT	GG	Additive	0.723
			Yes	3	4	0	Dominant	0.657
				37.5%	28.6%	0.0%	Recessive	1.000
			No	5	10	1	Homozygous	1.000
				62.5%	71.4%	100.0%	Heterozygous	0.182
**D**	rs3181096	CD28	GVHD I-II	CC	CT	TT	Additive	0.657
			Yes	5	2	0	Dominant	0.657
				35.7%	22.2%	0.0%	Recessive	NA
			No	9	7	0	Homozygous	NA
				64.3%	77.8%	0.0%	Heterozygous	0.657
**D**	rs3181097	CD28	GVHD I-II	AA	AG	GG	Additive	0.298
			Yes	3	4	0	Dominant	0.318
				50.0%	28.6%	0.0%	Recessive	0.526

			No	3	10	3	Homozygous	0.464
				50.0%	71.4%	100.0%	Heterozygous	0.613
**D**	rs3181098	CD28	GVHD I-II	GG	AG	AA	Additive	0.657
			Yes	5	2	0	Dominant	0.657
				35.7%	22.2%	0.0%	Recessive	NA
			No	9	7	0	Homozygous	NA
				64.3%	77.8%	0.0%	Heterozygous	0.657
**D**	rs28718975	CD28	GVHD I-II	TT	CT	CC	Additive	0.792
			Yes	5	2	0	Dominant	1.000
				31.3%	33.3%	0.0%	Recessive	1.000
			No	11	4	1	Homozygous	1.000
				68.8%	66.7%	100.0%	Heterozygous	1.000
**D**	rs28688913	CD28	GVHD I-II	CC	CT	TT	Additive	1.000
			Yes	5	2	0	Dominant	1.000
				31.3%	28.6%	0.0%	Recessive	NA
			No	11	5	0	Homozygous	NA
				68.8%	71.4%	0.0%	Heterozygous	1.000
**D**	rs28541784	CD28	GVHD I-II	CC	CT	TT	Additive	1.000
			Yes	5	2	0	Dominant	1.000
				29.4%	33.3%	0.0%	Recessive	NA
			No	12	4	0	Homozygous	NA
				70.6%	66.7%	0.0%	Heterozygous	1.000
**D**	rs201801072	CD28	GVHD I-II	TT	CT	CC	Additive	1.000
			Yes	5	2	0	Dominant	1.000
				31.3%	28.6%	0.0%	Recessive	NA
			No	11	5	0	Homozygous	NA
				68.8%	71.4%	0.0%	Heterozygous	1.000
**D**	rs200353921	CD28	GVHD I-II	TT	AT	AA	Additive	1.000
			Yes	5	2	0	Dominant	1.000
				31.3%	28.6%	0.0%	Recessive	NA
			No	11	5	0	Homozygous	NA
				68.8%	71.4%	0.0%	Heterozygous	1.000
**D**	rs56228674	CD28	GVHD I-II	CC	CT	TT	Additive	1.000
			Yes	7	0	0	Dominant	1.000
				33.3%	0.0%	0.0%	Recessive	NA
			No	14	2	0	Homozygous	NA
				66.7%	100.0%	0.0%	Heterozygous	1.000
**D**	rs1290180288	CD28	GVHD I-II	TT	CT	CC	Additive	NA
			Yes	7	0	0	Dominant	NA
				30.4%	0.0%	0.0%	Recessive	NA
			No	16	0	0	Homozygous	NA
				69.6%	0.0%	0.0%	Heterozygous	NA
**D**	rs3116496	CD28	GVHD I-II	TT	CT	CC	Additive	1.000
			Yes	7	0	0	Dominant	1.000
				33.3%	0.0%	0.0%	Recessive	NA
			No	14	2	0	Homozygous	NA
				66.7%	100.0%	0.0%	Heterozygous	1.000
**D**	rs375758971	CD28	GVHD I-II	TT	CT	CC	Additive	NA
			Yes	7	0	0	Dominant	NA

				30.4%	0.0%	0.0%	Recessive	NA
			No	16	0	0	Homozygous	NA
				69.6%	0.0%	0.0%	Heterozygous	NA
**D**	rs36084323	PD1	GVHD I-II	CC	CT	TT	Additive	0.127
			Yes	1	6	1	Dominant	0.352
				14.3%	54.5%	16.7%	Recessive	0.621
			No	6	5	5	Homozygous	1.000
				85.7%	45.5%	83.3%	Heterozygous	0.151
**D**	rs5839828	PD1	GVHD I-II	del	delG	GG	Additive	0.218
			Yes	1	6	1	Dominant	0.352
				14.3%	50.0%	20.0%	Recessive	0.631
			No	6	6	4	Homozygous	1.000
				85.7%	50.0%	80.0%	Heterozygous	0.173
**D**	rs11568821	PD1	GVHD I-II	CC	CA	AA	Additive	NA
			Yes	8	0	0	Dominant	NA
				33.3%	0.0%	0.0%	Recessive	NA
			No	16	0	0	Homozygous	NA
				66.7%	0.0%	0.0%	Heterozygous	NA
**D**	rs41386349	PD1	GVHD I-II	GG	AG	AA	Additive	**0.050**
			Yes	8	0	0	Dominant	**0.022**
				50.0%	0.0%	0.0%	Recessive	1.000
			No	8	7	1	Homozygous	1.000
				50.0%	100.0%	100.0%	Heterozygous	0.052
**D**	rs6705653	PD1	GVHD I-II	CC	CT	TT	Additive	0.590
			Yes	5	3	0	Dominant	0.667
				41.7%	27.3%	0.0%	Recessive	1.000
			No	7	8	1	Homozygous	0.667
				58.3%	72.7%	100.0%	Heterozygous	0.667
**D**	rs2227982	PD1	GVHD I-II	GG	AG	AA	Additive	0.064
			Yes	1	6	1	Dominant	0.352
				14.3%	60.0%	14.3%	Recessive	0.352
			No	6	4	6	Homozygous	1.000
				85.7%	40.0%	85.7%	Heterozygous	0.134

[a]P: patient; D: donor; M: patient and donor mismatch status.
