# Supplementary material for: Preliminary Data on SNP of Transplantation-Related Genes after Haploidentical Stem Cell Transplantation
Source: J Clin Med. 2024 Aug 9;13(16):4681. doi: 10.3390/jcm13164681 (PMC11354871; doi:10.3390/jcm13164681)
Supplement: Supplementary file 1 [file jcm-13-04681-s001.zip › Supplementary Table 6_20240718.pdf]

Table S6. Association analysis between SNPs and GVHD III-IV status post-haplo-HSCT.

Source of DNA[a]	SNP	Gene	Outcome/ status	Number of patients			Model	*p*-value
**P**	rs9276982	HLA-DOA	GVHD III-IV	GG	AG	AA	Additive	0.362
			Yes	3	3	0	Dominant	0.362
				18.8%	37.5%	0.0%	Recessive	NA
			No	13	5	0	Homozygous	NA
				81.3%	62.5%	0.0%	Heterozygous	0.362
**P**	rs71565361	HLA-DOA	GVHD III-IV	CC	CA	AA	Additive	1.000
			Yes	6	0	0	Dominant	1.000
				26.1%	0.0%	0.0%	Recessive	NA
			No	17	1	0	Homozygous	NA
				73.9%	100.0%	0.0%	Heterozygous	1.000
**P**	rs79327197	HLA-DOA	GVHD III-IV	AA	AG	GG	Additive	0.251
			Yes	4	2	0	Dominant	0.251
				20.0%	50.0%	0.0%	Recessive	NA
			No	16	2	0	Homozygous	NA
				80.0%	50.0%	0.0%	Heterozygous	0.251
**P**	rs151190962	HLA-DOA	GVHD III-IV	AA	delA	del	Additive	0.250
			Yes	5	1	0	Dominant	0.250
				21.7%	100.0%	0.0%	Recessive	NA
			No	18	0	0	Homozygous	NA
				78.3%	0.0%	0.0%	Heterozygous	0.250
**P**	rs9282369	HLA-DOA	GVHD III-IV	ins	insT	TT	Additive	0.146
			Yes	2	4	0	Dominant	1.000
				22.2%	44.4%	0.0%	Recessive	0.277
			No	7	5	6	Homozygous	0.486
				77.8%	55.6%	100.0%	Heterozygous	0.620
**P**	rs2009658	LTA	GVHD III-IV	CC	CG	GG	Additive	1.000
			Yes	4	2	0	Dominant	1.000
				25.0%	25.0%	0.0%	Recessive	NA
			No	12	6	0	Homozygous	NA
				75.0%	75.0%	0.0%	Heterozygous	1.000
**P**	rs111250247	LTA	GVHD III-IV	TT	TA	AA	Additive	NA
			Yes	6	0	0	Dominant	NA
				25.0%	0.0%	0.0%	Recessive	NA
			No	18	0	0	Homozygous	NA
				75.0%	0.0%	0.0%	Heterozygous	NA
**P**	rs915654	LTA	GVHD III-IV	AA	TA	TT	Additive	0.915
			Yes	1	4	1	Dominant	1.000
				20.0%	25.0%	33.3%	Recessive	1.000
			No	4	12	2	Homozygous	1.000
				80.0%	75.0%	66.7%	Heterozygous	1.000
**P**	rs34324979	HSPA1L	GVHD III-IV	GG	Gins	ins	Additive	0.618
			Yes	4	2	0	Dominant	0.618
				22.2%	33.3%	0.0%	Recessive	NA
			No	14	4	0	Homozygous	NA
				77.8%	66.7%	0.0%	Heterozygous	0.618

**P**	rs2075800	HSPA1L	GVHD III-IV	CC	CT	TT	Additive	
			Yes	1	5	0	Dominant	
				16.7%	35.7%	0.0%	Recessive	0.539
			No	5	9	4	Homozygous	
				83.3%	64.3%	100.0%	Heterozygous	
**P**	rs2227956	HSPA1L	GVHD III-IV	AA	GA	GG	Additive	0.251
			Yes	4	2	0	Dominant	0.251
				20.0%	50.0%	0.0%	Recessive	NA
			No	16	2	0	Homozygous	NA
				80.0%	50.0%	0.0%	Heterozygous	0.251
**P**	rs107822	RING1	GVHD III-IV	TT	CT	CC	Additive	0.224
			Yes	3	2	1	Dominant	0.643
				21.4%	25.0%	100.0%	Recessive	0.261
			No	11	6	0	Homozygous	0.267
				78.6%	75.0%	0.0%	Heterozygous	1.000
**P**	rs213210	RING1	GVHD III-IV	GG	AG	AA	Additive	0.259
			Yes	2	4	0	Dominant	0.341
				15.4%	44.4%	0.0%	Recessive	1.000
			No	11	5	1	Homozygous	1.000
				84.6%	55.6%	100.0%	Heterozygous	0.178
**P**	rs209132	TRIM27	GVHD III-IV	GG	AG	AA	Additive	0.934
			Yes	3	2	1	Dominant	1.000
				23.1%	25.0%	33.3%	Recessive	1.000
			No	10	6	2	Homozygous	1.000
				76.9%	75.0%	66.7%	Heterozygous	1.000
**P**	rs209131	TRIM27	GVHD III-IV	GG	AG	AA	Additive	0.953
			Yes	2	3	1	Dominant	1.000
				25.0%	27.3%	20.0%	Recessive	1.000
			No	6	8	4	Homozygous	1.000
				75.0%	72.7%	80.0%	Heterozygous	1.000
**P**	rs209130	TRIM27	GVHD III-IV	TT	CT	CC	Additive	0.599
			Yes	3	3	0	Dominant	1.000
				23.1%	33.3%	0.0%	Recessive	1.000
			No	10	6	2	Homozygous	1.000
				76.9%	66.7%	100.0%	Heterozygous	0.655
**P**	rs1536215	TRIM27	GVHD III-IV	CC	CG	GG	Additive	1.000
			Yes	4	2	0	Dominant	1.000
				23.5%	28.6%	0.0%	Recessive	NA
			No	13	5	0	Homozygous	NA
				76.5%	71.4%	0.0%	Heterozygous	1.000
**P**	rs139791445	TRIM27	GVHD III-IV	CC	CG	GG	Additive	NA
			Yes	6	0	0	Dominant	NA
				25.0%	0.0%	0.0%	Recessive	NA
			No	18	0	0	Homozygous	NA
				75.0%	0.0%	0.0%	Heterozygous	NA
**P**	rs11244	HLA-DOB	GVHD III-IV	GG	AG	AA	Additive	0.558
			Yes	4	1	0	Dominant	0.611
				28.6%	14.3%	0.0%	Recessive	1.000
			No	10	6	2	Homozygous	1.000

				71.4%	85.7%	100.0%	Heterozygous	0.624
**P**	rs2070120	HLA-DOB	GVHD III-IV	GG	AG	AA	Additive	0.539
			Yes	4	1	0	Dominant	0.539
				20.0%	33.3%	0.0%	Recessive	NA
			No	16	2	0	Homozygous	NA
				80.0%	66.7%	0.0%	Heterozygous	0.539
**P**	rs56150445	HLA-DOB	GVHD III-IV	GG	CG	CC	Additive	NA
			Yes	5	0	0	Dominant	NA
				21.7%	0.0%	0.0%	Recessive	NA
			No	18	0	0	Homozygous	NA
				78.3%	0.0%	0.0%	Heterozygous	NA
**P**	rs41258084	HLA-DOB	GVHD III-IV	CC	CT	TT	Additive	1.000
			Yes	5	0	0	Dominant	1.000
				23.8%	0.0%	0.0%	Recessive	NA
			No	16	2	0	Homozygous	NA
				76.2%	100.0%	0.0%	Heterozygous	1.000
**P**	rs17220087	HLA-DOB	GVHD III-IV	CC	AC	AA	Additive	0.539
			Yes	4	1	0	Dominant	0.539
				20.0%	33.3%	0.0%	Recessive	NA
			No	16	2	0	Homozygous	NA
				80.0%	66.7%	0.0%	Heterozygous	0.539
**P**	rs2071479	HLA-DOB	GVHD III-IV	CC	CT	TT	Additive	1.000
			Yes	5	0	0	Dominant	1.000
				22.7%	0.0%	0.0%	Recessive	NA
			No	17	1	0	Homozygous	NA
				77.3%	100.0%	0.0%	Heterozygous	1.000
**P**	rs17213693	HLA-DOB	GVHD III-IV	GG	CG	CC	Additive	1.000
			Yes	4	1	0	Dominant	1.000
				21.1%	25.0%	0.0%	Recessive	NA
			No	15	3	0	Homozygous	NA
				78.9%	75.0%	0.0%	Heterozygous	1.000
**P**	rs3130048	BAG6	GVHD III-IV	TT	CT	CC	Additive	0.223
			Yes	2	4	0	Dominant	0.192
				14.3%	44.4%	0.0%	Recessive	1.000
			No	12	5	1	Homozygous	1.000
				85.7%	55.6%	100.0%	Heterozygous	0.162
**P**	rs2844464	BAG6	GVHD III-IV	GG	AG	AA	Additive	0.789
			Yes	5	1	0	Dominant	1.000
				27.8%	20.0%	0.0%	Recessive	1.000
			No	13	4	1	Homozygous	1.000
				72.2%	80.0%	100.0%	Heterozygous	1.000
**P**	rs2242656	BAG6	GVHD III-IV	TT	CT	CC	Additive	1.000
			Yes	5	1	0	Dominant	1.000
				26.3%	20.0%	0.0%	Recessive	NA
			No	14	4	0	Homozygous	NA
				73.7%	80.0%	0.0%	Heterozygous	1.000
**D**	rs61365987	NOTCH4	GVHD III-IV	CC	CT	TT	Additive	1.000
			Yes	5	1	0	Dominant	1.000

				23.8%	33.3%	0.0%	Recessive	NA
			No	16	2	0	Homozygous	NA
				76.2%	66.7%	0.0%	Heterozygous	1.000
**D**	rs444472	NOTCH4	GVHD III-IV	GG	GA	AA	Additive	0.180
			Yes	4	1	1	Dominant	0.250
				25.0%	14.3%	100.0%	Recessive	0.446
			No	12	6	0	Homozygous	0.294
				75.0%	85.7%	0.0%	Heterozygous	1.000
**D**	rs2256594	NOTCH4	GVHD III-IV	AA	AG	GG	Additive	0.409
			Yes	1	3	2	Dominant	0.629
				14.3%	23.1%	50.0%	Recessive	0.251
			No	6	10	2	Homozygous	0.491
				85.7%	76.9%	50.0%	Heterozygous	1.000
**D**	rs394657	NOTCH4	GVHD III-IV	AA	AG	GG	Additive	0.180
			Yes	4	1	1	Dominant	1.000
				25.0%	14.3%	100.0%	Recessive	0.250
			No	12	6	0	Homozygous	0.294
				75.0%	85.7%	0.0%	Heterozygous	1.000
**D**	rs429853	NOTCH4	GVHD III-IV	TT	TC	CC	Additive	1.000
			Yes	4	2	0	Dominant	1.000
				23.5%	28.6%	0.0%	Recessive	NA
			No	13	5	0	Homozygous	NA
				76.5%	71.4%	0.0%	Heterozygous	1.000
**D**	rs111394117	NOTCH4	GVHD III-IV	GG	GA	AA	Additive	1.000
			Yes	5	1	0	Dominant	1.000
				23.8%	33.3%	0.0%	Recessive	NA
			No	16	2	0	Homozygous	NA
				76.2%	66.7%	0.0%	Heterozygous	1.000
**D**	rs9281491	HCP5	GVHD III-IV	ins	insA	AA	Additive	0.750
			Yes	4	2	0	Dominant	1.000
				23.5%	33.3%	0.0%	Recessive	1.000
			No	13	4	1	Homozygous	1.000
				76.5%	66.7%	100.0%	Heterozygous	0.632
**D**	rs2244546	HCP5	GVHD III-IV	CC	CG	GG	Additive	0.129
			Yes	2	4	0	Dominant	0.129
				12.5%	50.0%	0.0%	Recessive	NA
			No	14	4	0	Homozygous	NA
				87.5%	50.0%	0.0%	Heterozygous	0.129
**D**	rs4713466	HCP5	GVHD III-IV	CC	CT	TT	Additive	1.000
			Yes	3	3	0	Dominant	1.000
				23.1%	27.3%	0.0%	Recessive	NA
			No	10	8	0	Homozygous	NA
				76.9%	72.7%	0.0%	Heterozygous	1.000
**D**	rs2523676	HCP5	GVHD III-IV	CC	CT	TT	Additive	0.537
			Yes	2	4	0	Dominant	1.000
				20.0%	33.3%	0.0%	Recessive	1.000
			No	8	8	2	Homozygous	1.000
				80.0%	66.7%	100.0%	Heterozygous	0.646

**D**	rs2523675	HCP5	GVHD III-IV	GG	GA	AA	Additive	0.537
			Yes	2	4	0	Dominant	0.618
				33.3%	26.7%	0.0%	Recessive	0.546
			No	4	11	3	Homozygous	0.500
				66.7%	73.3%	100.0%	Heterozygous	1.000
**D**	rs2518028	HCP5	GVHD III-IV	CC	TC	TT	Additive	1.000
			Yes	5	1	0	Dominant	1.000
				26.3%	20.0%	0.0%	Recessive	NA
			No	14	4	0	Homozygous	NA
				73.7%	80.0%	0.0%	Heterozygous	1.000
**D**	rs141431529	HCP5	GVHD III-IV	GG	GT	TT	Additive	0.251
			Yes	4	2	0	Dominant	0.251
				20.0%	50.0%	0.0%	Recessive	NA
			No	16	2	0	Homozygous	NA
				80.0%	50.0%	0.0%	Heterozygous	0.251
**D**	rs107822	RING1	GVHD III-IV	TT	CT	CC	Additive	0.157
			Yes	3	1	2	Dominant	1.000
				25.0%	11.1%	66.7%	Recessive	0.143
			No	9	8	1	Homozygous	0.242
				75.0%	88.9%	33.3%	Heterozygous	0.603
**D**	rs213210	RING1	GVHD III-IV	GG	AG	AA	Additive	**0.031**
			Yes	3	0	3	Dominant	0.635
				33.3%	0.0%	60.0%	Recessive	0.078
			No	6	10	2	Homozygous	0.580
				66.7%	100.0%	40.0%	Heterozygous	0.087
**D**	rs209132	TRIM27	GVHD III-IV	GG	AG	AA	Additive	0.616
			Yes	3	2	1	Dominant	1.000
				27.3%	18.2%	50.0%	Recessive	0.446
			No	8	9	1	Homozygous	1.000
				72.7%	81.8%	50.0%	Heterozygous	1.000
**D**	rs209131	TRIM27	GVHD III-IV	GG	AG	AA	Additive	1.000
			Yes	2	3	1	Dominant	1.000
				25.0%	25.0%	25.0%	Recessive	1.000
			No	6	9	3	Homozygous	1.000
				75.0%	75.0%	75.0%	Heterozygous	1.000
**D**	rs209130	TRIM27	GVHD III-IV	TT	CT	CC	Additive	0.834
			Yes	3	3	0	Dominant	1.000
				25.0%	27.3%	0.0%	Recessive	1.000
			No	9	8	1	Homozygous	1.000
				75.0%	72.7%	100.0%	Heterozygous	1.000
**D**	rs1536215	TRIM27	GVHD III-IV	CC	CG	GG	Additive	1.000
			Yes	4	2	0	Dominant	1.000
				23.5%	28.6%	0.0%	Recessive	NA
			No	13	5	0	Homozygous	NA
				76.5%	71.4%	0.0%	Heterozygous	1.000
**D**	rs139791445	TRIM27	GVHD III-IV	CC	CG	GG	Additive	1.000
			Yes	6	0	0	Dominant	1.000
				26.1%	0.0%	0.0%	Recessive	NA
			No	17	1	0	Homozygous	NA

				73.9%	100.0%	0.0%	Heterozygous	1.000
**D**	rs11244	HLA-DOB	GVHD III-IV	GG	AG	AA	Additive	**0.032**
			Yes	0	4	2	Dominant	**0.024**
				0.0%	36.4%	66.7%	Recessive	0.143
			No	10	7	1	Homozygous	**0.038**
				100.0%	63.6%	33.3%	Heterozygous	0.090
**D**	rs2070120	HLA-DOB	GVHD III-IV	GG	AG	AA	Additive	1.000
			Yes	6	0	0	Dominant	1.000
				26.1%	0.0%	0.0%	Recessive	NA
			No	17	1	0	Homozygous	NA
				73.9%	100.0%	0.0%	Heterozygous	1.000
**D**	rs56150445	HLA-DOB	GVHD III-IV	GG	CG	CC	Additive	NA
			Yes	6	0	0	Dominant	NA
				25.0%	0.0%	0.0%	Recessive	NA
			No	18	0	0	Homozygous	NA
				75.0%	0.0%	0.0%	Heterozygous	NA
**D**	rs41258084	HLA-DOB	GVHD III-IV	CC	CT	TT	Additive	1.000
			Yes	6	0	0	Dominant	1.000
				27.3%	0.0%	0.0%	Recessive	NA
			No	16	2	0	Homozygous	NA
				72.7%	100.0%	0.0%	Heterozygous	1.000
**D**	rs17220087	HLA-DOB	GVHD III-IV	CC	AC	AA	Additive	NA
			Yes	6	0	0	Dominant	NA
				26.1%	0.0%	0.0%	Recessive	NA
			No	17	1	0	Homozygous	NA
				73.9%	100.0%	0.0%	Heterozygous	NA
**D**	rs2071479	HLA-DOB	GVHD III-IV	CC	CT	TT	Additive	0.250
			Yes	5	1	0	Dominant	0.250
				21.7%	100.0%	0.0%	Recessive	NA
			No	18	0	0	Homozygous	NA
				78.3%	0.0%	0.0%	Heterozygous	0.250
**D**	rs17213693	HLA-DOB	GVHD III-IV	GG	CG	CC	Additive	1.000
			Yes	6	0	0	Dominant	1.000
				26.1%	0.0%	0.0%	Recessive	NA
			No	17	1	0	Homozygous	NA
				73.9%	100.0%	0.0%	Heterozygous	1.000
**D**	rs3130048	BAG6	GVHD III-IV	TT	CT	CC	Additive	0.853
			Yes	4	1	1	Dominant	1.000
				28.6%	16.7%	25.0%	Recessive	1.000
			No	10	5	3	Homozygous	1.000
				71.4%	83.3%	75.0%	Heterozygous	1.000
**D**	rs2844464	BAG6	GVHD III-IV	GG	AG	AA	Additive	1.000
			Yes	5	1	0	Dominant	1.000
				26.3%	20.0%	0.0%	Recessive	NA
			No	14	4	0	Homozygous	NA
				73.7%	80.0%	0.0%	Heterozygous	1.000
**D**	rs2242656	BAG6	GVHD III-IV	TT	CT	CC	Additive	1.000
			Yes	5	1	0	Dominant	1.000

				26.3%	20.0%	0.0%	Recessive	NA
			No	14	4	0	Homozygous	NA
				73.7%	80.0%	0.0%	Heterozygous	1.000
M	rs107822	RING1	GVHD III-IV		matched		unmatched	
			Yes		3		3	1.000
					27.3%		25.0%	
			No		8		9	
					72.7%		75.0%	
**M**	rs213210	RING1	GVHD III-IV		matched		unmatched	
			Yes		1		5	0.621
					14.3%		31.3%	
			No		6		11	
					85.7%		68.8%	
**M**	rs209132	TRIM27	GVHD III-IV		matched		unmatched	
			Yes		4		2	1.000
					26.7%		22.2%	
			No		11		7	
					73.3%		77.8%	
**M**	rs209131	TRIM27	GVHD III-IV		matched		unmatched	
			Yes		3		3	0.665
					21.4%		30.0%	
			No		11		7	
					78.6%		70.0%	
**M**	rs209130	TRIM27	GVHD III-IV		matched		unmatched	
			Yes		4		2	1.000
					28.6%		20.0%	
			No		10		8	
					71.4%		80.0%	
**M**	rs1536215	TRIM27	GVHD III-IV		matched		unmatched	
			Yes		6		0	0.539
					30.0%		0.0%	
			No		14		4	
					70.0%		100.0%	
**M**	rs139791445	TRIM27	GVHD III-IV		matched		unmatched	
			Yes		6		0	1.000
					26.1%		0.0%	
			No		17		1	
					73.9%		100.0%	
**M**	rs11244	HLA-DOB	GVHD III-IV		matched		unmatched	
			Yes		0		5	**0.007**
					0.0%		50.0%	
			No		13		5	
					100.0%		50.0%	
**M**	rs2070120	HLA-DOB	GVHD III-IV		matched		unmatched	
			Yes		5		0	1.000
					22.7%		0.0%	
			No		17		1	
					77.3%		100.0%	
**M**	rs56150445	HLA-DOB	GVHD III-IV		matched		unmatched	

			Yes	5	0	NA
				21.7%	0.0%	
			No	18	0	
				78.3%	0.0%	
**M**	rs41258084	HLA-DOB	GVHD III-IV	matched	unmatched	
			Yes	5	0	NA
				21.7%	0.0%	
			No	18	0	
				78.3%	0.0%	
**M**	rs17220087	HLA-DOB	GVHD III-IV	matched	unmatched	
			Yes	4	1	0.395
				19.0%	50.0%	
			No	17	1	
				81.0%	50.0%	
**M**	rs2071479	HLA-DOB	GVHD III-IV	matched	unmatched	
			Yes	5	0	1.000
				22.7%	0.0%	
			No	17	1	
				77.3%	100.0%	
**M**	rs17213693	HLA-DOB	GVHD III-IV	matched	unmatched	
			Yes	5	0	1.000
				22.7%	0.0%	
			No	17	1	
				77.3%	100.0%	
**M**	rs3130048	BAG6	GVHD III-IV	matched	unmatched	
			Yes	3	3	1.000
				23.1%	27.3%	
			No	10	8	
				76.9%	72.7%	
**M**	rs2844464	BAG6	GVHD III-IV	matched	unmatched	
			Yes	6	0	0.130
				35.3%	0.0%	
			No	11	7	
				64.7%	100.0%	
**M**	rs2242656	BAG6	GVHD III-IV	matched	unmatched	
			Yes	6	0	0.277
				33.3%	0.0%	
			No	12	6	
				66.7%	100.0%	

**Source of DNA[a]**	**SNP**	**Gene**	**Outcome/ status**	**Number of patients**			**Model**	***p*-value**
**D**	rs11571315	CTLA4	GVHD III-IV	TT	CT	CC	Additive	0.337
			Yes	2	4	0	Dominant	0.357
				15.4%	40.0%	0.0%	Recessive	1.000
			No	11	6	1	Homozygous	1.000
				84.6%	60.0%	100.0%	Heterozygous	0.341
**D**	rs733618	CTLA4	GVHD III-IV	CC	CT	TT	Additive	0.853
			Yes	1	4	1	Dominant	1.000
				16.7%	28.6%	25.0%	Recessive	1.000
			No	5	10	3	Homozygous	1.000
				83.3%	71.4%	75.0%	Heterozygous	1.000

**D**	rs4553808	CTLA4	GVHD III-IV	AA	AG	GG	Additive	1.000
			Yes	5	1	0	Dominant	1.000
				23.8%	33.3%	0.0%	Recessive	NA
			No	16	2	0	Homozygous	NA
				76.2%	66.7%	0.0%	Heterozygous	1.000
**D**	rs11571316	CTLA4	GVHD III-IV	GG	AG	AA	Additive	0.665
			Yes	3	3	0	Dominant	0.665
				21.4%	30.0%	0.0%	Recessive	NA
			No	11	7	0	Homozygous	NA
				78.6%	70.0%	0.0%	Heterozygous	0.665
**D**	rs62182595	CTLA4	GVHD III-IV	GG	AG	AA	Additive	1.000
			Yes	5	1	0	Dominant	1.000
				23.8%	33.3%	0.0%	Recessive	NA
			No	16	2	0	Homozygous	NA
				76.2%	66.7%	0.0%	Heterozygous	1.000
**D**	rs573554201	CTLA4	GVHD III-IV	AA	AG	GG	Additive	NA
			Yes	6	0	0	Dominant	NA
				25.0%	0.0%	0.0%	Recessive	NA
			No	18	0	0	Homozygous	NA
				75.0%	0.0%	0.0%	Heterozygous	NA
**D**	rs16840252	CTLA4	GVHD III-IV	CC	CT	TT	Additive	1.000
			Yes	5	1	0	Dominant	1.000
				23.8%	33.3%	0.0%	Recessive	NA
			No	16	2	0	Homozygous	NA
				76.2%	66.7%	0.0%	Heterozygous	1.000
**D**	rs945677329	CTLA4	GVHD III-IV	AA	AG	GG	Additive	1.000
			Yes	6	0	0	Dominant	1.000
				26.1%	0.0%	0.0%	Recessive	NA
			No	17	1	0	Homozygous	NA
				73.9%	100.0%	0.0%	Heterozygous	1.000
**D**	rs5742909	CTLA4	GVHD III-IV	CC	CT	TT	Additive	1.000
			Yes	5	1	0	Dominant	1.000
				26.3%	20.0%	0.0%	Recessive	NA
			No	14	4	0	Homozygous	NA
				73.7%	80.0%	0.0%	Heterozygous	1.000
**D**	rs231775	CTLA4	GVHD III-IV	GG	AG	AA	Additive	0.591
			Yes	2	4	0	Dominant	0.649
				18.2%	33.3%	0.0%	Recessive	1.000
			No	9	8	1	Homozygous	1.000
				81.8%	66.7%	100.0%	Heterozygous	0.640
**D**	rs56102377	CTLA4	GVHD III-IV	GG	AG	AA	Additive	0.446
			Yes	5	1	0	Dominant	0.446
				22.7%	50.0%	0.0%	Recessive	NA
			No	17	1	0	Homozygous	NA
				77.3%	50.0%	0.0%	Heterozygous	0.446
**D**	rs56217811	CTLA4	GVHD III-IV	CC	CA	AA	Additive	NA
			Yes	6	0	0	Dominant	NA
				25.0%	0.0%	0.0%	Recessive	NA

			No	18	0	0	Homozygous	NA
				75.0%	0.0%	0.0%	Heterozygous	NA
**D**	rs55696217	CTLA4	GVHD III-IV	GG	GA	AA	Additive	NA
			Yes	6	0	0	Dominant	NA
				25.0%	0.0%	0.0%	Recessive	NA
			No	18	0	0	Homozygous	NA
				75.0%	0.0%	0.0%	Heterozygous	NA
**D**	rs231721	CTLA4	GVHD III-IV	TT	CT	CC	Additive	NA
			Yes	6	0	0	Dominant	NA
				25.0%	0.0%	0.0%	Recessive	NA
			No	18	0	0	Homozygous	NA
				75.0%	0.0%	0.0%	Heterozygous	NA
**D**	rs778932058	CTLA4	GVHD III-IV	AA	AG	GG	Additive	NA
			Yes	6	0	0	Dominant	NA
				25.0%	0.0%	0.0%	Recessive	NA
			No	18	0	0	Homozygous	NA
				75.0%	0.0%	0.0%	Heterozygous	NA
**D**	rs3087243	CTLA4	GVHD III-IV	GG	AG	AA	Additive	1.000
			Yes	3	3	0	Dominant	1.000
				23.1%	27.3%	0.0%	Recessive	NA
			No	10	8	0	Homozygous	NA
				76.9%	72.7%	0.0%	Heterozygous	1.000
**D**	rs11571319	CTLA4	GVHD III-IV	GG	AG	AA	Additive	1.000
			Yes	5	1	0	Dominant	1.000
				25.0%	25.0%	0.0%	Recessive	NA
			No	15	3	0	Homozygous	NA
				75.0%	75.0%	0.0%	Heterozygous	1.000
**D**	rs1234314	TNFSF4	GVHD III-IV	CC	CG	GG	Additive	0.475
			Yes	1	4	1	Dominant	0.621
				12.5%	36.4%	20.0%	Recessive	1.000
			No	7	7	4	Homozygous	1.000
				87.5%	63.6%	80.0%	Heterozygous	0.338
**D**	rs45454293	TNFSF4	GVHD III-IV	CC	CT	TT	Additive	0.358
			Yes	3	3	0	Dominant	0.635
				20.0%	42.9%	0.0%	Recessive	1.000
			No	12	4	2	Homozygous	1.000
				80.0%	57.1%	100.0%	Heterozygous	0.334
**D**	rs181758110	TNFSF4	GVHD III-IV	CC	CT	TT	Additive	NA
			Yes	6	0	0	Dominant	NA
				25.0%	0.0%	0.0%	Recessive	NA
			No	18	0	0	Homozygous	NA
				75.0%	0.0%	0.0%	Heterozygous	NA
**D**	rs1879877	CD28	GVHD III-IV	TT	GT	GG	Additive	0.818
			Yes	2	4	0	Dominant	1.000
				25.0%	28.6%	0.0%	Recessive	1.000
			No	6	10	1	Homozygous	1.000
				75.0%	71.4%	100.0%	Heterozygous	0.623
**D**	rs3181096	CD28	GVHD III-IV	CC	CT	TT	Additive	0.643

			Yes	3	3	0	Dominant	0.643
				21.4%	33.3%	0.0%	Recessive	NA
			No	11	6	0	Homozygous	NA
				78.6%	66.7%	0.0%	Heterozygous	0.643
**D**	rs3181097	CD28	GVHD III-IV	AA	AG	GG	Additive	0.818
			Yes	2	3	1	Dominant	0.632
				33.3%	21.4%	33.3%	Recessive	1.000
			No	4	11	2	Homozygous	1.000
				66.7%	78.6%	66.7%	Heterozygous	0.613
**D**	rs3181098	CD28	GVHD III-IV	GG	AG	AA	Additive	0.643
			Yes	3	3	0	Dominant	0.643
				21.4%	33.3%	0.0%	Recessive	NA
			No	11	6	0	Homozygous	NA
				78.6%	66.7%	0.0%	Heterozygous	0.643
**D**	rs28718975	CD28	GVHD III-IV	TT	CT	CC	Additive	0.075
			Yes	5	0	1	Dominant	0.621
				31.3%	0.0%	100.0%	Recessive	0.261
			No	11	6	0	Homozygous	0.353
				68.8%	100.0%	0.0%	Heterozygous	0.266
**D**	rs28688913	CD28	GVHD III-IV	CC	CT	TT	Additive	0.621
			Yes	5	1	0	Dominant	0.621
				31.3%	14.3%	0.0%	Recessive	NA
			No	11	6	0	Homozygous	NA
				68.8%	85.7%	0.0%	Heterozygous	0.621
**D**	rs28541784	CD28	GVHD III-IV	CC	CT	TT	Additive	0.144
			Yes	6	0	0	Dominant	0.144
				35.3%	0.0%	0.0%	Recessive	NA
			No	11	6	0	Homozygous	NA
				64.7%	100.0%	0.0%	Heterozygous	0.144
**D**	rs201801072	CD28	GVHD III-IV	TT	CT	CC	Additive	0.621
			Yes	5	1	0	Dominant	0.621
				31.3%	14.3%	0.0%	Recessive	NA
			No	11	6	0	Homozygous	NA
				68.8%	85.7%	0.0%	Heterozygous	0.621
**D**	rs200353921	CD28	GVHD III-IV	TT	AT	AA	Additive	0.621
			Yes	5	1	0	Dominant	0.621
				31.3%	14.3%	0.0%	Recessive	NA
			No	11	6	0	Homozygous	NA
				68.8%	85.7%	0.0%	Heterozygous	0.621
**D**	rs56228674	CD28	GVHD III-IV	CC	CT	TT	Additive	0.462
			Yes	5	1	0	Dominant	0.462
				23.8%	50.0%	0.0%	Recessive	NA
			No	16	1	0	Homozygous	NA
				76.2%	50.0%	0.0%	Heterozygous	0.462
**D**	rs129018028	CD28	GVHD III-IV	TT	CT	CC	Additive	NA
			Yes	6	0	0	Dominant	NA
				26.1%	0.0%	0.0%	Recessive	NA
			No	17	0	0	Homozygous	NA
				73.9%	0.0%	0.0%	Heterozygous	NA

**D**	rs3116496	CD28	GVHD III-IV	TT	CT	CC	Additive	0.462
			Yes	5	1	0	Dominant	0.462
				23.8%	50.0%	0.0%	Recessive	NA
			No	16	1	0	Homozygous	NA
				76.2%	50.0%	0.0%	Heterozygous	0.462
**D**	rs375758971	CD28	GVHD III-IV	TT	CT	CC	Additive	NA
			Yes	6	0	0	Dominant	NA
				26.1%	0.0%	0.0%	Recessive	NA
			No	17	0	0	Homozygous	NA
				73.9%	0.0%	0.0%	Heterozygous	NA
**D**	rs36084323	PD1	GVHD III-IV	CC	CT	TT	Additive	0.762
			Yes	2	2	2	Dominant	1.000
				28.6%	18.2%	33.3%	Recessive	0.618
			No	5	9	4	Homozygous	1.000
				71.4%	81.8%	66.7%	Heterozygous	1.000
**D**	rs5839828	PD1	GVHD III-IV	del	delG	GG	Additive	0.427
			Yes	3	2	1	Dominant	0.307
				42.9%	16.7%	20.0%	Recessive	1.000
			No	4	10	4	Homozygous	0.576
				57.1%	83.3%	80.0%	Heterozygous	0.305
**D**	rs11568821	PD1	GVHD III-IV	CC	CA	AA	Additive	NA
			Yes	6	0	0	Dominant	NA
				25.0%	0.0%	0.0%	Recessive	NA
			No	18	0	0	Homozygous	NA
				75.0%	0.0%	0.0%	Heterozygous	NA
**D**	rs41386349	PD1	GVHD III-IV	GG	AG	AA	Additive	0.184
			Yes	3	2	1	Dominant	0.362
				18.8%	28.6%	100.0%	Recessive	0.250
			No	13	5	0	Homozygous	0.235
				81.3%	71.4%	0.0%	Heterozygous	0.621
**D**	rs6705653	PD1	GVHD III-IV	CC	CT	TT	Additive	0.195
			Yes	3	2	1	Dominant	1.000
				25.0%	18.2%	100.0%	Recessive	0.250
			No	9	9	0	Homozygous	1.000
				75.0%	81.8%	0.0%	Heterozygous	1000
**D**	rs2227982	PD1	GVHD III-IV	GG	AG	AA	Additive	0.296
			Yes	2	1	3	Dominant	1.000
				28.6%	10.0%	42.9%	Recessive	0.307
			No	5	9	4	Homozygous	1.000
				71.4%	90.0%	57.1%	Heterozygous	0.537

[a]P: patient; D: donor; M: patient and donor mismatch status.
