# Supplementary material for: Preliminary Data on SNP of Transplantation-Related Genes after Haploidentical Stem Cell Transplantation
Source: J Clin Med. 2024 Aug 9;13(16):4681. doi: 10.3390/jcm13164681 (PMC11354871; doi:10.3390/jcm13164681)
Supplement: Supplementary file 1 [file jcm-13-04681-s001.zip › Supplementary Table 7_20240718.pdf]

Table S7. Association analysis between SNPs and cGVHD status post-haplo-HSCT.

Source of DNA[a]	SNP	Gene	Outcome/ status	Number of patients			Model	*p*-value
**P**	rs9276982	HLA-DOA	CGVHD	GG	AG	AA	Additive	1.000
			Yes	3	1	0	Dominant	1.000
				18.8%	12.5%	0.0%	Recessive	NA
			No	13	7	0	Homozygous	NA
				81.3%	87.5%	0.0%	Heterozygous	1.000
**P**	rs71565361	HLA-DOA	CGVHD	CC	CA	AA	Additive	1
			Yes	4	0	0	Dominant	1
				17.4%	0.0%	0.0%	Recessive	NA
			No	19	1	0	Homozygous	NA
				82.6%	100.0%	0.0%	Heterozygous	1
**P**	rs79327197	HLA-DOA	CGVHD	AA	AG	GG	Additive	1
			Yes	4	0	0	Dominant	1
				20.0%	0.0%	0.0%	Recessive	NA
			No	16	4	0	Homozygous	NA
				80.0%	100.0%	0.0%	Heterozygous	1
**P**	rs151190962	HLA-DOA	CGVHD	AA	delA	del	Additive	1.000
			Yes	4	0	0	Dominant	1.000
				17.4%	0.0%	0.0%	Recessive	NA
			No	19	1	0	Homozygous	NA
				82.6%	100.0%	0.0%	Heterozygous	1.000
**P**	rs9282369	HLA-DOA	CGVHD	ins	insT	TT	Additive	0.819
			Yes	2	1	1	Dominant	0.615
				22.2%	11.1%	16.7%	Recessive	1.000
			No	7	8	5	Homozygous	1.000
				77.8%	88.9%	83.3%	Heterozygous	1.000
**P**	rs2009658	LTA	CGVHD	CC	CG	GG	Additive	0.578
			Yes	2	2	0	Dominant	0.578
				12.5%	25.0%	0.0%	Recessive	NA
			No	14	6	0	Homozygous	NA
				87.5%	75.0%	0.0%	Heterozygous	0.578
**P**	rs111250247	LTA	CGVHD	TT	TA	AA	Additive	NA
			Yes	4	0	0	Dominant	NA
				16.7%	0.0%	0.0%	Recessive	NA
			No	20	0	0	Homozygous	NA
				83.3%	0.0%	0.0%	Heterozygous	NA
**P**	rs915654	LTA	CGVHD	AA	TA	TT	Additive	0.657
			Yes	1	2	1	Dominant	1.000
				20.0%	12.5%	33.3%	Recessive	0.437
			No	4	14	2	Homozygous	1.000
				80.0%	87.5%	66.7%	Heterozygous	1.000
**P**	rs34324979	HSPA1L	CGVHD	GG	Gins	ins	Additive	1.000
			Yes	3	1	0	Dominant	1.000
				16.7%	16.7%	0.0%	Recessive	NA
			No	15	5	0	Homozygous	NA
				83.3%	83.3%	0.0%	Heterozygous	1.000
**P**	rs2075800	HSPA1L	CGVHD	CC	CT	TT	Additive	0.597927533

			Yes	1	3	0	Dominant	1.000
				16.7%	21.4%	0.0%	Recessive	1.000
			No	5	11	4	Homozygous	1.000
				83.3%	78.6%	100.0%	Heterozygous	1.000
**P**	rs2227956	HSPA1L	CGVHD	AA	GA	GG	Additive	0.544
			Yes	3	1	0	Dominant	0.544
				15.0%	25.0%	0.0%	Recessive	NA
			No	17	3	0	Homozygous	NA
				85.0%	75.0%	0.0%	Heterozygous	0.544
**P**	rs107822	RING1	CGVHD	TT	CT	CC	Additive	0.778
			Yes	3	1	0	Dominant	1.000
				21.4%	12.5%	0.0%	Recessive	1.000
			No	11	7	1	Homozygous	1.000
				78.6%	87.5%	100.0%	Heterozygous	1.000
**P**	rs213210	RING1	CGVHD	GG	AG	AA	Additive	0.687
			Yes	3	1	0	Dominant	0.604
				23.1%	11.1%	0.0%	Recessive	1.000
			No	10	8	1	Homozygous	1.000
				76.9%	88.9%	100.0%	Heterozygous	0.616
**P**	rs209132	TRIM27	CGVHD	GG	AG	AA	Additive	0.602
			Yes	2	2	0	Dominant	1.000
				15.4%	25.0%	0.0%	Recessive	1.000
			No	11	6	3	Homozygous	1.000
				84.6%	75.0%	100.0%	Heterozygous	0.618
**P**	rs209131	TRIM27	CGVHD	GG	AG	AA	Additive	0.284
			Yes	1	1	2	Dominant	1.000
				12.5%	9.1%	40.0%	Recessive	0.179
			No	7	10	3	Homozygous	0.510
				87.5%	90.9%	60.0%	Heterozygous	1.000
**P**	rs209130	TRIM27	CGVHD	TT	CT	CC	Additive	0.735
			Yes	2	2	0	Dominant	1.000
				15.4%	22.2%	0.0%	Recessive	1.000
			No	11	7	2	Homozygous	1.000
				84.6%	77.8%	100.0%	Heterozygous	1.000
**P**	rs1536215	TRIM27	CGVHD	CC	CG	GG	Additive	1.000
			Yes	3	1	0	Dominant	1.000
				17.6%	14.3%	0.0%	Recessive	NA
			No	14	6	0	Homozygous	NA
				82.4%	85.7%	0.0%	Heterozygous	1.000
**P**	rs139791445	TRIM27	CGVHD	CC	CG	GG	Additive	NA
			Yes	4	0	0	Dominant	NA
				16.7%	0.0%	0.0%	Recessive	NA
			No	20	0	0	Homozygous	NA
				83.3%	0.0%	0.0%	Heterozygous	NA
**P**	rs11244	HLA-DOB	CGVHD	GG	AG	AA	Additive	0.445
			Yes	2	1	1	Dominant	1.000
				14.3%	14.3%	50.0%	Recessive	0.324
			No	12	6	1	Homozygous	0.350
				85.7%	85.7%	50.0%	Heterozygous	1.000

**P**	rs2070120	HLA-DOB	CGVHD	GG	AG	AA	Additive	0.453
			Yes	3	1	0	Dominant	0.453
				15.0%	33.3%	0.0%	Recessive	NA
			No	17	2	0	Homozygous	NA
				85.0%	66.7%	0.0%	Heterozygous	0.453
**P**	rs56150445	HLA-DOB	CGVHD	GG	CG	CC	Additive	NA
			Yes	4	0	0	Dominant	NA
				17.4%	0.0%	0.0%	Recessive	NA
			No	19	0	0	Homozygous	NA
				82.6%	0.0%	0.0%	Heterozygous	NA
**P**	rs41258084	HLA-DOB	CGVHD	CC	CT	TT	Additive	1.000
			Yes	4	0	0	Dominant	1.000
				19.0%	0.0%	0.0%	Recessive	NA
			No	17	2	0	Homozygous	NA
				81.0%	100.0%	0.0%	Heterozygous	1.000
**P**	rs17220087	HLA-DOB	CGVHD	CC	AC	AA	Additive	0.453
			Yes	3	1	0	Dominant	0.453
				15.0%	33.3%	0.0%	Recessive	NA
			No	17	2	0	Homozygous	NA
				85.0%	66.7%	0.0%	Heterozygous	0.453
**P**	rs2071479	HLA-DOB	CGVHD	CC	CT	TT	Additive	1.000
			Yes	4	0	0	Dominant	1.000
				18.2%	0.0%	0.0%	Recessive	NA
			No	18	1	0	Homozygous	NA
				81.8%	100.0%	0.0%	Heterozygous	1.000
**P**	rs17213693	HLA-DOB	CGVHD	GG	CG	CC	Additive	0.125
			Yes	2	2	0	Dominant	0.125
				10.5%	50.0%	0.0%	Recessive	NA
			No	17	2	0	Homozygous	NA
				89.5%	50.0%	0.0%	Heterozygous	0.125
**P**	rs3130048	BAG6	CGVHD	TT	CT	CC	Additive	0.730
			Yes	3	1	0	Dominant	0.615
				21.4%	11.1%	0.0%	Recessive	1.000
			No	11	8	1	Homozygous	1.000
				78.6%	88.9%	100.0%	Heterozygous	1.000
**P**	rs2844464	BAG6	CGVHD	GG	AG	AA	Additive	0.278
			Yes	2	2	0	Dominant	0.251
				11.1%	40.0%	0.0%	Recessive	1.000
			No	16	3	1	Homozygous	1.000
				88.9%	60.0%	100.0%	Heterozygous	0.194
**P**	rs2242656	BAG6	CGVHD	TT	CT	CC	Additive	0.179
			Yes	2	2	0	Dominant	0.179
				10.5%	40.0%	0.0%	Recessive	NA
			No	17	3	0	Homozygous	NA
				89.5%	60.0%	0.0%	Heterozygous	0.179
**D**	rs61365987	NOTCH4	CGVHD	CC	CT	TT	Additive	0.437
			Yes	3	1	0	Dominant	0.437
				14.3%	33.3%	0.0%	Recessive	NA
			No	18	2	0	Homozygous	NA
				85.7%	66.7%	0.0%	Heterozygous	0.437

**D**	rs444472	NOTCH4	CGVHD	GG	GA	AA	Additive	0.073
			Yes	2	1	1	Dominant	0.578
				12.5%	14.3%	100.0%	Recessive	0.167
			No	14	6	0	Homozygous	0.176
				87.5%	85.7%	0.0%	Heterozygous	1.000
**D**	rs2256594	NOTCH4	CGVHD	AA	AG	GG	Additive	0.545
			Yes	1	3	0	Dominant	1.000
				14.3%	23.1%	0.0%	Recessive	1.000
			No	6	10	4	Homozygous	1.000
				85.7%	76.9%	100.0%	Heterozygous	1.000
**D**	rs394657	NOTCH4	CGVHD	AA	AG	GG	Additive	0.073
			Yes	2	1	1	Dominant	0.578
				12.5%	14.3%	100.0%	Recessive	0.167
			No	14	6	0	Homozygous	0.176
				87.5%	85.7%	0.0%	Heterozygous	1.000
**D**	rs429853	NOTCH4	CGVHD	TT	TC	CC	Additive	0.552
			Yes	2	2	0	Dominant	0.552
				11.8%	28.6%	0.0%	Recessive	NA
			No	15	5	0	Homozygous	NA
				88.2%	71.4%	0.0%	Heterozygous	0.552
**D**	rs111394117	NOTCH4	CGVHD	GG	GA	AA	Additive	1.000
			Yes	4	0	0	Dominant	1.000
				19.0%	0.0%	0.0%	Recessive	NA
			No	17	3	0	Homozygous	NA
				81.0%	100.0%	0.0%	Heterozygous	1.000
**D**	rs9281491	HCP5	CGVHD	ins	insA	AA	Additive	0.900
			Yes	3	1	0	Dominant	1.000
				17.6%	16.7%	0.0%	Recessive	1.000
			No	14	5	1	Homozygous	1.000
				82.4%	83.3%	100.0%	Heterozygous	1.000
**D**	rs2244546	HCP5	CGVHD	CC	CG	GG	Additive	1.000
			Yes	3	1	0	Dominant	1.000
				18.8%	12.5%	0.0%	Recessive	NA
			No	13	7	0	Homozygous	NA
				81.3%	87.5%	0.0%	Heterozygous	1.000
**D**	rs4713466	HCP5	CGVHD	CC	CT	TT	Additive	0.596
			Yes	3	1	0	Dominant	0.596
				23.1%	9.1%	0.0%	Recessive	NA
			No	10	10	0	Homozygous	NA
				76.9%	90.9%	0.0%	Heterozygous	0.596
**D**	rs2523676	HCP5	CGVHD	CC	CT	TT	Additive	0.320
			Yes	2	1	1	Dominant	1.000
				20.0%	8.3%	50.0%	Recessive	0.312
			No	8	11	1	Homozygous	0.455
				80.0%	91.7%	50.0%	Heterozygous	0.571
**D**	rs2523675	HCP5	CGVHD	GG	GA	AA	Additive	0.383
			Yes	0	3	1	Dominant	0.539
				0.0%	20.0%	33.3%	Recessive	0.437
			No	6	12	2	Homozygous	0.333

				100.0%	80.0%	66.7%	Heterozygous	0.526
**D**	rs2518028	HCP5	CGVHD	CC	TC	TT	Additive	1.000
			Yes	3	1	0	Dominant	1.000
				15.8%	20.0%	0.0%	Recessive	NA
			No	16	4	0	Homozygous	NA
				84.2%	80.0%	0.0%	Heterozygous	1.000
**D**	rs141431529	HCP5	CGVHD	GG	GT	TT	Additive	0.544
			Yes	3	1	0	Dominant	0.544
				15.0%	25.0%	0.0%	Recessive	NA
			No	17	3	0	Homozygous	NA
				85.0%	75.0%	0.0%	Heterozygous	0.544
**D**	rs107822	RING1	CGVHD	TT	CT	CC	Additive	**0.018**
			Yes	0	4	0	Dominant	0.093
				0.0%	44.4%	0.0%	Recessive	1.000
			No	12	5	3	Homozygous	NA
				100.0%	55.6%	100.0%	Heterozygous	**0.021**
**D**	rs213210	RING1	CGVHD	GG	AG	AA	Additive	0.147
			Yes	0	2	2	Dominant	0.259
				0.0%	20.0%	40.0%	Recessive	0.179
			No	9	8	3	Homozygous	0.110
				100.0%	80.0%	60.0%	Heterozygous	0.474
**D**	rs209132	TRIM27	CGVHD	GG	AG	AA	Additive	0.418
			Yes	3	1	0	Dominant	0.300
				27.3%	9.1%	0.0%	Recessive	1.000
			No	8	10	2	Homozygous	1.000
				72.7%	90.9%	100.0%	Heterozygous	0.586
**D**	rs209131	TRIM27	CGVHD	GG	AG	AA	Additive	0.549
			Yes	2	1	1	Dominant	0.578
				25.0%	8.3%	25.0%	Recessive	0.544
			No	6	11	3	Homozygous	1.000
				75.0%	91.7%	75.0%	Heterozygous	0.537
**D**	rs209130	TRIM27	CGVHD	TT	CT	CC	Additive	0.534
			Yes	3	1	0	Dominant	0.590
				25.0%	9.1%	0.0%	Recessive	1.000
			No	9	10	1	Homozygous	1.000
				75.0%	90.9%	100.0%	Heterozygous	0.590
**D**	rs1536215	TRIM27	CGVHD	CC	CG	GG	Additive	0.552
			Yes	2	2	0	Dominant	0.552
				11.8%	28.6%	0.0%	Recessive	NA
			No	15	5	0	Homozygous	NA
				88.2%	71.4%	0.0%	Heterozygous	0.552
**D**	rs139791445	TRIM27	CGVHD	CC	CG	GG	Additive	1.000
			Yes	4	0	0	Dominant	1.000
				17.4%	0.0%	0.0%	Recessive	NA
			No	19	1	0	Homozygous	NA
				82.6%	100.0%	0.0%	Heterozygous	1.000
**D**	rs11244	HLA-DOB	CGVHD	GG	AG	AA	Additive	**0.046**
			Yes	1	1	2	Dominant	0.615
				10.0%	9.1%	66.7%	Recessive	0.061

			No	9	10	1	Homozygous	0.108
				90.0%	90.9%	33.3%	Heterozygous	1.000
**D**	rs2070120	HLA-DOB	CGVHD	GG	AG	AA	Additive	1.000
			Yes	4	0	0	Dominant	1.000
				17.4%	0.0%	0.0%	Recessive	NA
			No	19	1	0	Homozygous	NA
				82.6%	100.0%	0.0%	Heterozygous	1.000
**D**	rs56150445	HLA-DOB	CGVHD	GG	CG	CC	Additive	NA
			Yes	4	0	0	Dominant	NA
				16.7%	0.0%	0.0%	Recessive	NA
			No	20	0	0	Homozygous	NA
				83.3%	0.0%	0.0%	Heterozygous	NA
**D**	rs41258084	HLA-DOB	CGVHD	CC	CT	TT	Additive	1.000
			Yes	4	0	0	Dominant	1.000
				18.2%	0.0%	0.0%	Recessive	NA
			No	18	2	0	Homozygous	NA
				81.8%	100.0%	0.0%	Heterozygous	1.000
**D**	rs17220087	HLA-DOB	CGVHD	CC	AC	AA	Additive	1.000
			Yes	4	0	0	Dominant	1.000
				17.4%	0.0%	0.0%	Recessive	NA
			No	19	1	0	Homozygous	NA
				82.6%	100.0%	0.0%	Heterozygous	1.000
**D**	rs2071479	HLA-DOB	CGVHD	CC	CT	TT	Additive	1.000
			Yes	4	0	0	Dominant	1.000
				17.4%	0.0%	0.0%	Recessive	NA
			No	19	1	0	Homozygous	NA
				82.6%	100.0%	0.0%	Heterozygous	1.000
**D**	rs17213693	HLA-DOB	CGVHD	GG	CG	CC	Additive	1.000
			Yes	4	0	0	Dominant	1.000
				17.4%	0.0%	0.0%	Recessive	NA
			No	19	1	0	Homozygous	NA
				82.6%	100.0%	0.0%	Heterozygous	1.000
**D**	rs3130048	BAG6	CGVHD	TT	CT	CC	Additive	0.598
			Yes	3	1	0	Dominant	0.615
				21.4%	16.7%	0.0%	Recessive	1.000
			No	11	5	4	Homozygous	1.000
				78.6%	83.3%	100.0%	Heterozygous	1.000
**D**	rs2844464	BAG6	CGVHD	GG	AG	AA	Additive	0.179
			Yes	2	2	0	Dominant	0.179
				10.5%	40.0%	0.0%	Recessive	NA
			No	17	3	0	Homozygous	NA
				89.5%	60.0%	0.0%	Heterozygous	0.179
**D**	rs2242656	BAG6	CGVHD	TT	CT	CC	Additive	0.179
			Yes	2	2	0	Dominant	0.179
				10.5%	40.0%	0.0%	Recessive	NA
			No	17	3	0	Homozygous	NA
				89.5%	60.0%	0.0%	Heterozygous	0.179
**M**	rs107822	RING1	CGVHD		matched		unmatched	0.590
			Yes		1		3	

				9.1%	25.0%	
			No	10	9	
				90.9%	75.0%	
**M**	rs213210	RING1	CGVHD	matched	unmatched	1.000
			Yes	1	3	
				14.3%	18.8%	
			No	6	13	
				85.7%	81.3%	
**M**	rs209132	TRIM27	CGVHD	matched	unmatched	0.130
			Yes	1	3	
				6.7%	33.3%	
			No	14	6	
				93.3%	66.7%	
**M**	rs209131	TRIM27	CGVHD	matched	unmatched	0.272
			Yes	1	3	
				7.1%	30.0%	
			No	13	7	
				92.9%	70.0%	
**M**	rs209130	TRIM27	CGVHD	matched	unmatched	0.272
			Yes	1	3	
				7.1%	30.0%	
			No	13	7	
				92.9%	70.0%	
**M**	rs1536215	TRIM27	CGVHD	matched	unmatched	0.544
			Yes	3	1	
				15.0%	25.0%	
			No	17	3	
				85.0%	75.0%	
**M**	rs139791445	TRIM27	CGVHD	matched	unmatched	1.000
			Yes	4	0	
				17.4%	0.0%	
			No	19	1	
				82.6%	100.0%	
**M**	rs11244	HLA-DOB	CGVHD	matched	unmatched	1.000
			Yes	2	2	
				15.4%	20.0%	
			No	11	8	
				84.6%	80.0%	
**M**	rs2070120	HLA-DOB	CGVHD	matched	unmatched	0.174
			Yes	3	1	
				13.6%	100.0%	
			No	19	0	
				86.4%	0.0%	
**M**	rs56150445	HLA-DOB	CGVHD	matched	unmatched	NA
			Yes	4	0	
				17.4%	0.0%	
			No	19	0	
				82.6%	0.0%	

**M**	rs41258084	HLA-DOB	CGVHD	matched	unmatched	NA
			Yes	4	0	
				17.4%	0.0%	
			No	19	0	
				82.6%	0.0%	
**M**	rs17220087	HLA-DOB	CGVHD	matched	unmatched	0.324
			Yes	3	1	
				14.3%	50.0%	
			No	18	1	
				85.7%	50.0%	
**M**	rs2071479	HLA-DOB	CGVHD	matched	unmatched	1.000
			Yes	4	0	
				18.2%	0.0%	
			No	18	1	
				81.8%	100.0%	
**M**	rs17213693	HLA-DOB	CGVHD	matched	unmatched	1.000
			Yes	3	1	
				13.6%	100.0%	
			No	19	0	
				86.4%	0.0%	
**M**	rs3130048	BAG6	CGVHD	matched	unmatched	0.098
			Yes	4	0	
				30.8%	0.0%	
			No	9	11	
				69.2%	100.0%	
**M**	rs2844464	BAG6	CGVHD	matched	unmatched	0.552
			Yes	2	2	
				11.8%	28.6%	
			No	15	5	
				88.2%	71.4%	
**M**	rs2242656	BAG6	CGVHD	matched	unmatched	0.251
			Yes	2	2	
				11.1%	33.3%	
			No	16	4	
				88.9%	66.7%	

**Source of DNA[a]**	**SNP**	**Gene**	**Outcome/ status**	**Number of patients**			**Model**	***p*-value**
**D**	rs11571315	CTLA4	CGVHD	TT	CT	CC	Additive	0.863
			Yes	2	2	0	Dominant	1.000
				15.4%	20.0%	0.0%	Recessive	1.000
			No	11	8	1	Homozygous	1.000
				84.6%	80.0%	100.0%	Heterozygous	1.000
**D**	rs733618	CTLA4	CGVHD	CC	CT	TT	Additive	0.443
			Yes	0	3	1	Dominant	0.539
				0.0%	21.4%	25.0%	Recessive	0.544
			No	6	11	3	Homozygous	0.539
				100.0%	78.6%	75.0%	Heterozygous	0.521
**D**	rs4553808	CTLA4	CGVHD	AA	AG	GG	Additive	0.437
			Yes	3	1	0	Dominant	0.437

				14.3%	33.3%	0.0%	Recessive	NA
			No	18	2	0	Homozygous	NA
				85.7%	66.7%	0.0%	Heterozygous	0.437
**D**	rs11571316	CTLA4	CGVHD	GG	AG	AA	Additive	0.615
			Yes	3	1	0	Dominant	0.615
				21.4%	10.0%	0.0%	Recessive	NA
			No	11	9	0	Homozygous	NA
				78.6%	90.0%	0.0%	Heterozygous	0.615
**D**	rs62182595	CTLA4	CGVHD	GG	AG	AA	Additive	0.437
			Yes	3	1	0	Dominant	0.437
				14.3%	33.3%	0.0%	Recessive	NA
			No	18	2	0	Homozygous	NA
				85.7%	66.7%	0.0%	Heterozygous	0.437
**D**	rs573554201	CTLA4	CGVHD	AA	AG	GG	Additive	NA
			Yes	4	0	0	Dominant	NA
				16.7%	0.0%	0.0%	Recessive	NA
			No	20	0	0	Homozygous	NA
				83.3%	0.0%	0.0%	Heterozygous	NA
**D**	rs16840252	CTLA4	CGVHD	CC	CT	TT	Additive	0.437
			Yes	3	1	0	Dominant	0.437
				14.3%	33.3%	0.0%	Recessive	NA
			No	18	2	0	Homozygous	NA
				85.7%	66.7%	0.0%	Heterozygous	0.437
**D**	rs945677329	CTLA4	CGVHD	AA	AG	GG	Additive	1.000
			Yes	4	0	0	Dominant	1.000
				17.4%	0.0%	0.0%	Recessive	NA
			No	19	1	0	Homozygous	NA
				82.6%	100.0%	0.0%	Heterozygous	1.000
**D**	rs5742909	CTLA4	CGVHD	CC	CT	TT	Additive	1.000
			Yes	3	1	0	Dominant	1.000
				15.8%	20.0%	0.0%	Recessive	NA
			No	16	4	0	Homozygous	NA
				84.2%	80.0%	0.0%	Heterozygous	1.000
**D**	rs231775	CTLA4	CGVHD	GG	AG	AA	Additive	0.897
			Yes	2	2	0	Dominant	1.000
				18.2%	16.7%	0.0%	Recessive	1.000
			No	9	10	1	Homozygous	1.000
				81.8%	83.3%	100.0%	Heterozygous	1.000
**D**	rs56102377	CTLA4	CGVHD	GG	AG	AA	Additive	1.000
			Yes	4	0	0	Dominant	1.000
				18.2%	0.0%	0.0%	Recessive	NA
			No	18	2	0	Homozygous	NA
				81.8%	100.0%	0.0%	Heterozygous	1.000
**D**	rs56217811	CTLA4	CGVHD	CC	CA	AA	Additive	NA
			Yes	4	0	0	Dominant	NA
				16.7%	0.0%	0.0%	Recessive	NA
			No	20	0	0	Homozygous	NA
				83.3%	0.0%	0.0%	Heterozygous	NA
**D**	rs55696217	CTLA4	CGVHD	GG	GA	AA	Additive	NA

			Yes	4	0	0	Dominant	NA
				16.7%	0.0%	0.0%	Recessive	NA
			No	20	0	0	Homozygous	NA
				83.3%	0.0%	0.0%	Heterozygous	NA
**D**	rs231721	CTLA4	CGVHD	TT	CT	CC	Additive	NA
			Yes	4	0	0	Dominant	NA
				16.7%	0.0%	0.0%	Recessive	NA
			No	20	0	0	Homozygous	NA
				83.3%	0.0%	0.0%	Heterozygous	NA
**D**	rs778932058	CTLA4	CGVHD	AA	AG	GG	Additive	NA
			Yes	4	0	0	Dominant	NA
				16.7%	0.0%	0.0%	Recessive	NA
			No	20	0	0	Homozygous	NA
				83.3%	0.0%	0.0%	Heterozygous	NA
**D**	rs3087243	CTLA4	CGVHD	GG	AG	AA	Additive	1.000
			Yes	2	2	0	Dominant	1.000
				15.4%	18.2%	0.0%	Recessive	NA
			No	11	9	0	Homozygous	NA
				84.6%	81.8%	0.0%	Heterozygous	1.000
**D**	rs11571319	CTLA4	CGVHD	GG	AG	AA	Additive	0.544
			Yes	3	1	0	Dominant	0.544
				15.0%	25.0%	0.0%	Recessive	NA
			No	17	3	0	Homozygous	NA
				85.0%	75.0%	0.0%	Heterozygous	0.544
**D**	rs1234314	TNFSF4	CGVHD	CC	CG	GG	Additive	0.639
			Yes	2	1	1	Dominant	0.578
				25.0%	9.1%	20.0%	Recessive	1.000
			No	6	10	4	Homozygous	1.000
				75.0%	90.9%	80.0%	Heterozygous	0.546
**D**	rs45454293	TNFSF4	CGVHD	CC	CT	TT	Additive	0.760
			Yes	3	1	0	Dominant	1.000
				20.0%	14.3%	0.0%	Recessive	1.000
			No	12	6	2	Homozygous	1.000
				80.0%	85.7%	100.0%	Heterozygous	1.000
**D**	rs181758110	TNFSF4	CGVHD	CC	CT	TT	Additive	NA
			Yes	4	0	0	Dominant	NA
				16.7%	0.0%	0.0%	Recessive	NA
			No	20	0	0	Homozygous	NA
				83.3%	0.0%	0.0%	Heterozygous	NA
**D**	rs1879877	CD28	CGVHD	TT	GT	GG	Additive	0.330
			Yes	0	3	0	Dominant	0.526
				0.0%	21.4%	0.0%	Recessive	1.000
			No	8	11	1	Homozygous	NA
				100.0%	78.6%	100.0%	Heterozygous	0.273
**D**	rs3181096	CD28	CGVHD	CC	CT	TT	Additive	0.538
			Yes	1	2	0	Dominant	0.538
				7.1%	22.2%	0.0%	Recessive	NA
			No	13	7	0	Homozygous	NA
				92.9%	77.8%	0.0%	Heterozygous	0.538

**D**	rs3181097	CD28	CGVHD	AA	AG	GG	Additive	0.330
			Yes	0	3	0	Dominant	0.539
				0.0%	21.4%	0.0%	Recessive	1.000
			No	6	11	3	Homozygous	NA
				100.0%	78.6%	100.0%	Heterozygous	0.521
**D**	rs3181098	CD28	CGVHD	GG	AG	AA	Additive	0.538
			Yes	1	2	0	Dominant	0.538
				7.1%	22.2%	0.0%	Recessive	NA
			No	13	7	0	Homozygous	NA
				92.9%	77.8%	0.0%	Heterozygous	0.538
**D**	rs28718975	CD28	CGVHD	TT	CT	CC	Additive	0.894
			Yes	2	1	0	Dominant	1.000
				12.5%	16.7%	0.0%	Recessive	1.000
			No	14	5	1	Homozygous	1.000
				87.5%	83.3%	100.0%	Heterozygous	1.000
**D**	rs28688913	CD28	CGVHD	CC	CT	TT	Additive	1.000
			Yes	2	1	0	Dominant	1.000
				12.5%	14.3%	0.0%	Recessive	NA
			No	14	6	0	Homozygous	NA
				87.5%	85.7%	0.0%	Heterozygous	1.000
**D**	rs28541784	CD28	CGVHD	CC	CT	TT	Additive	1.000
			Yes	2	1	0	Dominant	1.000
				11.8%	16.7%	0.0%	Recessive	NA
			No	15	5	0	Homozygous	NA
				88.2%	83.3%	0.0%	Heterozygous	1.000
**D**	rs201801072	CD28	CGVHD	TT	CT	CC	Additive	1.000
			Yes	2	1	0	Dominant	1.000
				12.5%	14.3%	0.0%	Recessive	NA
			No	14	6	0	Homozygous	NA
				87.5%	85.7%	0.0%	Heterozygous	1.000
**D**	rs200353921	CD28	CGVHD	TT	AT	AA	Additive	1.000
			Yes	2	1	0	Dominant	1.000
				12.5%	14.3%	0.0%	Recessive	NA
			No	14	6	0	Homozygous	NA
				87.5%	85.7%	0.0%	Heterozygous	1.000
**D**	rs56228674	CD28	CGVHD	CC	CT	TT	Additive	1.000
			Yes	3	0	0	Dominant	1.000
				14.3%	0.0%	0.0%	Recessive	NA
			No	18	2	0	Homozygous	NA
				85.7%	100.0%	0.0%	Heterozygous	1.000
**D**	rs1290180288	CD28	CGVHD	TT	CT	CC	Additive	NA
			Yes	3	0	0	Dominant	NA
				13.0%	0.0%	0.0%	Recessive	NA
			No	20	0	0	Homozygous	NA
				87.0%	0.0%	0.0%	Heterozygous	NA
**D**	rs3116496	CD28	CGVHD	TT	CT	CC	Additive	1.000
			Yes	3	0	0	Dominant	1.000
				14.3%	0.0%	0.0%	Recessive	NA
			No	18	2	0	Homozygous	NA
				85.7%	100.0%	0.0%	Heterozygous	1.000

**D**	rs375758971	CD28	CGVHD	TT	CT	CC	Additive	NA
			Yes	3	0	0	Dominant	NA
				13.0%	0.0%	0.0%	Recessive	NA
			No	20	0	0	Homozygous	NA
				87.0%	0.0%	0.0%	Heterozygous	NA
**D**	rs36084323	PD1	CGVHD	CC	CT	TT	Additive	0.557
			Yes	2	1	1	Dominant	0.552
				28.6%	9.1%	16.7%	Recessive	1.000
			No	5	10	5	Homozygous	1.000
				71.4%	90.9%	83.3%	Heterozygous	0.528
**D**	rs5839828	PD1	CGVHD	del	delG	GG	Additive	0.274
			Yes	1	1	2	Dominant	1.000
				14.3%	8.3%	40.0%	Recessive	0.179
			No	6	11	3	Homozygous	0.523
				85.7%	91.7%	60.0%	Heterozygous	1.000
**D**	rs11568821	PD1	CGVHD	CC	CA	AA	Additive	NA
			Yes	4	0	0	Dominant	NA
				16.7%	0.0%	0.0%	Recessive	NA
			No	20	0	0	Homozygous	NA
				83.3%	0.0%	0.0%	Heterozygous	NA
**D**	rs41386349	PD1	CGVHD	GG	AG	AA	Additive	0.301
			Yes	4	0	0	Dominant	1.000
				25.0%	0.0%	0.0%	Recessive	0.262
			No	12	7	1	Homozygous	1.000
				75.0%	100.0%	100.0%	Heterozygous	0.273
**D**	rs6705653	PD1	CGVHD	CC	CT	TT	Additive	0.534
			Yes	3	1	0	Dominant	0.590
				25.0%	9.1%	0.0%	Recessive	1.000
			No	9	10	1	Homozygous	1.000
				75.0%	90.9%	100.0%	Heterozygous	0.500
**D**	rs2227982	PD1	CGVHD	GG	AG	AA	Additive	0.588
			Yes	2	1	1	Dominant	0.552
				28.6%	10.0%	14.3%	Recessive	1.
			No	5	9	6	Homozygous	1.000
				71.4%	90.0%	85.7%	Heterozygous	0.537

[a]P: patient; D: donor; M: patient and donor mismatch status.
